# Efficacy of pharmacotherapies on pediatric patients with metabolic dysfunction-associated steatotic liver disease: a systematic review and network meta-analysis

**DOI:** 10.1186/s12876-025-04393-x

**Published:** 2025-12-08

**Authors:** Shehab Yaser, Hatem Yaser, Hazem E. Mohammed, Mohamed Nasser, Mohamed Khalafalla Darwish, Mohamed E. Haseeb, Anas Hussein Heiba, Mohamed Mabrouk Ghonaim, Heba Aboeldahab, Zeyad Bady

**Affiliations:** 1 Faculty of Medicine, Assiut University, Assiut, Egypt; 2 Medical Research Group of Egypt, Negida Academy, Arlington, MA USA; 3 Faculty of Medicine, Minia University, Minia, Egypt; 4 Faculty of Medicine, Menoufia university, Menoufia, Egypt; 5 Clinical Research Department, El-Gomhoria General Hospital, MOHP, Alexandria, Egypt

**Keywords:** Metabolic dysfunction-associated steatotic liver disease, Pediatric patients, Pharmacotherapies, NAFLD activity score systematic review, Network meta-analysis

## Abstract

**Background:**

Metabolic dysfunction-associated steatotic liver disease (MASLD) affects children and is increasingly prevalent alongside rising childhood obesity. The MASLD spectrum spans from simple hepatic steatosis to metabolic-associated steatohepatitis (MASH), fibrosis, and cirrhosis. Despite this rising prevalence, the optimal pharmacotherapy for pediatric MASLD remains uncertain.

**Objective:**

This systematic review and network meta-analysis aimed to evaluate the efficacy of different pharmacotherapies in managing pediatric MASLD.

**Methods:**

Included in this meta-analysis were randomized controlled trials. The diagnosis of MASLD was established using medical imaging techniques such as ultrasonography or magnetic resonance imaging, or via liver biopsy, provided that patients had no other chronic liver diseases or secondary causes of liver steatosis. A systematic search of five electronic databases was conducted up to August 2024. Data were synthesized using a random-effects model, with results expressed as pooled mean differences (MDs) for continuous outcomes or relative risks (RRs) for categorical outcomes, each with a 95% confidence interval (CI).

**Results:**

This analysis included 26 trials involving 1503 patients. The mean age of patients across studies ranged from 7.41 to 14.06 years. Vitamin D demonstrated the best ranking in managing MASLD, significantly reducing NAFLD Activity Score (NAS) and improving lipid profiles by reducing low-density lipoprotein (LDL) and total cholesterol, while increasing high-density lipoprotein (HDL) levels. Besides, vitamin D combined with docosahexaenoic acid (DHA) showed the strongest effect in reducing triglycerides. Vitamin E was associated with more patients achieving nonalcoholic steatohepatitis (NASH) resolution. Regarding liver transaminases, Cysteamine Bitartrate Delayed Release (CBDR) most effectively reduced ALT levels, AST levels, and fibrosis score.

**Conclusions:**

Our NMA suggests pharmacotherapy holds promise for pediatric MASLD, with vitamin D and vitamin E presenting the most consistent benefits across histologic and laboratory outcomes. Future well-designed RCTs integrating standardized MASLD diagnosis and lifestyle interventions are warranted.

**Supplementary Information:**

The online version contains supplementary material available at 10.1186/s12876-025-04393-x.

## Introduction

Metabolic dysfunction-associated steatotic liver disease (MASLD), previously referred to as nonalcoholic fatty liver disease (NAFLD), represents the most common chronic liver condition affecting children and adolescents globally. MASLD encompasses a wide spectrum of hepatic abnormalities, ranging from simple steatosis, characterized by fat accumulation in liver cells, to metabolic dysfunction-associated steatohepatitis (MASH), fibrosis, and potentially cirrhosis. The hallmark of MASLD is an excessive accumulation of hepatic fat in individuals without significant alcohol consumption or other identifiable liver diseases. Increasingly recognized as the hepatic manifestation of metabolic syndrome, MASLD is closely linked to obesity, insulin resistance, and various metabolic dysfunction components such as dyslipidemia and hypertension [1].

Recent epidemiological studies have shed light on the burden of MASLD among pediatric populations. A meta-analysis has estimated the prevalence of MASLD to be 7.6% in the general pediatric population and 34.2% among children attending obesity clinics [2]. Furthermore, MASLD prevalence appears to increase with age, starting as low as 0.7% in children aged 2 to 4 years and rising significantly to 17.3% in those aged 15 to 19 years [3]. Projections suggest that the incidence of MASLD will continue to rise, with a predicted prevalence of 30.7% by 2040 [4].

Despite extensive research efforts, the precise pathophysiological mechanisms underlying MASLD and its progression to MASH remain incompletely understood. Current evidence points to a “multiple-parallel hits” model, wherein genetic predispositions, environmental triggers, and metabolic imbalances interact to drive disease onset and progression. Key risk factors for pediatric MASLD include obesity, type 2 diabetes, dyslipidemia, and sedentary lifestyles. Additional predictors include elevated body mass index (BMI), increased waist circumference, and the consumption of diets high in calories, saturated fats, and added sugars [5, 6].

The cornerstone of MASLD management in children remains lifestyle modification, including dietary changes, weight reduction, and increased physical activity [7]. These interventions aim to reduce hepatic fat, improve insulin sensitivity, and mitigate systemic inflammation. However, achieving adherence to lifestyle changes can be particularly challenging in pediatric populations, often limiting their long-term efficacy. Despite their central role, current guidelines lack specific exercise prescriptions for MASLD management due to insufficient evidence regarding the effectiveness of various exercise regimens in this population [8].

Pharmacologic and non-pharmacologic therapies are increasingly being explored as adjuncts to lifestyle interventions. Among these, vitamin E has shown promise as an antioxidant, with studies reporting improvements in some histological parameters, such as hepatocellular ballooning scores. However, its impact on liver enzyme levels, such as alanine aminotransferase (ALT), showed insignificant results [9]. Other emerging treatments include vitamin D supplementation [10], insulin sensitizers like metformin [9], and N-acetylcysteine [11, 12]. Omega-3 fatty acids have also been evaluated for their role in improving lipid metabolism and reducing hepatic steatosis [12]. Orlistat, a lipase inhibitor that reduces dietary fat absorption, has demonstrated significant improvements in pediatric MASLD in randomized controlled trials (RCTs) [13]. Despite these promising therapeutic options, there remains no universally accepted pharmacologic treatment for pediatric MASLD [14]. This therapeutic gap highlights an urgent need for further research and the development of targeted interventions to prevent disease progression to MASH or cirrhosis.

The primary objective of this systematic review and network meta-analysis (NMA) is to comprehensively assess the efficacy and safety of current and emerging treatments for MASLD in children and adolescents. By synthesizing evidence exclusively from RCTs, this study aims to identify the most effective interventions for improving hepatic outcomes, including laboratory markers such as liver enzyme levels, lipid profiles, and metabolic parameters, as well as histological outcomes, such as reductions in hepatic steatosis and fibrosis. We believe these findings will help address critical gaps in the understanding and management of MASLD in pediatric populations.

## Methods

An in-depth investigation and NMA were conducted according to the Preferred Reporting Items for Systematic Reviews and Meta-Analyses for Network Meta-Analyses (PRISMANMA) guidelines [15]. The study protocol was registered (registration number: CRD42024583706) with the International Prospective Register of Systematic Reviews (PROSPERO).

###  Eligibility criteria

Included in the study were randomized controlled trials written in English that compared various drug therapies for treating pediatric MASLD in individuals meeting specific criteria: Patients: pediatric patients who met the approved international diagnostic criteria [14]. The diagnosis was achieved through medical imaging techniques such as ultrasonography or magnetic resonance imaging (MRI), or via liver biopsy, provided there were no other chronic liver diseases or secondary causes of liver steatosis. Intervention: studies comparing any regimens including pharmacological medications and supplements. Comparator: All possible comparisons among the included interventions were explored. Outcomes: The main outcomes of interest were NAFLD Activity Score (NAS), Nonalcoholic steatohepatitis (NASH) resolution, fibrosis score improvement, liver transaminases, and lipid profile. The minimum follow-up period was one month.

Exclusion criteria: Duplicate publications, literature reviews, letters to the editor, abstracts from conference proceedings, studies that assessed the acute effects of a single exercise session, and animal model research, studies lacking access to full text or raw data will be excluded, and studies reporting lifestyle intervention or probiotics alone.

### Search strategy

Five electronic databases were searched (PubMed, Embase, The Cochrane Library, Scopus, and Web of Science). The search strategy was built around the eligibility criteria. Search terms can be found in (Table S1) uploaded on 15 May 2024, and re-runed on 24 August 2024.

### Study selection and data extraction

Six reviewers (ME, MN, MK, SY, HY, AH) independently screened the selected studies from the electronic databases to assess their eligibility for inclusion via Rayyan software, and a duplicate check was done. Full-text screening was done by two reviewers (HY, SY). The reference lists of the included full-text articles and relevant systematic reviews were also manually screened (snowballing technique) to identify any additional eligible studies [16].

Data extraction was performed using a uniform data extraction sheet. All data conversions, including transformation of medians (Interquartile range/range) to means (standard deviation) and other reported formats (e.g., standard errors or confidence intervals to standard deviations), were performed using the Meta-Analysis Accelerator tool [17]. Extracted data included the following: Study characteristics, Baseline characteristics of the patients, Risk of Bias assessment and support for judgment, and Data of the studies’ outcome reliable for analysis, type and dose of nutritional supplementation. Six authors independently and in duplicate conducted the data extraction. Any conflicts were resolved through discussion and consensus.

### Quality assessment and risk of bias

The risk of bias for the included studies was assessed independently and in duplicate by (ME, MN, MK, SY, HY, AH) authors. It was performed using the risk-of-bias tool recommended by the Cochrane Collaboration for randomized trials (RoB2) [18].

### Statistical analysis

NMA was performed using R Studio (version 4.4.2) with the netmeta package to combine direct and indirect evidence [19]. The relative effect on multiple intervention comparisons was estimated using random-effects NMA. Mean differences (MDs) and 95% confidence intervals were used for continuous outcomes. Relative risks (RRs) and 95% confidence intervals were used for categorical outcomes. The surface under the cumulative ranking curves (SUCRA) value and cumulative ranking plots were used to calculate the ranking probabilities of each intervention. To further evaluate treatment hierarchies, we will generate rankograms to visualize the probability distributions of each intervention’s ranking across all possible positions. Heterogeneity in each pairwise comparison was assessed with the I ² statistic (a p-value >0.05 indicating absence of significant heterogeneity). Inconsistency is tested by the node splitting method. To assess the robustness of the findings, a sensitivity analysis was performed by excluding studies with a high risk of bias. This helped evaluate whether the overall results were influenced by methodological limitations in individual studies. We also did Bayesian network meta-regression to assess whether baseline body mass index (BMI) or study duration influenced treatment effects. Analyses were performed for the primary histological outcome (NAS) and for key biochemical outcomes (AST and ALT). All models were implemented using the MetaInsight online platform (University of Leicester) [20].

## Results

### Search results

**Fig. 1:**
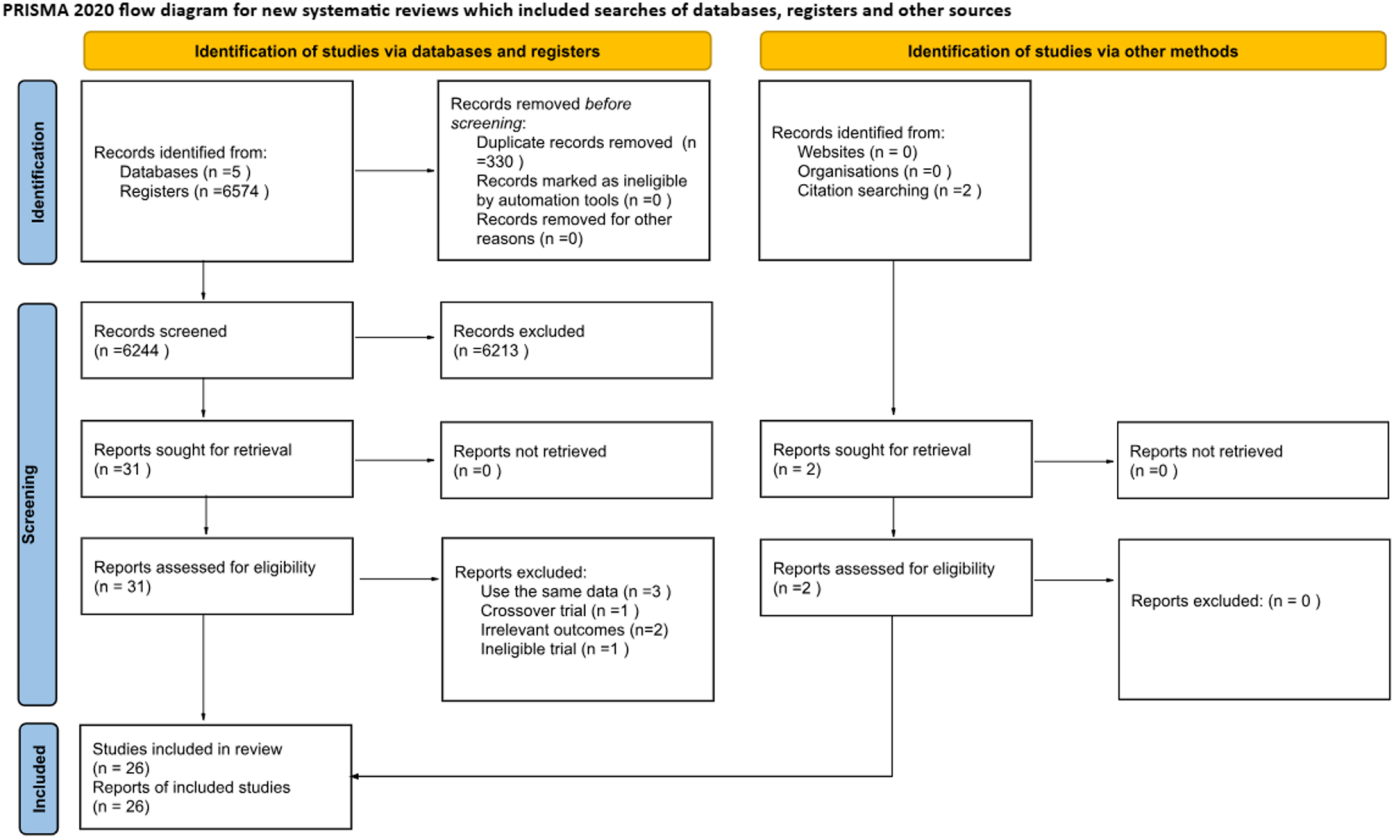
PRISMA flow diagram of included and excluded network meta-analyses. PRISMA = Preferred Reporting Items for Systematic Reviews and Meta-Analyses

Figure 1 illustrates the procedure for identifying eligible trials. We initially identified 6574 studies, after screening studies by title and abstract and removing duplicates, we identified 26 eligible studies [9–13, 21–41].

### Trials characteristics

The baseline characteristics of each trial are represented in (Table S2), we included 26 RCTs involving 1503 pediatric patients. (Table S3) displays the characteristics of the included trials.

### Quality assessment

**Fig. 2:**
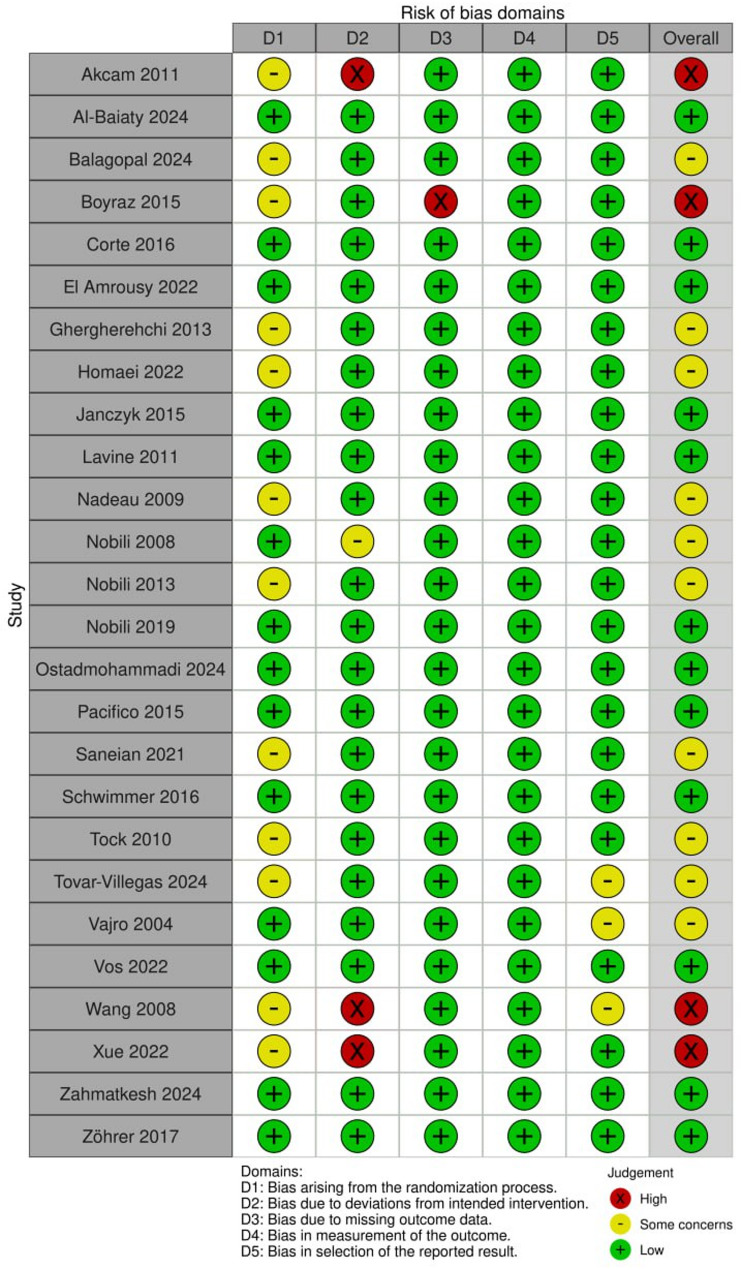
Quality assessment of randomized controlled trials (RCTs) using the Cochrane risk of bias 2 tool

Detailed results regarding risk of bias assessment are available in (Fig. 2, S1). In general, 12 trials were found to be low, 10 trials presented some concerns, four trials were rated as having high risk of bias [12], didn’t report the reason for the missing outcome data, [21, 39, 40] failed to implement the intervention equally between trial arms.

### Biopsy efficacy outcomes

#### NAS

**Fig. 3:**
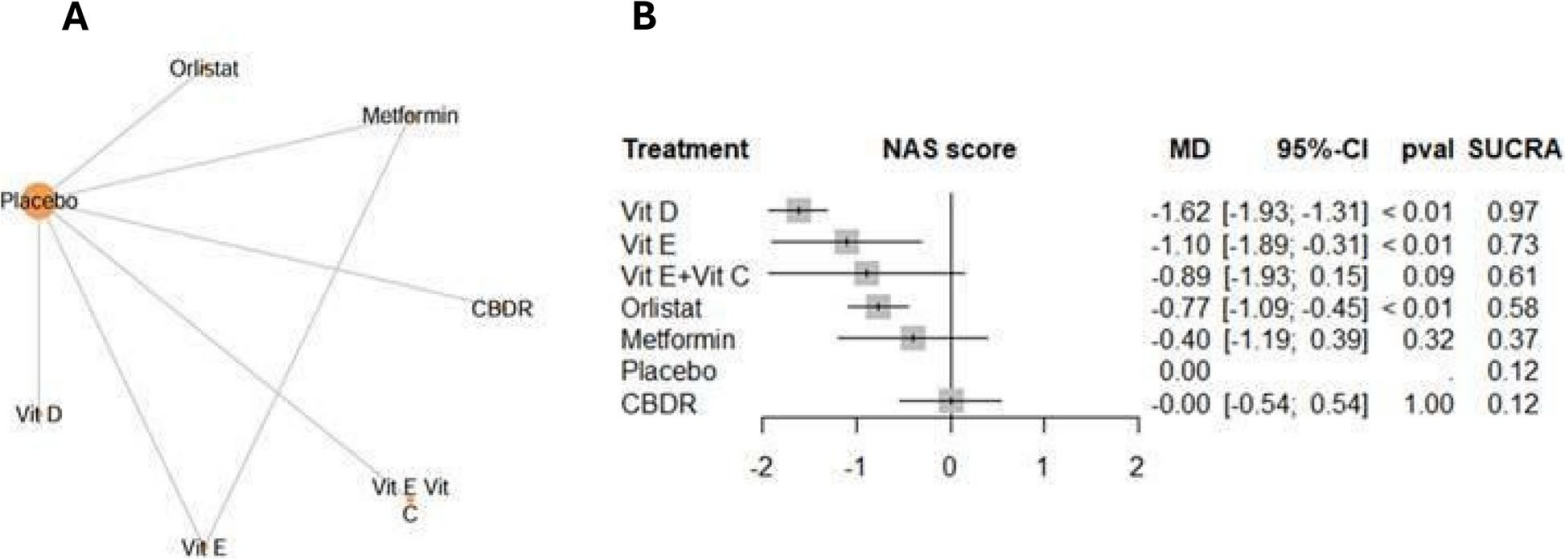
**A**, Network geometry of NAS for all included studies, by drug treatments. **B**, Forest plots for network meta-analysis of NAS

Six trials with 522 patients reported on the NAS score. (Fig. 3) represents the network geometry and the NMA results compared to placebo and (figure S2 and Table 1) display the ranking of the interventions. Vitamin D displayed the most promising results by achieving the highest ranking among the interventions: SUCRA (97%). Besides, Vitamin D displayed significant improvements in NAS score compared to Orlistat, Metformin and cysteamine bitartrate delayed release (CBDR). Vitamin E and Orlistat displayed significant improvements compared to CBDR.

*NAS = NAFLD Activity Score*,* CBDR = cysteamine bitartrate delayed release; Vit. = vitamin.*

**Table 1:**
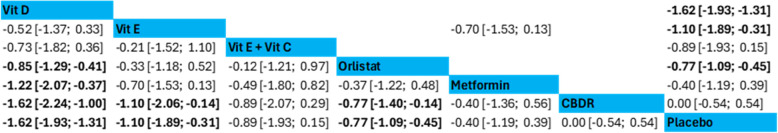
League table for pairwise meta-analysis (right upper part) and NMA (left lower part) effect estimates: NAFLD activity Score. CBDR = cysteamine bitartrate delayed release; Vit = vitamin

#### NASH resolution events

Three trials with 247 patients reported on NASH resolution events. (Figures S3, S4) represent the network geometry and the NMA results compared to placebo and (figure S5 and table S4) display the ranking of the interventions. Vitamin E displayed the most promising results by achieving the highest ranking among the interventions: SUCRA (83%). Vitamin E solely displayed significant improvement compared to placebo: (RR: 2.14, 95%CI 1.19 to 3.84). However, there were no significant differences between vitamin E and other interventions.

#### Histological improvement in Fibrosis, lobular Inflammation, Steatosis, and ballooning (Events and Scores)

Across four trials with 469 patients, improvements were assessed in fibrosis, lobular inflammation, steatosis, and ballooning. For fibrosis, no significant differences were observed between the interventions and placebo (Figures S6–S10; Tables S5–S6). For lobular inflammation, vitamin D and CBDR displayed the highest rankings in improvement events (SUCRA: 98% and 75%, respectively) and score (SUCRA: 84% and 80%, respectively). The incidence of lobular inflammation improvement on vitamin D was significantly higher compared to vitamin E, metformin, and placebo (Figures S11–S15; Tables S7–S8). For steatosis, vitamin D ranked highest for improvement events (SUCRA: 99%), with a significantly higher incidence compared to vitamin E, metformin, vitamin E plus vitamin C, placebo, and CBDR. Vitamin E showed a significantly higher incidence of improvement compared to CBDR (RR: 1.84, 95%CI 1.01 to 3.35). For mean change in steatosis score, orlistat showed a significant reduction (MD: −0.22, 95%CI −0.38 to −0.06, SUCRA: 56%), while no other intervention differed significantly from placebo (Figures S16–S21; Tables S9–S10). For ballooning, vitamin E and metformin both showed significantly higher incidences of improvement compared to placebo (RR: 2.07, 95%CI 1.10 to 3.89, SUCRA: 73%) and had the best reductions in score (vitamin E: MD: −0.60, 95%CI −0.94 to −0.26, SUCRA: 92%; metformin: MD: −0.40, 95%CI −0.78 to −0.02, SUCRA: 70%). Vitamin D ranked highest for ballooning (SUCRA: 88%), though there were no significant differences between vitamin D, CBDR, vitamin E plus vitamin C, and placebo (Figures S22–S27; Tables S11–S12).

#### Portal inflammation improvement events and score

Two trials comprising 222 patients reported on the number of patients displaying improvements in the portal inflammation score. (Figures S28-S30) represent the network geometry and the NMA results compared to placebo and **(**Figure S31-S32 and Table S13-S14) display the ranking of the interventions. There were no significant differences between the interventions investigated and the placebo.

### Laboratory efficacy outcomes

#### Aspartate transaminase (AST)

Twenty trials, including 1267 patients, reported data regarding AST levels. (Figures S33-S34) represent the network geometry and the NMA results compared to placebo and (figure S35 and table S15) display the ranking of the interventions. CBDR, followed by vitamin E + hydroxytyrosol (HXT), followed by vitamin D displayed the highest AST reductions compared to placebo: (MD: −27.00 units per liter (U/L), 95%CI −41.25 to −12.75, SUCRA: 94%), (MD: −17.20 (U/L), 95%CI −30.13 to −4.27, SUCRA: 81%), and (MD: −14.00(U/L), 95%CI −18.20 to −9.80, SUCRA: 76%), respectively. Besides, the before mentioned three interventions displayed significant reductions in AST levels compared to orlistat, vitamin E, zinc, metformin, losartan and L-carnitine as shown in (table S15). The above-mentioned results are of the sensitivity analysis after removing [12, 39, 40]. Results before sensitivity analysis are displayed in (figure S36-S38 and table S16).

#### Alanine transaminase (ALT)

**Fig. 4:**
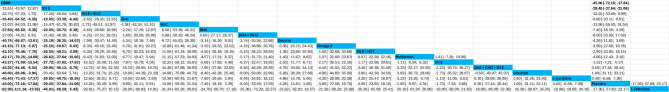
**A**, Network geometry of ALT for all included studies, by drug treatments. **B**, Forest plots for network meta-analysis of ALT. ALT = Alanine transaminase, CBDR = cysteamine bitartrate delayed release; Vit = vitamin; NAC = N-Acetylcysteine; DHA = Docosahexaenoic acid; HXT = Hydroxytyrosol; CHO = Choline

Twenty-two trials comprising 1388 patients reported data regarding ALT levels. (Fig. 4) represent the network geometry and the NMA results compared to placebo and **(**figure S39 and Table 2) display the ranking of the interventions. Compared to placebo, CBDR and Vitamin D had the best and the only significant reductions in ALT levels compared to placebo: (MD: −45.00 (U/L), 95%CI −72.16 to −17.84, SUCRA: 98%) and (MD: −29.45(U/L), 95%CI −37.84 to −21.06, SUCRA: 92%), respectively. Besides, both CBDR and vitamin D showed significant ALT reductions compared to most of the investigated interventions, as shown in (Table 2). The above-mentioned results are of the sensitivity analysis after removing [12, 39, 40]. Results before sensitivity analysis are displayed in (figure S40-42 and table S17).

**Table 2:**
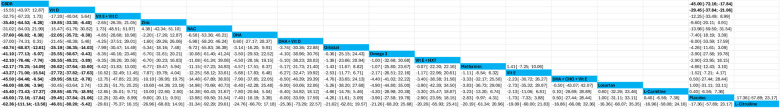
League table for pairwise meta-analysis (right upper part) and NMA (left lower part) effect estimates: ALT (results after sensitivity analysis)*.*ALT = Alanine transaminase; CBDR = cysteamine bitartrate delayed release; Vit = vitamin; NAC = N-Acetylcysteine; DHA = Docosahexaenoic acid; HXT = Hydroxytyrosol; CHO = Choline

#### Alkaline phosphatase (ALP)

Three trials comprising 369 patients reported data regarding ALP levels. (Figures S43-S44) represent the network geometry and the NMA results compared to placebo and **(**figure S45 and table S18) display the ranking of the interventions. Compared to placebo, Losartan had the best and the only significant reductions in ALT levels compared to placebo: (MD: −23.40(U/L), 95%CI −42.93 to −3.87, SUCRA: 82%). There were no significant differences between the other interventions (metformin, CBDR, vitamin E) and placebo as shown in (table S18).

#### Lipid profile (total cholesterol, triglycerides, low-density lipoprotein cholesterol (LDL), and High-density lipoprotein cholesterol (HDL))

Seventeen trials including 1,197 patients reported data related to total cholesterol levels. (Figures S46-S47) represent the network geometry and the NMA results compared to placebo and **(**figure S48 and table S19) display the ranking of the interventions. Vitamin D, followed by vitamin E + vitamin C, followed by orlistat displayed the highest and the only significant total cholesterol reductions compared to placebo: (MD: −35.5 milligrams per deciliter mg/dL, 95%CI −54.04 to −16.96, SUCRA: 96%), (MD: −20.50 mg/dL, 95%CI −37.64 to −3.36, SUCRA: 85%), and (MD: −9.28 mg/dL, 95%CI −14.59 to −3.97, SUCRA: 70%), respectively. Moreover, vitamin D displayed significant reductions compared to all interventions but vitamin E + vitamin C and L-citrulline as shown in table (S19). Besides, vitamin E + vitamin C and orlistat displayed significantly superior total cholesterol reductions compared to vitamin E: (MD: −20.31 mg/dL, 95%CI −37.46 to −3.15) and (MD: −9.09 mg/dL, 95%CI −14.46 to −3.72), respectively. Additionally, vitamin E + vitamin C showed significantly favorable total cholesterol reductions compared to docosahexaenoic acid (DHA) (MD: −28.00 mg/dL, 95%CI −55.65 to −0.35). The above-mentioned results are of the sensitivity analysis after removing [12, 39, 40]. Results before sensitivity analysis are displayed in (figure S49-S51 and table S20).

As for triglycerides, eighteen trials reported relevant data, incorporating 1,257 patients. (Figures S52-S53) represent the network geometry and the NMA results compared to placebo and (figure S54 and table S21) display the ranking of the interventions. Compared to placebo, only DHA + vitamin D, vitamin D alone, and orlistat displayed significant reductions in triglyceride levels: (MD: −74.59 mg/dL, 95%CI −106.58 to −42.60, SUCRA: 97%), (MD: −33.45 mg/dL, 95%CI −42.16 to −24.74, SUCRA: 83%), and (MD: −4.40 mg/dL, 95%CI −7.38 to −1.42, SUCRA: 37%), respectively. DHA + vitamin D had the best ranking among all treatment arms and significantly reduced triglyceride levels compared to all interventions, as shown in (table S21). Besides, vitamin D alone ranked second-best and significantly reduced triglycerides compared to half of the investigated interventions. Remarkably, orlistat didn’t rank high among the interventions (SUCRA: 37%), but it also significantly reduced triglycerides compared to vitamin E (MD: −4.51 mg/dL, 95%CI −7.54 to −1.48). The above-mentioned results are of the sensitivity analysis after removing [12, 39, 40]. Results before sensitivity analysis are displayed in (figure S55-S57 and table S22).

Regarding LDL levels, fourteen trials reported relevant data, including 1,064 patients. (Figures S58-S59) represent the network geometry and the NMA results compared to placebo and **(**figure S60 and table S23) display the ranking of the interventions. Vitamin D, CBDR, and orlistat solely displayed significant reductions in LDL levels compared to placebo: (MD: −13.50 mg/dL, 95%CI −23.17 to −3.83, SUCRA: 86%), (MD: −7.00 mg/dL, 95%CI −13.20 to −0.80, SUCRA: 69%), and (MD: −5.66 mg/dL, 95%CI −9.78 to −1.54, SUCRA: 65%), respectively. In addition to vitamin D scoring the highest rank in LDL reduction, vitamin D displayed significant reductions compared to vitamin E, metformin and omega 3: (MD: −14.81 mg/dL, 95%CI −26.47 to −3.16)., (MD: −14.97 mg/dL, 95%CI −26.63 to −3.31), and (MD: −25.30 mg/dL, 95%CI −49.95 to −0.65), respectively as shown in (table S23). The above-mentioned results are of the sensitivity analysis after removing [12]. Results before sensitivity analysis are displayed in (figure S61-S63 and table S24).

Concerning HDL levels, fourteen trials reported relevant data, including 1,082 patients. (Figures S64-S65) represent the network geometry and the NMA results compared to placebo and **(**figure S66 and table S25) display the ranking of the interventions. Vitamin D alone, Vitamin D + DHA, and orlistat solely displayed significant increments in HDL levels compared to placebo: (MD: 8.85 mg/dL, 95%CI 6.47 to 11.23, SUCRA: 96%), (MD: 8.31 mg/dL, 95%CI 1.89 to 14.73, SUCRA: 92%), and (MD: 1.65 mg/dL, 95%CI 0.47 to 2.83, SUCRA: 57%), respectively. Vitamin D alone followed by vitamin D + DHA achieved highest ranking in increasing HDL in addition to significantly increasing HDL compared to most of the other interventions as shown in (table S25). Although zinc and metformin didn’t display significant increments in HDL compared to placebo, they displayed significant increments in HDL compared to vitamin E: (MD: 4.51 mg/dL, 95%CI 0.05 to 8.97), and (MD: 2.66 mg/dL, 95%CI 0.13 to 5.19), respectively as shown in (table S25). The above-mentioned results are of the sensitivity analysis after removing [12]. Results before sensitivity analysis are displayed in (figure S67-S69 and table S26).

#### Fasting blood glucose, fasting blood insulin (FBI), homeostatic model assessment of insulin resistance (HOMA-IR)

Fifteen trials compromising 997 patients reported data regarding fasting blood glucose levels. (Figures S70-S71) represents the network geometry and the NMA results compared to placebo and **(**figure S72 and table S27) display the ranking of the interventions. Orlistat solely reduced fasting blood glucose compared to placebo: (MD: −4.23 mg/dL, 95%CI −6.75 to −1.71, SUCRA: 83%). However, there were no significant differences between orlistat and other interventions. Interestingly, vitamin D resulted in significantly higher fasting blood glucose levels compared to placebo (MD: 8.50 mg/dL, 95%CI 5.77 to 11.23, SUCRA: 3%) and almost all other interventions as shown in (table S27). The above-mentioned results are of the sensitivity analysis after removing [12, 21, 39, 40]. Results before sensitivity analysis are displayed in (figure S73-S75 and table S28).

As for FBI, fourteen trials reported relevant data, including 968 patients. (Figures S76-S77) represent the network geometry and the NMA results compared to placebo and (figure S78 and table S29) display the ranking of the interventions. Compared to placebo vitamin E + HXT, followed by vitamin D, followed by metformin displayed the highest ranking in reducing FBI among all interventions. Both vitamin E + HXT and vitamin D displayed significant FBI reductions compared to orlistat, vitamin E + vitamin C and losartan, with vitamin D showing additional significantly superior FBI reductions to omega 3. Metformin and DHA showed significant FBI reductions compared to vitamin E + vitamin C and losartan. The above-mentioned results are of the sensitivity analysis after removing [12, 21, 39, 40]. Results before sensitivity analysis are displayed in (figure S79-S81 and table S30).

Regarding HOMA-IR, seventeen trials reported relevant data, including 1,150 patients. (Figures S82-S83) represent the network geometry and the NMA results compared to placebo and **(**figure S84 and table S31) display the ranking of the interventions. Compared to placebo, vitamin D, followed by N-acetylcysteine (NAC) displayed the highest ranking in reducing HOMA-IR among all interventions. The above-mentioned results are of the sensitivity analysis after removing [12, 21, 39, 40]. Results before sensitivity analysis are displayed in (figure S85-S87 and table S32).

### Anthropometric measures

#### BMI

**Fig. 5:**
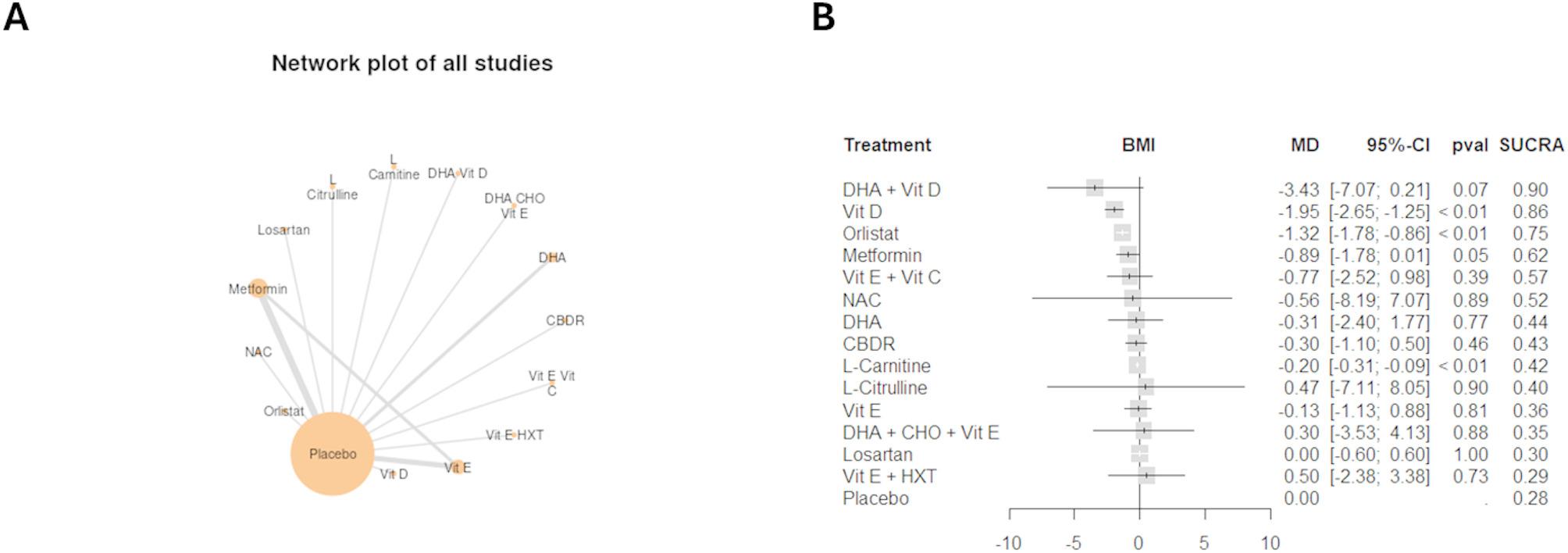
**A**, Network geometry of BMI for all included studies, by drug treatments. **B**, Forest plots for network meta-analysis of BMI. BMI = Body mass index, CBDR = cysteamine bitartrate delayed release; Vit = vitamin; NAC = N-Acetylcysteine; DHA = Docosahexaenoic acid; HXT = Hydroxytyrosol; CHO = Choline

Eighteen trials compromising 1,215 patients reported data regarding BMI. (Fig. 5) represents the network geometry and the NMA results compared to placebo and **(**figure S88 and Table 3) display the ranking of the interventions. Compared to placebo, only vitamin D, orlistat and L-citrulline significantly reduced BMI: (MD: −1.95 kg/m^2, 95%CI −2.65 to −1.25, SUCRA: 86%), (MD: −1.32 kg/m^2, 95%CI −1.78 to −0.86, SUCRA: 75%), and (MD: −0.20 kg/m^2, 95%CI −0.31 to −0.09, SUCRA: 42%), respectively. Moreover, vitamin D and orlistat significantly reduced BMI compared to CBDR, L-carnitine, vitamin E and losartan as shown in **(**Table 3). The above-mentioned results are of the sensitivity analysis after removing [12, 39]. Results before sensitivity analysis are displayed in (figure S89-S91 and table S33).

**Table 3:**
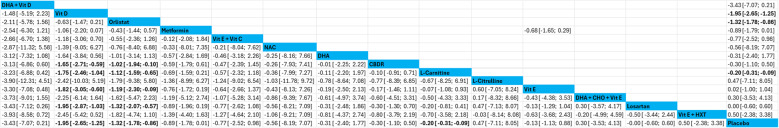
League table for pairwise meta-analysis (right upper part) and NMA (left lower part) effect estimates: BMI(results after sensitivity analysis). BMI = Body mass index; CBDR = cysteamine bitartrate delayed release; Vit = vitamin; NAC = N-Acetylcysteine; DHA = Docosahexaenoic acid; HXT = Hydroxytyrosol; CHO = Choline

#### Body mass index standard deviation score (BMI-SDS)

Eighteen trials compromising 619 patients reported data regarding BMI-SDS. (Figures S92-S93) represent the network geometry and the NMA results compared to placebo and **(**figure S94 and table S34) display the ranking of the interventions. Compared to placebo, only orlistat and CDBR significantly reduced BMI-SDS: (MD: −0.45, 95%CI −0.62 to −0.28, SUCRA: 86%), and (MD: −0.10, 95%CI −0.18 to −0.02, SUCRA: 54%), respectively. Moreover, orlistat significantly reduced BMI-SDS compared to CBDR, metformin, vitamin E, L-carnitine, and DHA as shown in (table S34). The above-mentioned results are of the sensitivity analysis after removing [12, 21, 39, 40]. Results before sensitivity analysis are displayed in (figure S95-S97 and table S35).

#### Waist circumference

Eighteen trials compromising 780 patients reported data regarding BMI. (Figures S98-S99) represent the network geometry and the NMA results compared to placebo and (figure S100 and table S36) display the ranking of the interventions. Orlistat and L-carnitine solely displayed significant waist circumference reductions compared to placebo: (MD: −3.27 cm, 95%CI −4.71 to −1.83, SUCRA: 88%), and (MD: −0.32 cm, 95%CI −0.59 to −0.05, SUCRA: 56%), respectively. Orlistat displayed the highest ranking in reducing waist circumference among the investigated interventions and showed significantly lower reductions in waist circumference compared to L-carnitine, CBDR, losartan, and DHA plus CHO plus vitamin E: (MD: −2.95 cm, 95%CI −4.41 to −1.49), (MD: −3.47 cm, 95%CI −6.28 to −0.66), (MD: −5.77 cm, 95%CI −8.95 to −2.59), and (MD: −8.27 cm, 95%CI −16.15 to −0.39), respectively as shown in (table S36).

### Metaregression analysis

Meta-regression showed no significant association between BMI or study duration and treatment effects across outcomes. For NAS, regression coefficients were not significant for BMI (B = − 1.36, 95% CrI − 14.81 to 5.13) or duration (B = − 0.01, 95% CrI − 5.63 to 5.88). Similarly, for AST no significant associations were detected for BMI (B = − 2.26, 95% CrI − 13.23 to 10.29) or duration (B = − 1.66, 95% CrI − 12.14 to 9.45). ALT analyses also indicated no significant effect of BMI (B = − 3.55, 95% CrI − 22.56 to 12.39) or duration (B = 5.53, 95% CrI − 10.27 to 18.70). Full model outputs are presented in (tables S37-S52). In addition, there was no significant regression coefficient between baseline AST and change in NAS (B = 1.28, 95% CrI − 4.50 to 18.14) or baseline ALT and change in NAS(B = 0.75, 95% CrI − 4.20 to 8.97).

## Discussion

The current SR and NMA comprehensively assess the efficacy of pharmacotherapeutics in pediatric MASLD. Vitamin D 2000 IU appeared to be the most promising intervention by displaying superior improvements in histological and laboratory outcomes. Vitamin D displayed a 1.62-point reduction in NAS alongside a superior ranking among the interventions in the number of patients achieving improvement in steatosis and lobular inflammation (SUCRA: 99% and 98%, respectively). This proves a clinically significant histological improvement in the MASLD pediatric population. Moreover, clinical data indicate a correlation between hypovitaminosis D and MASLD, suggesting that vitamin D supplementation may serve as a potential therapeutic option for MASLD in children [42]. Interestingly, these results contradict the findings displayed by some trials that investigated vitamin D in MASLD [43–45]. For example, Kitson MASH pilot study [46], which involved patients with MASH undergoing liver biopsy both before and after a six-month course of 25,000 IU of cholecalciferol weekly supplementation, revealed no impact on liver outcomes such as intra-hepatocyte fat accumulation, fibrosis, or local inflammation. This disparity might be attributed to baseline vitamin D levels of patients in those trials, patient characteristics, vitamin D dosing, and follow-up duration. However, it is important to acknowledge that the trials participating in this meta-analysis are conducted on the pediatric population, which might raise questions regarding the relationship between age and response to vitamin D. Notably, our metaregression analyses revealed no significant effect of follow-up duration or baseline BMI, which is believed to influence treatment response across trials, on NAS or lab values (ALT and AST) (tables S49-S52).

Another prominent intervention in our NMA was vitamin E. Patients treated with vitamin E were the most likely to achieve NASH resolution compared to other interventions. Moreover, vitamin E had promising enhancements in laboratory outcomes compared to other interventions in this NMA. The effects of vitamin E align with one of the most guiding trials on vitamin E supplementation in adults with MASLD [47]. In the trial by Vilar-Gomez et al., patients were followed up for more than 5 years, and vitamin E has been shown to have palpable improvements in the clinical outcomes of MASLD patients regardless of their diabetic status. Vitamin E improvements in blood apolipoprotein-A1, AST levels, reduced DNA damage, and low expression of pro-inflammatory cytokines were all predictors of its efficacy in MASLD [22].

Notably, CBDR resulted in superior enhancements in laboratory outcomes compared to placebo and other pharmacotherapeutics in this NMA. However, CBDR barely resulted in a significant improvement in histological parameters. This raises concerns about whether laboratory improvements, represented as reductions in AST and ALT mainly, can be translated to improvements in histological parameters. Moreover, our metaregression analysis revealed that the baseline AST and ALT levels didn’t alter the reductions observed in NAS score in our meta-analysis (tables S49-S52).

It is important to appreciate that although pharmacotherapy evolves as a promising solution for pediatric MASLD, lifestyle modifications remain the cornerstone of management of this disease. Dietary approaches, regardless of their micro- and macronutrient compositions, tend to improve MASLD mainly through weight loss [48]. However, most of the dietary approaches are faced with challenges of poor long-term adherence, variability in individual metabolic response, and socioeconomic barriers. Besides, the lack of robust data on the details and beneficial effects of exercise programs in pediatric MASLD makes lifestyle modifications even more challenging [49]. Some evidence supports that pharmacotherapy with insulin sensitizers like metformin plus lifestyle modifications is superior to lifestyle changes alone [31]. However, these trials lack statistical power and methodological robustness. Besides, many trials adopting lifestyle modifications alongside pharmacological interventions vary in their lifestyle recommendations and patient surveillance, which deters their comparison and critique. Therefore, the true potential of pharmacotherapy alongside lifestyle modifications in MASLD, though promising, is yet to be known.

Interestingly, Resmetirom (Rezdiffra) (March 2024) has been approved by the FDA for treating non-cirrhotic NASH with fibrosis (F2-F3) in adults, based on histologic improvement, with confirmatory outcomes to be determined. In August 2025, semaglutide (Wegovy) was also approved by the FDA for MASH with liver scarring, showcasing the rising foothold of metabolic therapy in adults[50, 51]. On the other hand, there is no FDA-approved pharmacotherapy for any spectrum of pediatric MASLD. The limited therapeutic pipeline in children reflects wider problems in conducting pediatric hepatology research: diagnosis relies still on liver biopsy, which also raises ethical concerns and needs parental consent; non-invasive biomarkers are less validated for use in children; and practical issues such as recruitment and retention, traveling to specialized centers, caregiver burden, family privacy, and having other family members participate in follow-up make trial conduct difficult. All these challenges have so far limited both the number and the size of pediatric trials, with the resulting evidence base being much weaker than in adults [52].

This NMA is among the first to comprehensively evaluate pharmacotherapy in pediatric MASLD, bringing in antioxidants, supplements, and antidiabetics into a single modeling framework that allows for indirect comparisons between drug classes. Sensitivity analyses by excluding studies at high risk of bias were performed to ensure robustness, and meta-regression was done to assess the effects of baseline parameters and follow-up duration on histologic and biochemical outcomes.

Nevertheless, some limitations should be kept in mind. Data extraction was limited to what the original publications reported, with only six trials analyzed by intention-to-treat analysis (ITT), while the rest were per-protocol, so harmonization of analytic strategies was prevented. Further heterogeneity came from MASLD diagnosis not being standardized across studies. Dose stratification (e.g., vitamin E) was not possible; to preserve network connectivity, it was necessary to group by intervention, which was also adopted by previous NMAs on the topic (chen et al., 2022 [53]). Trials that co-administered lifestyle interventions with pharmacotherapy were excluded because, as mentioned earlier, lifestyle modifications differ from one trial to another and would have heavily affected transitivity. Lastly, variability in age, baseline severity, and BMI across trials may have influenced results, although meta-regression suggested these factors did not significantly alter treatment effects.

Our future recommendations stem from the limitations of this NMA and the current literature on pediatric MASLD pharmacotherapy. Future studies with larger cohorts, strict MASLD diagnosis criteria (preferably using biopsy), and standardised lifestyle modifications are highly recommended. These recommendations can seal the gap in the potential of pediatric pharmacotherapy alongside lifestyle modifications. Moreover, studies assessing the real-world effectiveness of pharmacotherapy by considering socioeconomic factors and cost-effectiveness will reflect the practicality of pharmacotherapy in pediatric MASLD.

## Conclusions

The findings of our NMA show that pharmacotherapy is a promising strategy for pediatric MASLD. 2000 IU Vitamin D was effective in managing MASLD. Vitamin E is notably effective in achieving NASH resolution and enhancing laboratory parameters. While CBDR best reduces liver transaminases, it has minimal effect on histological outcomes. These findings underscore the great potential of vitamin D and vitamin E in managing pediatric MASLD. Future RCTs with robust MASLD diagnosis criteria that can incorporate lifestyle modifications alongside pharmacotherapy are highly recommended.

## Supplementary Information

Supplementary Material 1.

## Data Availability

Data is provided within the manuscript or supplementary information files.

## Code availability

The code used for data analysis in this study was implemented using R software. The R code utilized for data analysis in this study is available upon reasonable request from the corresponding author.
