# Supplementary material for: Efficacy of pharmacotherapies on pediatric patients with metabolic dysfunction-associated steatotic liver disease: a systematic review and network meta-analysis
Source: BMC Gastroenterol. 2025 Dec 8;25:853. doi: 10.1186/s12876-025-04393-x (PMC12683787; doi:10.1186/s12876-025-04393-x)
Supplement: Supplementary file 1 — Supplementary Material 1. [file 12876_2025_4393_MOESM1_ESM.docx]

Tables

Table S1 Search strategy

| Database (Number of studies) | Search strategy |
| --- | --- |
| PUBMED (796) | **("non alcoholic fatty liver disease"[MeSH Terms] OR "NASH" [All Fields] OR "NAFLD"[All Fields] OR (non alcoholic fatty liver disease*) OR "nonalcoholic fatty liver disease"[Title/Abstract] OR "non-alcoholic fatty liver disease*"[Title/Abstract] OR "NAFLD"[Title/Abstract] OR "NASH"[Title/Abstract] OR "nonalcoholic steatohepatitis"[Title/Abstract] OR "non-alcoholic steatohepatitis"[Title/Abstract] OR "non alcoholic steatohepatitis"[Title/Abstract] OR "non alcoholic fatty liver"[Title/Abstract]OR "non-alcoholic fatty liver"[Title/Abstract] OR Steatohepatosis[Title/Abstract] OR "Hepatic steatosis"[Title/Abstract] OR "Metabolic dysfunction-associated fatty liver disease"[Title/Abstract] OR MASH[Title/Abstract] OR "Fatty liver disease"[Title/Abstract] OR "metabolic dysfunction-associated steatotic liver disease"[Title/Abstract] OR MASLD[Title/Abstract]) AND ("Randomised controlled trial" OR "Clinical trial" OR RCT OR Randomised controlled trial OR "randomized" OR "TRIAL" OR Randomized controlled trial) AND ("child*" OR "pediatric" OR "paediatric" OR "adolescen*" OR "infant"[MeSH Terms] OR "child"[MeSH Terms] OR "adolescent"[MeSH Terms])** |
| Embase (440) | (non alcoholic fatty liver disease:ti,ab,kw OR NASH:ti,ab,kw OR NAFLD:ti,ab,kw OR nonalcoholic fatty liver disease:ti,ab,kw OR non-alcoholic fatty liver disease:ti,ab,kw OR nonalcoholic steatohepatitis:ti,ab,kw OR non-alcoholic steatohepatitis:ti,ab,kw OR non alcoholic fatty liver:ti,ab,kw OR non-alcoholic fatty liver:ti,ab,kw OR Steatohepatosis:ti,ab,kw OR Hepatic steatosis:ti,ab,kw OR Metabolic dysfunction-associated fatty liver disease:ti,ab,kw OR MASH:ti,ab,kw OR Fatty liver disease:ti,ab,kw OR metabolic dysfunction-associated steatotic liver disease:ti,ab,kw OR MASLD:ti,ab,kw) AND (Randomised controlled trial:ti,ab,kw OR Clinical trial:ti,ab,kw OR RCT:ti,ab,kw OR randomized:ti,ab,kw OR TRIAL:ti,ab,kw) |
| WEB OF SCIENCE (757) | ALL=(("non alcoholic fatty liver disease" OR "NASH" OR "NAFLD" OR (non alcoholic fatty liver disease*) OR "nonalcoholic fatty liver disease" OR "non-alcoholic fatty liver disease*" OR "nonalcoholic steatohepatitis" OR "non-alcoholic steatohepatitis" OR "non alcoholic fatty liver" OR "non-alcoholic fatty liver" OR steatohepatisis OR "Hepatic steatosis" OR "Metabolic dysfunction-associated fatty liver disease" OR MASH OR "Fatty liver disease" OR "metabolic dysfunction-associated steatotic liver disease" OR MASLD) AND ("Randomised controlled trial" OR "Clinical trial" OR RCT OR "randomized" OR "TRIAL" ) AND ("child*" OR "pediatric" OR "paediatric" OR "adolescen*" OR "infant")) |
| COCHRANE (641) | ((non alcoholic fatty liver disease) OR (NASH) OR (NAFLD) OR (non alcoholic fatty liver disease*) OR (nonalcoholic fatty liver disease) OR (non-alcoholic fatty liver disease*) OR (nonalcoholic steatohepatitis) OR (non-alcoholic steatohepatitis) OR (non alcoholic fatty liver) OR (non-alcoholic fatty liver) OR Steatohepatosis OR (Hepatic steatosis) OR (Metabolic dysfunction-associated fatty liver disease) OR MASH OR (Fatty liver disease) OR (metabolic dysfunction-associated steatotic liver disease) OR MASLD) in Title Abstract Keyword AND ((Randomised controlled trial) OR (Clinical trial) OR RCT OR (randomized) OR (TRIAL) ) in Title Abstract Keyword AND ((child*) OR (pediatric) OR (paediatric) OR (adolescen*) OR (infant)) in Title Abstract Keyword - (Word variations have been searched) |
| Scopus (3940) | TITLE-ABS-KEY ( "non alcoholic fatty liver disease" OR "NASH" OR "NAFLD" OR "nonalcoholic fatty liver disease" OR "non-alcoholic fatty liver disease" OR "nonalcoholic steatohepatitis" OR "non-alcoholic steatohepatitis" OR "non alcoholic fatty liver" OR "non-alcoholic fatty liver" OR steatohepatosis OR "Hepatic steatosis" OR "Metabolic dysfunction-associated fatty liver disease" OR mash OR "Fatty liver disease" OR "metabolic dysfunction-associated steatotic liver disease" OR masld ) AND ( "Randomised controlled trial" OR "Clinical trial" OR rct OR "randomized" OR "TRIAL" ) AND ( "child" OR "children" OR "pediatric" OR "paediatric" OR "adolescent" OR "adolescents" OR "adolescence" OR "infant" ) AND ( LIMIT-TO ( SUBJAREA , "MEDI" ) OR LIMIT-TO ( SUBJAREA , "PHAR" ) ) AND ( LIMIT-TO ( DOCTYPE , "ar" ) ) AND ( LIMIT-TO ( LANGUAGE , "English" ) ) AND ( LIMIT-TO ( EXACTKEYWORD , "Human" ) ) |

Table S2, Baseline characteristics of the included studies.

| 1. Study ID | Study Drug | Age Mean (SD) | Gender, Male N (%) | BMI Mean (SD) | ALT, U/I Mean (SD) | NAS Mean (SD) |
| --- | --- | --- | --- | --- | --- | --- |
|  |  |  |  |  |  |  |
| Akcam 2011 | Metformin | 12 (2.9) | 32 (48) | 29 (5.5) | 25.4 (12.2) | N/A |
|  | Vit E | 12.6 (2.3) |  | 27.7 (3.2) | 27.8 (11.1) |  |
|  | Untreated | 11.3 (2.6) |  | 26.8 (4) | 21.5 (18.2) |  |
| Al-Baiaty 2024 | Vit E | 11.8 (2.1) | 12.14 (2.34) | N/A | 29 (15) | N/A |
|  | Placebo | 12.5 (2.6) |  |  | 30.4 (10.7) |  |
| Balagopal 2024 | NAC | 13.73 (3.11) | 7 (54) | 31.86 (4.64) | 90.13 (38.13) | 2 to 3^b^ |
|  | Placebo | 14.24 (2.76) |  | 32.18 (5.04) | 84.75 (26.87) | 2 to 3^b^ |
| Boyraz 2015 | Omega-3 | 13.8(3.8) | 73 (53) | 29.7 (4.8) | 58.2 (17.7) | N/A |
|  | Placebo | 13.3 (3.5) |  | 27.2 (3.3) | 56 (18.8) |  |
| Corte 2016 | DHA + Vit D | 12.3 (2.07) | N/A | 28.42 (4.08) | 40.25 (24.59) | 4.95 (0.82) |
|  | Placebo | 13.20 (2.16) |  | 28.39 (5.42) | 51.20 (52.97) | 4.39 (1.27) |
| El Amrousy 2022 | Vit D | 10.6 (2.02) | 49 (49) | 33.1 (1.3) | 61.1 (35.9) | 4.22 (0.8) |
|  | Placebo | 10.9 (2.5) |  | 32.1 (1.4) | 57.9 (36.6) | 4.1 (0.7) |
| Ghergherehchi 2013 | Vit E | 7.66 (3.1) | 18 (55) | 32.66 (8.1) | 75.29 (8.54) | N/A |
|  | Placebo | 7.14 (3.19) |  | 32.69 (6.04) | 75.25 (8.86) |  |
| Homaei 2022 | Metformin | 11.8 (1.6) | 79 (53) | 28.9 (4.4) | 55.72 (12.7) | N/A |
|  | Vit E | 12.1 (1.7) |  | 28.5 (3.3) | 55.38 (17.7) |  |
|  | Placebo | 12.2 (1.5) |  | 28.6 (5.3) | 58.57 (17) |  |
| Janczyk 2015 | Omega-3 | 13.4 (3.6) | 65 (86) | 29 (4.5) | 77 (24.68) | N/A |
|  | Placebo | 12.9 (3.1) |  | 28.8 (4.6) | 89.67 (46.95) |  |
| Lavine 2011 | Vit E | 13.4 (2.3) | 140 (80.9) | 34 (7) | 121 (65) | 4.8 (1.6) |
|  | Metformin | 13.1 (2.4) |  | 34 (7) | 121 (68) | 4.5 (1.2) |
|  | Placebo | 12.9 (2.6) |  | 33 (6) | 126 (62) | 4.6 (1.3) |
| Nadeau 2009 | Metformin | 15.1 (1.34)^a^ | 17 (34) | 39.6 ( 5.96) | 61.5 (4) | N/A |
|  | Placebo |  |  | 40.2 (6.49) | 52.9 (8.2) |  |
| Nobili 2008 | Vit E + Vit C | 11.87 (8.96) | 37 (69.8) | 25.47 (8.1) | 68.53 (33.34) | 4.33 (3.93) |
|  | Placebo | 12.23 (9.84) |  | 26.1 (8.83) | 57.11 (23.63) | 4.33 (3.91) |
| Nobili 2013 | Placebo | 14.33 (3.19) | 25 (41.66) | 27.8 (4.07) | 90.33 (29.52) | N/A |
|  | DHA 250 mg/day | 12 (2.39) |  | 28.23 (3.91) | 78.33 (19.95) | 3.71 (1.78) |
|  | DHA 500 mg/day | 11.67 (1.6) |  | 25.6 (2.87) | 66 (21.54) | N/A |
| Nobili 2019 | Vit E + HXT | 12.5 (2.9) | N/A | 27.4 (4.4) | 47.7 (44.6) | N/A |
|  | Placebo | 12.9 (2.7) |  | 27.4 (5.4) | 38.3 (22.9) |  |
| Ostadmohammadi 2024 | Zinc | 14.2 (2.6) | 29 (55.7) | 29.2 (2.7) | 49.7 (10.2) | N/A |
|  | Placebo | 13.9 (2.7) |  | 29.7 (2.2) | 51.4 (12) |  |
| Pacifico 2015 | DHA | 11 (2.6) | 30 (58.8) | 28.9 (4.3) | 57 (20) | 4.6 (0.5) |
|  | Placebo | 10.8 (2.8) |  | 27.5 (5.5) | 56 (19) | 4.4 (0.6) |
| Saneian 2021 | L-Carnitine | 12.3 (2.04) | 42 (76.3) | 26.7 (5.2) | 54.6 (24.5) | N/A |
|  | Placebo | 12.9 (2.41) |  | 26.7 (4.3) | 55.6 (20.6) |  |
| Schwimmer 2016 | CBDR | 13.8 (2.9) | 119 (70) | 33 (7) | 140 (118) | 4.7 (1.4) |
|  | Placebo | 13.6 (2.5) |  | 32 (6) | 103 (76) | 4.6 (1.4) |
| Tock 2010 | Metformin | N/A | 35 (100) | 37.24 (3.44) | 52.05 (26.89) | N/A |
|  | Placebo |  |  | 37.02 (3.15) | 52.12 (24.93) |  |
| Tovar 2024 | L-Citrulline | N/A | N/A | 34.93 (12.91) | 37.2 (53.9) | N/A |
|  | Placebo |  |  | 33 (3.3) | 42.5 (27.2) |  |
| Vajro 2004 | Vit E | 10.7 (3.45) | 21 (75) | 24.50 (2.78) | 72.58 (17.46) | N/A |
|  | Placebo | 9.88 (3.97) |  | 24.82 (2.35) | 76 (23.45) |  |
| Vos 2022 | Losartan | 14 (2) | 67 (80.7) | 34 (5) | 115 (50) | 4.6 (1.5) |
|  | Placebo | 13 (2) |  | 35 (5) | 126 (61) | 4.4 (1.2) |
| Wang 2008 | Vit E | 13.4 (1.6) | 52 (68.42) | 29.36 (3.11) | 139.98 (19.82) | N/A |
|  | Untreated | 13.4 (2.5) |  | 29.61 (1.48) | 152.26 (49.3) |  |
| Xue 2022 | rhGH | 12.04 (1.56) | 44 (100) | N/A | 27.44 (8.18) | N/A |
|  | Untreated | 11.48 (1.78) |  |  | 32.58 (11.32) |  |
| Zahmatkesh 2024 | Orlistat | 14.14 (1.83) | 30 (56.6) | 27.10 (2.12) | 50.29 (18.25) | 4.72 (1.36) |
|  | Placebo | 13.38 (1.61) |  | 26.7 (2.16) | 53 (16.84) | 4.44 (1.18) |
| Zöhrer 2017 | DHA + CHO + Vit E | 13.2 (2.3) | 24 (60) | 27.6 (4) | 53.5 (32.6) | 4.35 (1.1) |
|  | Placebo | 13.2 (2.1) |  | 28.3 (5.3) | 51.2 (51.6) | 4.55 (1.28) |

N= Number, BMI= Body Mass Index, ALT= Alanine Transaminase, NAS= NAFLD Activity Score, Vit= Vitamin, NAC= N-acetylcysteine, DHA= Docosahexaenoic Acid, EPA= Eicosapentaenoic Acid, HXT= Hydroxytyrosol, CBDR= Cysteamine Bitartrate Delayed Release, rhGH= recombinant human Growth Hormone, CHO= Choline.
^a^ Age is presented as the combined mean (±SD) for both groups.
^b^ NAS is presented as range (min-max); other variables as mean (SD) unless otherwise noted.

Table S3, Summary of included studies.

| Study ID | Doses | Outcome timepoints Months | MASLD diagnosis | Sample size | Design | ITT or PP | Population | Follow up duration (months) | Background medications status | Location (by continent) | Lifestyle interventions | | |
| --- | --- | --- | --- | --- | --- | --- | --- | --- | --- | --- | --- | --- | --- |
|  |  |  |  |  |  |  |  |  |  |  | Diet | Physical excercise | Additional data (sleep,etc) |
| Akcam 2011 | metformin: 850-mg daily and vitamin E: 400 U daily, in capsule form | 6 | US | 67 | randomized, double blinded, parallel group, prospective clinical trial | PP | 67 adolscent with liver steatosis with mean age 12.3(1.7) were recruited to pediatrics endocrinology unit. | 6 | Patients were explicitly excluded if they were taking medications known to influence body composition, insulin action, or insulin secretion (e.g., glucocorticoid therapy) or any medication that influences bone metabolism (hormone replacement therapy, calcitonin, bisphosphonates, cytotoxics, antimetabolites, anticoagulants, anticonvulsants, thyroxine, interferon, or lamivudine). | EU | Patients in all groups were advised to adopt a diet supplying 30 kcal/kg based on current body weight; 50 % of the diet's energy was derived from carbohydrates, 30 % from lipids, and 20Yo fromproteins | All patients were advised to perform at least 30 minutes of aerobic physical activity per day. This was evaluated as the number of weeks per month with at least 4 days/week of 30 minutes of physical activity per day. Compliance with the exercise program was considered successful if the patient completed 4 days with 30 minutes of exercise per week. | N/A |
| Al-Baiaty 2024 | tocotrienol-rich fraction vitamin E (TRF) pill containing 50 mg of vitamin E isomers, comprised 7.4 mg of δ-tocotrienol, 2.02 mg of β-tocotrienol, 22.3 mg of γ-tocotrienol, 18.28 mg of α-tocotrienol, and 17.1 mg of α-tocopherol. | 6 | US + fibroscan | 29 | randomized, single-blinded clinical trial | PP | Children between the ages of 10 and 18  who were overweight or obese (BMI between the 85th and 95th percentile for their age) were enrolled at Universiti Kebangsaan Malaysia Medical Centre (UKMMC). An abdominal ultrasound confirmed the diagnosis of NAFLD. Also, a controlled attenuation parameter (CAP) score on the FibroScan must be > 263 dB/m, with an elevated alanine transaminase level of≥two-fold the upper limit (26 U/L for boys and 22 U/L for girls, respectively). | 6 | Prior to the start of the trial, the children were instructed to avoid using any dietary supplements for at least four weeks. | Asia | Over a six-month duration, participants were directed to ingest either the oral TRF (50 mg)  or a placebo after meals.The children were advised to maintain their regular dietary habits, refrain from incorporating vitamin E-rich supplements | not to alter their exercise routines. | N/A |
| Balagopal 2024 | NAC (either 600 mg per day [n = 5] or 600 mg/twice a day [n = 5]) or placebo (n = 4) | 4 | biopsy | 13 | randomized double‐blind placebo‐controlled study design was used and the study was quadruple masked (participant, care provider, investigator, outcomes assessor) | PP | Children with age 8–18 years, BMI‐percentile ≥95th percentile and biopsy‐ confirmed MASLD (NAS score >2) were eligible for inclusion in the study. | 4 | Exclusion criteria include medications such as adrenergic β‐blockers; steroids and other drugs known to interfere with the measurement of liver enzymes and risk factors for cardiovascular disease. | NA | All participants across the groups were instructed to maintain their normal dietary intake and keep a diet diary for three consecutive days before the collection of the baseline samples | N/A | N/A |
| Boyraz 2015 | 1000 mg dose of PUFA once daily | 12 | US | 108 | patients randomized to two groups  the first recieved PUFA and the other group recieved placebo. | PP | One hundred and thirty-eight obese patients with NAFLD  (65 girls and 73 boys, aged 9 to 17 years, mean age: 13.9±3.7  years) attending the outpatient clinic of the İstanbul Şişli Etfal  Education and Research Hospital from March 2010 to June  2012 were initially selected for the stud | 12 | NA | EU | The recommended diet was composed of 50%  carbohydrates, 20% protein and 30% fat, in accordance with  the American Heart Association diet. All obese patients were  advised to lose weight with a restriction of daily caloric intake to  25-30 kcal/kg per day | The lifestyle intervention programme  consisted of scheduled exercise (three times per week for 1  hour) and the promotion of self-initiated physical activities | N/A |
| Corte 2016 | oral dose of 500 mg of DHA and 800 IU/day of vitamin D | 6 - 12 | biopsy | 41 | a randomized, double-blind placebo-controlled trial | ITT | Sixty six white European patients (4–16 years) with liver biopsy-proven NAFLD, referred to the Hepato-Metabolic Department of “Bambino Gesu`” Children’s Hospital (Rome, Italy) between March 2014 and April 2015 | 12 | Patients taking drugs known to induce fatty liver were excluded from the study. | EU | all patients were included in a lifestyle intervention program consisting of a hypocaloric diet (25–30 Kcal/kg/day) and regular physical exercise (twice weekly 1-hour physical activity) |  | N/A |
| El Amrousy 2022 | 2000 IU of vitamin D3 daily | 6 | biopsy | 100 | A randomized, double-blind, placebo-controlled trial. | PP | from those attending the outpatient endocrinology clinic,  Pediatric Department, Tanta University Hospital, during the period from September 2017 to October 2018. obese children less than 18 years with  biopsy-proven NAFLD and serum 25 (OH) D level less than  20 ng/ml. Obesity is defned as body mass index (BMI)≥30 or≥95th percentile for age and sex | 6 | Patients were excluded if they had received vitamin D supplementation in the last six months or were undergoing treatment with agents known to affect calcium or vitamin D metabolism, such as anticonvulsants and glucocorticoids. | Africa | all included children were put on the same hypocaloric diet where 30% of the total energy intake was reduced. The hypocaloric diet consists of 50% carbohydrate, 30% fat, and 20% protein | N/A | N/A |
| Ghergherehchi 2013 | vitamin E 400mg/d | 6 | US | 33 | randomized double-blind study | PP | obese children aged 4 to 15 years with NAFLD in Tabriz-Iran,  from April 2008 to June 2009  Inclusion criteria were a body mass index  higher than 97th percentile for age and sex, alanine aminotransferase (ALT), aspartate aminotransferase (AST) levels  at least 1.5 times more than the upper limit of normality  (5-40 IU/L) and signs of hepatic steatosis in ultrasonography. | 6 | Patients taking drugs known to induce steatosis, specifically prednisolone, valproate, amiodarone, and methotrexate, were excluded from the study. | Asia | The lifestyle intervention program consisted of low calorie diet  from 1300 to 1800 Kcal based on the individual requirements | aerobic exercise  and walking for 2 hours every day. | N/A |
| Homaei 2022 | metformin Glucophage (Merck) at a dose of 500 mg twice daily. Patients in the second group were treated with vitamin E capsules (EuRHO VITAL) at a dose of 400 units twice daily | 3 | US | 150 | Randomized Clinical Trial | PP | 150 nondiabetic obese children with nonalcoholic fatty liver. Samples were taken from children admitted to the endocrinology clinic of the Children’s Hospital in Qazvin, Iran, who were referred for diagnostic-therapeutic measures from 2018 to 2019. Inclusion criteria consisted of patient satisfaction, age between 10 and 14 years, the body mass index (BMI) above 95% , the nonalcoholic fatty liver disease, which was diagnosed with increased serum ALT level ( > 60 U/L), and confirmation of fatty liver with ultrasonography . | 3 | Patients with a history of taking drugs that can increase liver enzymes, such as nonsteroidal anti-inflammatory drugs, antibiotics, anticonvulsants, and statins, were excluded from the study. | Asia | all the patients were given a proper diet and advised to increase their physical activity. Recommendations for changing lifestyle, exercising, and following diets appropriate for weight loss were made to all the three groups by a nutritionist | N/A | N/A |
| Janczyk 2015 | fish oil containing omega-3 LC-PUFA (DHA and EPA in a 3:2 proportion [450-1300 mg/day]) or placebo (sunflower oil, containing omega-6 LC-PUFA) | 6 | US or biopsy | 64 | multicenter, randomized, double-blind, placebocontrolled clinical trial | ITT | 4 Polish pediatric departments, The inclusion criteria were: (1) age >5 and <19 years; (2) overweight or obesity (according to International Obesity Task Force body mass index (BMI) charts7); (3)ALT activity $1.3 times the upper limit of normal (ULN); (4) presence of hyperechogenic liver on ultrasound or liver histology consistent with NAFLD/nonalcoholic steatohepatitis | 6 | Patients were excluded if they received treatment with vitamin E, statins, antihypertensives, ursodeoxycholic acid, probiotics, or metformin within 3 months prior to randomization. | EU | , all patients were regularly instructed by an experienced dietician in each center to comply with an individually prescribed diet, which, in combination with increased physical activity, was aimed at producing a slow reduction in body weight (approximately 0.5 kg/week). Extensive dietary data were collected using a validated food frequency questionnaire | N/A | N/A |
| Lavine 2011 | Group (1) oral metformin (500 mg twice daily) and vitamin E placebo twice daily, Group (2) vitamin E (400 IU twice daily) and metformin placebo twice daily | 24 | biopsy | 173 | TONIC is a randomized, multicenter, double-masked, placebo-controlled trial of 96 weeks of treatment with metformin or vitamin E. | ITT | Eligible children were identified and recruited from unsolicited referrals to the 10 participating clinical centers starting in September 2005 and ending September 2007. Patients aged 8 to 17 years with NAFLD and persistently elevated levels of alanine aminotransferase (ALT) were eligible. All patients and parents were provided uniform standard-of-care advice on diet and exercise at each visit by physicians and dieticians. NAFLD was defined by a liver biopsy demonstrating more than 5% steatosis within a 6-month period before randomization. Persistent elevation of ALT was defined by a value greater than 60 U/L for 1 to 6 months before and at the time of randomization. Patients with diabetes mellitus or cirrhosis were excluded | 24 | Patients were excluded if they had used drugs historically associated with NAFLD for more than 2 consecutive weeks in the past 2 years, or had used antidiabetic drugs, anti-NAFLD drugs (metformin, vitamin E, UDCA, SAM-e, betaine, milk thistle, probiotics), or any over-the-counter or herbal remedy for hyperlipidemia in the 3 months prior to randomization. | NA | Eligible children were identified and recruited from unsolicited referrals to the 10 participating clinical centers starting in September 2005 and ending September 2007. Patients aged 8 to 17 years with NAFLD an persistently elevated levels of alanine aminotransferase (ALT) were eligible. All patients and parents were provided uniform standard-of-care advice on diet and exercise at each visit by physicians and dieticians. NAFLD was defined by a liver biopsy demonstrating more than 5% steatosis within a 6-month period before randomization. Persistent elevation of ALT was defined by a value greater than 60 U/L for 1 to 6 months before and at the time of randomization. Patients with diabetes mellitus or cirrhosis were excluded | N/A | N/A |
| Nadeau 2009 | Subjects were started initially on 500 mg of metformin or placebo once daily, increased to 500 mg twice daily at 1 month and then to 850 mg twice daily at 2 months | 6 | US | 50 | randomized, double-blind, placebo-controlled design | PP | The study included 50 obese adolescents, ages 12–18 yr, recruited from an urban health care system including school-based health centers providing health care to the medically underserved in Denver, CO, through posted advertisements or contact with providers as previously described (27). A screening visit included family history, physical exam, and fasting laboratory evaluation. Eligibility criteria included fasting insulin level .25 microunits/mL or homeostasis model assessment .3.5 (28) and two of three of the following: acanthosis nigricans, body mass index (BMI) .30 kg/m2 or .95% for age, or family history of type 2 diabetes."Exclusion criteria included preexisting diabetes, pregnancy, serum creatinine .1.5 mg/dL, current or prior alcohol consumption, or significant heart disease. Liver disease other than NAFLD was excluded by the following labs: hepatitis B surface antigen, hepatitis C antibody, ceruloplasmin, alpha-1 antitrypsin level or phenotype, prothrombin time, iron, total iron binding capacity, ferritin, and anti-nuclear, anti-smooth muscle, and anti-liver/kidney microsomal antibodies. No other abnormal labs or causes of liver disease requiring exclusion were identified." | 6 | NA | NA | All subjects underwent dietary assessment, and watched a standardized video about healthy eating habits. Wellness education emphasized balanced meals with modest calorie reduction; decreased fat and simple sugar consumption; increased fiber, fruit, and vegetable intake; and regular aerobic exercise. Subjects chose three specific dietary or exercise goals and were given a calendar to record progress on their goals and medication compliance | N/A | N/A |
| Nobili 2008 | vitamin E 600 IU/day and vitamin C 500 mg/day | 12 - 24 | biopsy | 53 | open-label fashion | ITT | Inclusion criteria were persistently elevated serum aminotransferase levels, diffusely echogenic liver on imaging studies suggestive of fatty liver, and biopsy consistent with the diagnosis of NAFLD. Exclusion criteria were hepatic virus infections (HCV RNA–polymerase chain reaction negative; hepatitis A, B, C, D, E, and G; cytomegalovirus; and Epstein-Barr virus), alcohol consumption, history of parenteral nutrition, and use of drugs known to induce steatosis (for example, valproate, amiodarone, or prednisone) or to affect body weight and carbohydrate metabolism. Autoimmune liver disease, metabolic liver disease, Wilson’s disease, and -1-antitrypsin-associated liver disease were ruled out. | 24 | Patients were excluded if they were taking drugs known to induce steatosis (e.g., valproate, amiodarone, or prednisone) or medications that affect body weight and carbohydrate metabolism. | EU | Lifestyle Intervention. Patients and responsible guardians underwent monthly 1-hour nutritional counseling by the same experienced dietician during the 24- month treatment period. At every visit, participants were encouraged to continue adherence to treatment. Diet was hypocaloric (25-30 calories/kg/day) in overweight and obese children; and isocaloric (40-45 calories/kg/day) in children with normal BMI. The number of calories needed was determined taking into account anthropometrics, and physical and daily activities. Diet composition consisted of carbohydrate (50%-60%); fat (23%- 30%); and protein (15%-20%); fatty acid composition was two-thirds unsaturated, and one-third saturated; the 6/3 ratio was 4:1 as recommended by the Italian Recommended Dietary Allowances. Diet was tailored on individual preferences and requirement of calories. The goal of weight management was to induce a negative calorie balance in overweight and obese patients and to allow a neutral calorie balance in normal weight subjects. The above diet regimen was prescribed with a recommendation to engage in a moderate daily exercise program (45 minutes/day aerobic physical exercise). At each visit, subjects or their responsible guardians were asked to fill out a 3-day dietary and physical activity recall to evaluate adherence to lifestyle recommendations. A multidisciplinary team including dieticians, hepatologists, endocrinologists, psychologists, and cardiologists evaluated and closely followed-up patients participating in this study as done routinely in overweight and obese children and adolescents treated in our institution. To increase compliance with the recommended treatment and weight maintenance during follow-up, the weight loss program focused on long-term dietary modification (low-fat meals, decrease of nutrient dense foods and consequent increase of fruits and vegetables, ingestion of small-size to moderate-size portions of meals throughout the day), increment in daily physical activity and reduction of sedentary activities, and behavior change skills (self-monitoring, familybased reinforcement systems, identification of high-risk situations, self-awareness, stimulus controls, and cognitive behavior strategies). Participants and their family were instructed on how to exercise and maintain adherence to the exercise program by a skilled exercise physiologist as part of this multidisciplinary program. | N/A | N/A |
| Nobili 2013 | DHA 250 mg/day, and DHA 500 mg/day | 6 - 12 -18 - 24 | biopsy | 60 | randomized controlled trial of DHA supplementation (250 mg/day and 500 mg/day) vs. placebo | ITT | Children were eligible for the study if they had: 1) age <18 years, 2) persistently elevated serum alanine transaminase (ALT 40 U/L), 3) diffusely hyperechogenic liver at ultrasonography, and 4) liver biopsy consistent with NAFLD. Children were excluded from the study if they had any of the following: 1) viral liver disease, 2) autoimmune liver disease, 3) Wilson’s disease, 4) a-1-antitrypsin deficiency, 5) celiac disease, 6) alcohol consumption (any quantity), 7) use of parenteral nutrition, 8) use of drugs known to induce fatty liver, and 9) previous use of N3-LCPUFA | 24 | Patients were excluded for the use of drugs known to induce fatty liver or for the previous use of n-3 long-chain polyunsaturated fatty acids. | EU | A balanced lowcalorie diet was prescribed and physical activity was suggested to all patients as described in detail elsewhere | Nutritional counselling consisted of a low-calorie diet for subjects with a BMI ‡ 85th percentile and an iso-calorie diet for normal weight subjects. Patients and responsible guardians underwent 1-hour nutritional counselling monthly by the same experienced dietician. Diet was tailored on individual preferences and balanced as recommended by the Italian Recommended Dietary Allowances. (hypo-caloric diet 25–30 cal/kg/day, iso-caloric diet 40–45 cal/kg/day, carbohydrate 50–60%; fat 23–30%; protein 15–20%; fatty acid: two-third saturated, onethird unsaturated; x6/x3 ratio ¼ 4:1). The goal of weight management was to induce a negative calorie balance in subjects with a BMI‡ 85th percentile and to allow a balanced diet in normal weight subjects. The above diet regimen was associated with a recommendation to engage in a moderate daily exercise programme (45 min/day aerobic physical exercise) | N/A |
| Nobili 2019 | 7.5 mg of HXT and 10 mg of vitamin E | 4 | biopsy | 70 | randomized double-blind placebo-controlled trial | ITT | Eighty-one white European patients (4–16 years) with NAFLD, referred to the HepatoMetabolic Department of “Bambino Gesù” Children’s Hospital (Rome, Italy) between March 2017 and April 2018, were enrolled in the present study. The diagnosis of NAFLD was performed according to the guidelines of the European Society of Pediatric Gastroenterology, Hepatology and Nutrition (ESPGHAN). The children were eligible for the study if they were between 4 and 16 years of age, had liver steatosis, aminotransferases (ALT) levels <10 upper limit of normal, and no laboratory and/or clinical signs of liver decompensation. Moreover, in all children other causes of liver disease (i.e. viral liver disease, autoimmune hepatitis, Wilson's disease, α-1-antitrypsin deficiency), celiac disease, alcohol consumption, use of drugs known to induce fatty liver, were excluded | 4 | Patients taking drugs known to induce fatty liver were excluded from the study. | EU | , all patients were included in a lifestyle intervention program consisting of a hypocaloric diet (25–30 Kcal/kg/day) and regular physical exercise (twice weekly 2-hour physical activity) | N/A | N/A |
| Ostadmohammadi 2024 | 30 mg of elemental zinc | 4 | US | 52 | a randomized, double-blind, placebo-controlled trial with a parallel design | PP | Inclusion criteria included overweight or obese children (age- and sex-specific BMI of ≥ 85th percentile) with NASH, aged 10–18 years. NASH was diagnosed by ultrasonography based on the American Society of Pediatric Gastroenterology criteria and serum concentration of alanine aminotransferase (ALT) [2, 24]. Serum ALT concentrations greater than 26 U/L for girls and 22 U/L for boys were utilized as diagnostic criteria for NASH [25]. Participants were excluded if they had any infectious or autoimmune diseases, other conditions that can cause an elevation in serum ALT levels such as viral hepatitis (B and C), hemochromatosis, HIV, a-1-anti-trypsin deficiency, and Wilson disease, hypersensitivity to the study supplement, and used any antioxidant and/or anti-inflammatory agents within 3 months before enrollment. | 4 | Patients who had used any antioxidant and/or anti-inflammatory agents within 3 months before enrollment were excluded. | Asia | each participant’s physical activity levels and dietary intake were not evaluated | N/A | N/A |
| Pacifico 2015 | DHA supplementation [250 mg/day] | 6 | biopsy | 51 | double-blind, placebo-controlled randomized trial | PP | Patients were eligible for the study if they had at enrollment: 1) age < 18 years; 2) body mass index (BMI) > 85th percentile according to age- and gender-specific percentiles of BMI [17]; 3) persistently elevated aminotransferase levels; 4) magnetic resonance imaging (MRI)-diagnosed NAFLD [hepatic fat fraction (HFF) ≥ 5%] [18]; and 5) liver biopsy consistent with NAFLD. Secondary causes of steatosis including hepatic virus infections (hepatitis A-E and G, cytomegalovirus, and Epstein-Barr virus), autoimmune hepatitis, metabolic liver disease, α-1-antitrypsin deficiency, cystic fibrosis, Wilson’s disease, hemochromatosis, and celiac disease were excluded after appropriate tests. Other exclusion criteria were smoking, and the history of type 1 or type 2 diabetes, renal disease, total parenteral nutrition, alcohol intake, use of hepatotoxic medications, and previous use of n-3 LC-PUFAs. | 6 | Patients were excluded for the use of hepatotoxic medications and the previous use of n-3 long-chain polyunsaturated fatty acids. | EU | A balanced low-calorie diet was prescribed to all patients with a recommendation to engage in a moderate daily exercise program (60 min/day at least 5 days a week), and to reduce sedentary activities. Specifically, diet was hypocaloric (25-30 calories/Kg/day), consisting of carbohydrate (50%-60%); protein (15%-20%); and fat (23%-30%), with a composition of two in third unsaturated and one in third saturated; the ω6/ω3 ratio was approximately 4:1 as recommended by the Italian Recommended Dietary Allowances | N/A | N/A |
| Saneian 2021 | vitamin E 400 unit per day, L-carnitine tablet with dose of 50 mg/kg/day twice a day (max: 500 mg Bid) | 3 | US | 55 | randomized, triple-blind, placebo-controlled clinical trial | PP | Eligibility criteria for participation were as follows: patients with NAFLD, increase liver enzymes more than 40 unit/L and diagnosis of fatty liver based on ultrasound, patients between the ages of 5 and 15 years. Patients with auto immune hepatitis, hypothyroidism, Wilson disease, viral hepatitis, hemochromatosis, gastrointestinal disorders, alcohol consumption, use of drugs that might affect liver functions and participants with low compliance for following the study and failure to complete medication period (3 months) were excluded. | 3 | Patients taking drugs that might affect liver functions were excluded from the study. | Asia | Both groups were advised to exercise, weight control and low fat diet. For this purpose, all patients were referred to the nutritionist. | N/A | N/A |
| Schwimmer 2016 | CBDR (75-mg capsules) or matching placebo capsules orally in weight-adjusted doses: 300 mg twice daily if weighing 65 kg or less, 375 mg twice daily if weighing more than 65 to 80 kg, or 450 mg twice daily if weighing more than 80 kg. The treatment duration was 52 weeks. Participants started treatment at a lower dose (75 mg twice daily for 65 kg, 150 mg twice daily for >65 to 80 kg, or 225 mg twice daily for >80 kg), which escalated weekly to the full dose during the first 4 weeks | 13 | biopsy | 119 | CyNCh was a multicenter, placebo controlled, randomized clinical trial of children ages 8–17 years with moderate to severe NAFLD | ITT | Study inclusion criteria were as follows: children ages 8–17 years at enrollment, with histologic evidence of NAFLD based on a liver biopsy specimen obtained within 90 days of the start of screening and no more than 120 days before randomization, and a NAFLD activity score (NAS) of 4 or higher, as scored by the individual NASH CRN pathology committee member at each study site. The median number of days between liver biopsy and randomization was 74 (interquartile range, 51–96 days). Participants also lacked evidence of other liver diseases based on laboratory evaluation and liver histology. They also had to show the ability to swallow CBDR capsules. Children were excluded if they had uncompensated liver disease, poorly controlled diabetes (hemoglobin A1c > 9%), or a history of other conditions that made it unsafe to participate | 13 | Patients were excluded for chronic use (more than 2 consecutive weeks in the past year) of medications known to cause hepatic steatosis or steatohepatitis (systemic glucocorticoids, tetracycline, anabolic steroids, valproic acid, salicylates, tamoxifen), use of other known hepatotoxins within 90 days of biopsy, or initiation of NAFLD/NASH treatment between biopsy and randomization. | NA | All children received a standardized nutrition and exercise intervention consistent with the American Academy of Pediatrics 2007 Expert Committee Recommendations Regarding the Prevention, Assessment, and Treatment of Child and Adolescent Overweight and Obesity.23 As recommended for NAFLD, lifestyle advice was provided at each study visit.24 | N/A | N/A |
| Tock 2010 | metformin treated group (500 mg twice daily) | 6 -12 | US | 35 | Thirty-five postpubertal obese boys were randomized into two groups | PP | The boys included in the study were between 15 and 19 years old, presented with primary obesity (body mass index >95th percentile of the CDC reference growth charts) [12], and had reached the initial postpubertal Tanner stage five | 12 | NA | SA | They were encouraged to reduce their food intake and follow a specifically balanced diet, reducing saturated fats and refined sugars | aerobic training program comprised of 60-minute sessions, three times a week (180 minutes/week) | N/A |
| Tovar 2024 | 6 g/day of L-citrulline | 2 | US | 42 | A prospective, double-blind clinical trial in adolescents with abdominal obesity was randomized into two groups | PP | aged between 15 and 19 years with overweight or obesity by body mass index (BMI) for age according to the World Health Organization (WHO), who were stage 5 on the Tanner scale, who had a diagnosis of MAFLD (measuring steatosis by ultrasound) [9], and who had no significant alcohol consumption | 2 | NA | NA | the participants were asked not to change their diet | N/A | N/A |
| Vajro 2004 | oral a-acetate tocopherol pills (400 mg/d; 8 ± 3.5 mg/kg body weight/once a day; range, 4.4–12 mg/kg body weight/once a day for 2 months, followed by 100 mg/d for 3 months and thereafter by 2 months of washout) | 2 - 5 | US + biopsy (not to all) | 28 | Twenty-eight children with obesity-related hypertransaminasemia and bright liver were randomly allocated to two single-blind groups | ITT | (ideal body weight $ 120% according to Tanner (25), and body mass index [BMI, Kg/m2] (26) > 95th percentile) patients seen at our institution from January 1999 to June 2001 because of chronic (> 6 months’ duration) hypertransaminasemia (aspartate aminotransferase or alanine aminotransferase [ALT] $ 1.5 times above normal values for more than 6 months] and US hepatic steatosis (bright liver) | 5 | None of the patients had previously been treated with hepatotoxic drugs. | EU | Patients received the prescription of a balanced lowcalorie diet (30 cal/kg/d, carbohydrate 55%–65%; fat 23%–30%, protein 12%–15%; fatty acids: two thirds saturated, one third unsaturated; v6/v3 ratio 4 4:1, as recommended by Italian Recommended Dietary Allowances ), and moderate daily exercise program, with a target weight loss of 2 kg/month for 6 months. | Physical exercise was also recommended to all patients | N/A |
| Vos 2022 | The starting dose was one 50-mg capsule of losartan or matching placebo per day for 1 week, then two capsules of 50 mg of losartan or matching placebo once per day (100 mg total) from weeks 2– 24 | 6 | biopsy | 83 | a multicenter, randomized, doublemasked, placebo-controlled, parallel treatment group phase 2 trial | PP | age 8–17 years at initial screening, (2) histologic evidence of NAFLD with or without fibrosis and a NAFLD activity score (NAS) of ≥3 (without requirement for all three components of the NAS, other than steatosis) on liver biopsy that predated enrollment by no more than 2 years, and (3) serum ALT at screening ≥50 U/l | 6 | Summary Sentence: Patients were excluded if receiving treatment with antihypertensive medication, potassium, nonsteroidal anti-inflammatory drugs (NSAIDs), or lithium; however, a minority of patients (5% to 7%) across groups had a history of using anti-lipidemic or anti-psychotic medications in the past 6 months. | NA | At each study visit, trained study staff provided standardized evidence-based, written nutrition and exercise recommendations (Appendix S3) to all participants, in accordance with the current standard-of-care lifestyle intervention for pediatric NAFLD | N/A | N/A |
| Wang 2008 | vitamin E capsule at a dose of 100 mg/d for one month | 1 | US | 76 | divided randomly into three groups and the ultrasonography operator was blinded to the groups | PP | child was considered to be obese when the body mass index (BMI) exceeded the 95th BMI percentage for age and sex[20], were enrolled in this study. The age of the subjects ranged from 10 to 17 years (mean 13.7 ± 1.9 years). They were all obese children with liver fatty infiltration in ultrasonic appearance and abnormal liver function with higher alanine aminotransferase (ALT) by at least 1.5 times over the upper normal limit which was diagnosed as NASH | 1 | NA | Asia | Group 2: low-calorie [high in carbohydrate (50%) and low in fat (10%)] with the aim of a reduction in daily intake by 250 kcal. A total daily calorie intake was controlled from 1300 kcal to 1600 kcal based on the individual age. Two eggs and a bowl of soymilk were supplied at breakfast. Pork, egg, fish, shrimp, fresh vegetable, rice and corn were served at lunch and dinner. No beverage but mineral water was provided /// Group 3 were controlled in lifestyle freely by themselves with total daily calorie intake./// Group 1 did not receive any  lifestyle intervention. | Group 2: swimming, playing basketball and tabletennis Group 3: physical exercises (a low-intensity aerobic exercise to reach a 50%-60% maximum of their heart beat and maintained for 30 min, 2-3 times a week) | Group 2: get up at 6: 30 O’clock in the morning and went to sleep at 21: 00 O’clock |
| Xue 2022 | Daily dose of GH was 0.1 IU/kg (0.033 mg/kg) | 3 - 6 | US | 44 | a randomized, open-label study | PP | boys with obesity and NAFLD aged 8–16 years. Exclusion criteria included: 1) secondary obesity, such as hypothyroidism, Cushing’s disease or hypothalamic obesity; 2) positive hepatitis B surface antigen, positive hepatitis C antibodies or other liver disease such as Wilson’s disease or autoimmune hepatitis etc.; 3) diabetes, hypothalamus-pituitary diseases or other disease condition known to affect the GH axis; 4) children who ever have had alcohol intake, smoked or used drugs which may influence liver function, glucose, lipid metabolism or weight within the 3 months prior to study; 5) serious infection, systemic disease, and other chronic wasting illnesses; 6) short stature or growth velocity is less than 5 cm/yea | 6 | Patients were excluded if they had used drugs that might influence liver function, glucose, lipid metabolism, or weight within the 3 months prior to the study. | Asia | guidance of dietary and exercise was according to the guideline presented by Styne et al | N/A | N/A |
| Zahmatkesh 2024 | 360 mg/day of orlistat | 3 | biopsy | 53 | a double-blind randomized clinical trial | PP | boys and girls with overweight/ obese, aged 12 to 18 years; 3) having BMI Z score higher than 1 and lower than 3 for age and sex; 4) liver biopsy showing more than 5% steatosis (NAFLD definition); 5) high serum levels of liver enzyme ALT 1.5 times the normal level | 3 | Adolescents were excluded if they had a documented medical background, were using hepatotoxic drugs (such as phenytoin, amoxifen, or lithium), or had taken pharmaceuticals, dietary supplements, or herbal supplements impacting appetite, weight, or metabolism for a minimum of 3 months prior to the study. | Asia | The distribution of caloric intake was estimated to include 30% fat (7% saturated and a maximum of 300 mg of cholesterol), 50% carbohydrate,and 20% protein, and all subjects received the same dietary recommendations. | N/A | N/A |
| Zöhrer 2017 | 250 mg of DHA, 39 IU of vitamin E and 201 mg of choline | 12 | biopsy | 40 | a randomized controlled clinical trial | PP | Excluded were patients with Wilson disease, hepatitis B and C, acute systemic disease, autoimmune hepatitis, hypothyroidism, cystic fibrosis, celiac disease, suspected muscular dystrophy, alpha-1-antitrypsin deficiency and other metabolic inherited diseases. Patients were also excluded if body weight and carbohydrate metabolism were altered by parenteral nutrition, protein malnutrition, previous gastrointestinal surgery, structural abnormalities of the gastrointestinal tract or neurological impairment. Finally, the use of nonsteroidal anti-inflammatory drugs, antibiotics, probiotics or antisecretory drugs capable of mcausing achlorhydria within the two months before enrolment was also an exclusion criterion. Forty-three of 60 screened children with NAFLD met these inclusion criteria: age 4-16 years [13 (30.2%) children and 30 (69.8%) adolescents], persistently elevated serum aminotransferase levels, diffusely echogenic liver on imaging studies suggestive of fatty liver and biopsy findings consistent with NASH. All these children were enrolled in this placebo-controlled clinical trial | 12 | The use of nonsteroidal anti-inflammatory drugs, antibiotics, probiotics, or antisecretory drugs capable of causing achlorhydria within the two months before enrolment was an exclusion criterion. | EU | Both were provided by DMF Dietetic Metabolic Food (Italy). Concomitantly, all patients were recommended to follow a hypocaloric diet (25-30 kcal/kg/day) and to engage in twice weekly 1-hour physical activity during the treatment and for further 6 months of follow-up | N/A | N/A |

MASLD= Metabolic Dysfunction-Associated Steatotic Liver Disease, US= Ultrasonography, PUFA= Polyunsaturated Fatty Acid, DHA= Docosahexaenoic Acid, IU= International Unit, LC-PUFA= Long Chain-Polyunsaturated Fatty Acid, EPA= Eicosapentaenoic Acid, HXT= Hydroxytyrosol, CBDR= Cysteamine Bitartrate Delayed Release, GH= Growth Hormone, EU= Europe, NA= North America, SA= South America, ITT = Intention-to-treat, PP = Per-Protocol.

Table S4, League table for pairwise meta-analysis (right upper part) and NMA (left lower part) effect estimates: NASH resolution events.


Table S5, League table for pairwise meta-analysis (right upper part) and NMA (left lower part) effect estimates: Fibrosis improvements events.


Table S6, League table for pairwise meta-analysis (right upper part) and NMA (left lower part) effect estimates: Fibrosis score.

Table S7, League table for pairwise meta-analysis (right upper part) and NMA (left lower part) effect estimates: Lobular inflammation improvements events.

Table S8, League table for pairwise meta-analysis (right upper part) and NMA (left lower part) effect estimates: Lobular inflammation score.

Table S9, League table for pairwise meta-analysis (right upper part) and NMA (left lower part) effect estimates: Steatosis improvement events.

Table S10, League table for pairwise meta-analysis (right upper part) and NMA (left lower part) effect estimates: Steatosis score.

Table S11, League table for pairwise meta-analysis (right upper part) and NMA (left lower part) effect estimates: Ballooning improvement events.

Table S12, League table for pairwise meta-analysis (right upper part) and NMA (left lower part) effect estimates: Ballooning score.

Table S13, League table for pairwise meta-analysis (right upper part) and NMA (left lower part) effect estimates: Portal inflammation improvement events.

Table S14, League table for pairwise meta-analysis (right upper part) and NMA (left lower part) effect estimates: Portal inflammation score.

Table S15, League table for pairwise meta-analysis (right upper part) and NMA (left lower part) effect estimates: AST (results after sensitivity analysis).

Table S16, League table for pairwise meta-analysis (right upper part) and NMA (left lower part) effect estimates: AST (results before sensitivity analysis).

Table S17, League table for pairwise meta-analysis (right upper part) and NMA (left lower part) effect estimates: ALT (results before sensitivity analysis).

Table S18, League table for pairwise meta-analysis (right upper part) and NMA (left lower part) effect estimates: ALP.

Table S19, League table for pairwise meta-analysis (right upper part) and NMA (left lower part) effect estimates: Total cholesterol (results after sensitivity analysis).

Table S20, League table for pairwise meta-analysis (right upper part) and NMA (left lower part) effect estimates: Total cholesterol (results before sensitivity analysis).

Table S21, League table for pairwise meta-analysis (right upper part) and NMA (left lower part) effect estimates: Triglycerides (results after sensitivity analysis).

Table S22, League table for pairwise meta-analysis (right upper part) and NMA (left lower part) effect estimates: Triglycerides (results before sensitivity analysis).

Table S23, League table for pairwise meta-analysis (right upper part) and NMA (left lower part) effect estimates: LDL (results after sensitivity analysis).

Table S24, League table for pairwise meta-analysis (right upper part) and NMA (left lower part) effect estimates: LDL (results before sensitivity analysis).

Table S25, League table for pairwise meta-analysis (right upper part) and NMA (left lower part) effect estimates: HDL (results after sensitivity analysis).

Table S26, League table for pairwise meta-analysis (right upper part) and NMA (left lower part) effect estimates: HDL (results before sensitivity analysis).

Table S27, League table for pairwise meta-analysis (right upper part) and NMA (left lower part) effect estimates: Fasting blood glucose (results after sensitivity analysis).

Table S28, League table for pairwise meta-analysis (right upper part) and NMA (left lower part) effect estimates: Fasting blood glucose (results before sensitivity analysis).

Table S29, League table for pairwise meta-analysis (right upper part) and NMA (left lower part) effect estimates: FBI (results after sensitivity analysis).

Table S30, League table for pairwise meta-analysis (right upper part) and NMA (left lower part) effect estimates: FBI (results before sensitivity analysis).

Table S31, League table for pairwise meta-analysis (right upper part) and NMA (left lower part) effect estimates: HOMA-IR (results after sensitivity analysis).

Table S32, League table for pairwise meta-analysis (right upper part) and NMA (left lower part) effect estimates: HOMA-IR (results before sensitivity analysis).

Table S33, League table for pairwise meta-analysis (right upper part) and NMA (left lower part) effect estimates: BMI (results before sensitivity analysis).

Table S34, League table for pairwise meta-analysis (right upper part) and NMA (left lower part) effect estimates: BMI-SDS (results after sensitivity analysis).

Table S35, League table for pairwise meta-analysis (right upper part) and NMA (left lower part) effect estimates: BMI-SDS (results before sensitivity analysis).

Table S36, League table for pairwise meta-analysis (right upper part) and NMA (left lower part) effect estimates: Waist circumference.

Table S37, Meta-regression of Body Mass Index (BMI) versus Alanine Aminotransferase (ALT): Empirical mean and standard deviation for each variable, plus standard error of the mean, across studies included in the analysis.

| Variable | Mean | SD | Naive SE | Time-series SE |
| --- | --- | --- | --- | --- |
| d.Placebo.CBDR | -43.7597 | 16.827 | 0.05949 | 0.24891 |
| d.Placebo.DHA | -8.285 | 9.008 | 0.03185 | 0.12421 |
| d.Placebo.DHA_CHO_Vit_E | -0.2907 | 17.538 | 0.06201 | 0.32943 |
| d.Placebo.DHA_Vit_D | -8.6566 | 16.065 | 0.0568 | 0.21398 |
| d.Placebo.L_Carnitine | -1.2773 | 10.669 | 0.03772 | 0.168 |
| d.Placebo.L_Citrulline | 19.0895 | 22.835 | 0.08073 | 0.49854 |
| d.Placebo.Losartan | 2.7343 | 19.196 | 0.06787 | 0.30277 |
| d.Placebo.Metformin | -2.6814 | 6.901 | 0.0244 | 0.13446 |
| d.Placebo.NAC | -12.8158 | 24.505 | 0.08664 | 0.48429 |
| d.Placebo.Omega_3 | -4.3399 | 15.483 | 0.05474 | 0.19632 |
| d.Placebo.Orlistat | -5.8646 | 10.761 | 0.03805 | 0.16407 |
| d.Placebo.Vit_D | -28.4968 | 10.425 | 0.03686 | 0.09278 |
| d.Placebo.Vit_E | -2.9769 | 5.827 | 0.0206 | 0.04345 |
| d.Placebo.Vit_E_HXT | -4.2331 | 14.681 | 0.0519 | 0.20546 |
| d.Placebo.Vit_E_Vit_C | -14.1168 | 15.193 | 0.05372 | 0.24812 |
| d.Placebo.Zinc | -10.0555 | 10.792 | 0.03815 | 0.06551 |
| sd.d | 7.9948 | 5.771 | 0.02041 | 0.17521 |
| B | -3.5456 | 8.55 | 0.03023 | 0.38364 |

Table S38, Meta-regression of Body Mass Index (BMI) versus Alanine Aminotransferase (ALT): Quantiles (2.5%, 25%, 50%, 75%, and 97.5%) for each variable across studies included in the analysis.

| Variable | 2.50% | 25% | 50% | 75% | 97.50% |
| --- | --- | --- | --- | --- | --- |
| d.Placebo.CBDR | -76.846 | -54.632 | -43.6147 | -33.1702 | -10.753 |
| d.Placebo.DHA | -26.6736 | -13.255 | -8.2547 | -3.1945 | 9.716 |
| d.Placebo.DHA_CHO_Vit_E | -34.3903 | -11.426 | -0.6978 | 10.5818 | 37.122 |
| d.Placebo.DHA_Vit_D | -40.6987 | -18.713 | -8.8072 | 1.4564 | 23.093 |
| d.Placebo.L_Carnitine | -24.5074 | -5.935 | -0.9614 | 3.616 | 20.238 |
| d.Placebo.L_Citrulline | -25.5975 | 4.212 | 19.0191 | 33.87 | 64.26 |
| d.Placebo.Losartan | -34.4109 | -9.833 | 2.7728 | 14.953 | 40.715 |
| d.Placebo.Metformin | -16.5876 | -6.327 | -2.8464 | 0.8246 | 12.044 |
| d.Placebo.NAC | -60.493 | -29.21 | -12.5959 | 3.5198 | 35.288 |
| d.Placebo.Omega_3 | -35.2915 | -14.139 | -4.6977 | 5.7013 | 25.805 |
| d.Placebo.Orlistat | -29.279 | -10.569 | -5.5373 | -0.8838 | 15.876 |
| d.Placebo.Vit_D | -49.8903 | -33.339 | -28.6193 | -23.8322 | -6.185 |
| d.Placebo.Vit_E | -15.1449 | -6.052 | -2.9586 | 0.1175 | 8.973 |
| d.Placebo.Vit_E_HXT | -33.8266 | -13.312 | -4.1401 | 5.0216 | 24.483 |
| d.Placebo.Vit_E_Vit_C | -44.924 | -23.239 | -13.8611 | -4.5893 | 15.492 |
| d.Placebo.Zinc | -32.4031 | -15.406 | -9.9196 | -4.6977 | 12.009 |
| sd.d | 0.3479 | 3.779 | 6.9408 | 18.8749 | 22.441 |
| B | -22.5599 | -8.343 | -3.154 | 1.8069 | 12.386 |

Table S39, Meta-regression of Duration versus Alanine Aminotransferase (ALT): Empirical mean and standard deviation for each variable, plus standard error of the mean, across studies included in the analysis.

| Variable | Mean | SD | Naive SE | Time-series SE |
| --- | --- | --- | --- | --- |
| d.Placebo.CBDR | -46.2579 | 14.851 | 0.05251 | 0.30656 |
| d.Placebo.DHA | -9.4638 | 7.499 | 0.02651 | 0.14229 |
| d.Placebo.DHA_CHO_Vit_E | -1.3143 | 16.023 | 0.05665 | 0.40494 |
| d.Placebo.DHA_Vit_D | -9.616 | 14.509 | 0.0513 | 0.29555 |
| d.Placebo.L_Carnitine | 2.7349 | 7.392 | 0.02614 | 0.11577 |
| d.Placebo.L_Citrulline | 20.3373 | 21.986 | 0.07773 | 0.76374 |
| d.Placebo.Losartan | 2.5424 | 17.62 | 0.0623 | 0.39368 |
| d.Placebo.Metformin | -1.7977 | 5.042 | 0.01783 | 0.07903 |
| d.Placebo.NAC | -13.1162 | 23.621 | 0.08351 | 0.64085 |
| d.Placebo.Omega_3 | -3.022 | 13.904 | 0.04916 | 0.28777 |
| d.Placebo.Orlistat | -1.9922 | 7.566 | 0.02675 | 0.11707 |
| d.Placebo.Vit_D | -28.3674 | 7.464 | 0.02639 | 0.06665 |
| d.Placebo.Vit_E | -0.6217 | 4.172 | 0.01475 | 0.06188 |
| d.Placebo.Vit_E_HXT | -0.7124 | 12.526 | 0.04429 | 0.2379 |
| d.Placebo.Vit_E_Vit_C | -18.7535 | 14.522 | 0.05134 | 0.38173 |
| d.Placebo.Zinc | -7.7016 | 8.322 | 0.02942 | 0.11535 |
| sd.d | 5.1469 | 4.439 | 0.0157 | 0.1592 |
| B | 5.5268 | 7.15 | 0.02528 | 0.27193 |

Table S40, **Meta-regression of Duration versus Alanine Aminotransferase (ALT):** Quantiles (2.5%, 25%, 50%, 75%, and 97.5%) for each variable across studies included in the analysis.

| Variable | 2.50% | 25% | 50% | 75% | 97.50% |
| --- | --- | --- | --- | --- | --- |
| d.Placebo.CBDR | -75.763 | -55.7148 | -46.2029 | -36.725 | -16.561 |
| d.Placebo.DHA | -23.666 | -14.0129 | -9.6132 | -5.099 | 6.232 |
| d.Placebo.DHA_CHO_Vit_E | -31.5868 | -12.0929 | -1.4598 | 9.292 | 30.108 |
| d.Placebo.DHA_Vit_D | -37.8755 | -18.9948 | -9.8434 | -0.523 | 19.278 |
| d.Placebo.L_Carnitine | -13.7965 | -0.2609 | 3.1073 | 6.225 | 16.852 |
| d.Placebo.L_Citrulline | -21.589 | 5.8435 | 20.3554 | 34.884 | 63.871 |
| d.Placebo.Losartan | -32.7538 | -9.1577 | 2.6789 | 14.539 | 35.986 |
| d.Placebo.Metformin | -12.8408 | -4.5727 | -1.4772 | 1.371 | 7.372 |
| d.Placebo.NAC | -60.1624 | -29.0019 | -12.8076 | 3 | 32.202 |
| d.Placebo.Omega_3 | -29.5596 | -12.4025 | -3.0939 | 6.302 | 23.474 |
| d.Placebo.Orlistat | -18.7927 | -5.1638 | -1.6352 | 1.658 | 12.468 |
| d.Placebo.Vit_D | -44.6949 | -31.6314 | -28.2196 | -24.776 | -13.41 |
| d.Placebo.Vit_E | -9.4258 | -2.9977 | -0.4534 | 1.848 | 7.261 |
| d.Placebo.Vit_E_HXT | -26.1953 | -8.6469 | -0.1693 | 7.386 | 22.731 |
| d.Placebo.Vit_E_Vit_C | -45.8322 | -28.425 | -19.1876 | -9.746 | 11.517 |
| d.Placebo.Zinc | -25.4999 | -11.935 | -7.4547 | -3.091 | 8.282 |
| sd.d | 0.1729 | 1.8426 | 3.9816 | 7.241 | 16.443 |
| B | -10.2684 | 1.3101 | 5.8682 | 10.277 | 18.703 |

Table S41, Meta-regression of AST versus Body Mass Index (BMI): Empirical mean and standard deviation for each variable, plus standard error of the mean, across studies included in the analysis.

| Variable | Mean | SD | Naive SE | Time-series SE |
| --- | --- | --- | --- | --- |
| d.Placebo.CBDR | -26.2994 | 9.658 | 0.03415 | 0.15135 |
| d.Placebo.DHA_CHO_Vit_E | -7.7309 | 11.573 | 0.04092 | 0.24439 |
| d.Placebo.DHA_Vit_D | -11.1879 | 11.669 | 0.04126 | 0.21776 |
| d.Placebo.L_Carnitine | -0.7488 | 7.193 | 0.02543 | 0.18286 |
| d.Placebo.L_Citrulline | -14.3439 | 15.211 | 0.05378 | 0.35044 |
| d.Placebo.Losartan | 5.815 | 10.362 | 0.03663 | 0.18596 |
| d.Placebo.Metformin | -2.2077 | 4.498 | 0.0159 | 0.12682 |
| d.Placebo.NAC | -13.6711 | 13.104 | 0.04633 | 0.27926 |
| d.Placebo.Omega_3 | -8.0899 | 8.262 | 0.02921 | 0.11413 |
| d.Placebo.Orlistat | -4.5465 | 7.155 | 0.0253 | 0.16301 |
| d.Placebo.Vit_D | -13.5241 | 6.786 | 0.02399 | 0.06255 |
| d.Placebo.Vit_E | -3.107 | 4.976 | 0.01759 | 0.06071 |
| d.Placebo.Vit_E_HXT | -18.3422 | 9.489 | 0.03355 | 0.17248 |
| d.Placebo.Vit_E_Vit_C | -10.7565 | 9.716 | 0.03435 | 0.22728 |
| d.Placebo.Zinc | -3.5179 | 7.106 | 0.02512 | 0.06761 |
| sd.d | 4.6098 | 4.304 | 0.01522 | 0.16786 |
| B | -2.2594 | 5.945 | 0.02102 | 0.35531 |

Table S42, Meta-regression of AST versus Body Mass Index (BMI): Quantiles (2.5%, 25%, 50%, 75%, and 97.5%) for each variable across studies included in the analysis.

| Variable | 2.50% | 25% | 50% | 75% | 97.50% |
| --- | --- | --- | --- | --- | --- |
| d.Placebo.CBDR | -45.2886 | -32.1443 | -26.2526 | -20.43 | -7.6714 |
| d.Placebo.DHA_CHO_Vit_E | -29.8941 | -15.1602 | -7.7788 | -0.28368 | 14.9235 |
| d.Placebo.DHA_Vit_D | -33.6888 | -18.7962 | -11.2297 | -3.78494 | 12.1623 |
| d.Placebo.L_Carnitine | -15.1852 | -3.6717 | -0.8891 | 1.97698 | 14.6597 |
| d.Placebo.L_Citrulline | -44.0914 | -24.3465 | -14.5037 | -4.48377 | 16.3492 |
| d.Placebo.Losartan | -15.0528 | -0.3565 | 6.0894 | 12.12468 | 25.4535 |
| d.Placebo.Metformin | -12.1405 | -4.0996 | -2.0146 | -0.05095 | 6.4499 |
| d.Placebo.NAC | -38.5903 | -22.3438 | -13.7144 | -4.98058 | 11.8795 |
| d.Placebo.Omega_3 | -24.1943 | -12.6901 | -8.1246 | -3.52833 | 8.1937 |
| d.Placebo.Orlistat | -18.8552 | -7.455 | -4.7054 | -1.83896 | 10.8295 |
| d.Placebo.Vit_D | -27.6739 | -16.2867 | -13.4801 | -10.67214 | 0.3093 |
| d.Placebo.Vit_E | -13.6517 | -5.5435 | -3.0036 | -0.5589 | 6.8869 |
| d.Placebo.Vit_E_HXT | -36.418 | -23.9817 | -18.6083 | -12.67719 | 0.559 |
| d.Placebo.Vit_E_Vit_C | -29.4151 | -16.549 | -10.858 | -5.15691 | 8.7427 |
| d.Placebo.Zinc | -17.8657 | -6.9937 | -3.4952 | -0.08128 | 11.0072 |
| sd.d | 0.09509 | 1.4821 | 3.4486 | 6.40327 | 16.3467 |
| B | -13.226 | -5.6066 | -2.4545 | 0.76625 | 10.2886 |

Table S43, Meta-regression of AST versus Duration: Empirical mean and standard deviation for each variable, plus standard error of the mean, across studies included in the analysis.

| Variable | Mean | SD | Naive SE | Time-series SE |
| --- | --- | --- | --- | --- |
| d.Placebo.CBDR | -26.6049 | 9.282 | 0.03282 | 0.20966 |
| d.Placebo.DHA_CHO_Vit_E | -6.3321 | 10.599 | 0.03747 | 0.23673 |
| d.Placebo.DHA_Vit_D | -10.415 | 10.965 | 0.03877 | 0.24719 |
| d.Placebo.L_Carnitine | -0.1996 | 5.493 | 0.01942 | 0.10157 |
| d.Placebo.L_Citrulline | -16.7035 | 14.836 | 0.05245 | 0.43061 |
| d.Placebo.Losartan | 4.3543 | 9.357 | 0.03308 | 0.18594 |
| d.Placebo.Metformin | -2.9309 | 3.338 | 0.0118 | 0.04611 |
| d.Placebo.NAC | -13.9019 | 12.618 | 0.04461 | 0.33214 |
| d.Placebo.Omega_3 | -7.5838 | 6.981 | 0.02468 | 0.09366 |
| d.Placebo.Orlistat | -4.0481 | 5.542 | 0.0196 | 0.09672 |
| d.Placebo.Vit_D | -14.3451 | 5.54 | 0.01959 | 0.05105 |
| d.Placebo.Vit_E | -3.8022 | 3.552 | 0.01256 | 0.04446 |
| d.Placebo.Vit_E_HXT | -17.7065 | 8.645 | 0.03056 | 0.18305 |
| d.Placebo.Vit_E_Vit_C | -7.5118 | 10.396 | 0.03676 | 0.33851 |
| d.Placebo.Zinc | -3.632 | 6.183 | 0.02186 | 0.08817 |
| sd.d | 3.6004 | 3.454 | 0.01221 | 0.15221 |
| B | -1.6606 | 5.403 | 0.0191 | 0.23189 |

Table S44, Meta-regression of AST versus Duration: Quantiles (2.5%, 25%, 50%, 75%, and 97.5%) for each variable across studies included in the analysis.

| Variable | 2.50% | 25% | 50% | 75% | 97.50% |
| --- | --- | --- | --- | --- | --- |
| d.Placebo.CBDR | -44.4504 | -32.586 | -26.6434 | -20.6155 | -8.7784 |
| d.Placebo.DHA_CHO_Vit_E | -26.9564 | -13.062 | -6.3695 | 0.4997 | 14.6577 |
| d.Placebo.DHA_Vit_D | -31.8581 | -17.48 | -10.1978 | -3.2761 | 11.0172 |
| d.Placebo.L_Carnitine | -11.2539 | -2.52 | -0.3077 | 1.9574 | 11.5029 |
| d.Placebo.L_Citrulline | -45.4586 | -26.691 | -17.2132 | -6.5615 | 12.0181 |
| d.Placebo.Losartan | -13.8589 | -1.713 | 4.2383 | 10.4591 | 22.5909 |
| d.Placebo.Metformin | -9.91162 | -4.778 | -2.8338 | -1.0371 | 3.4131 |
| d.Placebo.NAC | -37.7182 | -22.514 | -13.9299 | -5.8102 | 11.3644 |
| d.Placebo.Omega_3 | -21.1892 | -11.651 | -7.5964 | -3.5024 | 6.2096 |
| d.Placebo.Orlistat | -15.1213 | -6.505 | -4.1487 | -1.7565 | 7.7433 |
| d.Placebo.Vit_D | -25.4676 | -16.867 | -14.3925 | -11.8605 | -2.9027 |
| d.Placebo.Vit_E | -10.9286 | -5.76 | -3.7522 | -1.8212 | 3.2799 |
| d.Placebo.Vit_E_HXT | -34.6332 | -23.182 | -17.7848 | -12.2174 | -0.8295 |
| d.Placebo.Vit_E_Vit_C | -28.3185 | -13.99 | -7.479 | -0.9416 | 13.0616 |
| d.Placebo.Zinc | -15.5777 | -6.81 | -3.781 | -0.5116 | 9.1414 |
| sd.d | 0.08971 | 1.151 | 2.6271 | 4.9172 | 12.9712 |
| B | -12.1363 | -5.046 | -1.7169 | 1.6258 | 9.4462 |

Table S45, Meta-regression of NAS versus Body Mass Index (BMI): Empirical mean and standard deviation for each variable, plus standard error of the mean, across studies included in the analysis.

| Variable | Mean | SD | Naive SE | Time-series SE |
| --- | --- | --- | --- | --- |
| d.Placebo.CBDR | 0.3609 | 1.578 | 0.005579 | 0.184073 |
| d.Placebo.Metformin | 0.1668 | 2.2393 | 0.007917 | 0.316301 |
| d.Placebo.Orlistat | -1.4553 | 2.5885 | 0.009152 | 0.424302 |
| d.Placebo.Vit_D | -1.246 | 1.6058 | 0.005677 | 0.208286 |
| d.Placebo.Vit_E | -0.5365 | 2.2413 | 0.007924 | 0.319239 |
| d.Placebo.Vit_E_Vit_C | -1.7862 | 3.3179 | 0.01173 | 0.549889 |
| sd.d | 0.8009 | 0.4676 | 0.001653 | 0.007947 |
| B | -1.3565 | 4.7739 | 0.016878 | 0.949626 |

Table S46, Meta-regression of NAS versus Body Mass Index (BMI): Quantiles (2.5%, 25%, 50%, 75%, and 97.5%) for each variable across studies included in the analysis.

| Variable | 2.50% | 25% | 50% | 75% | 97.50% |
| --- | --- | --- | --- | --- | --- |
| d.Placebo.CBDR | -2.29187 | -0.5838 | 0.1448 | 1.0553 | 4.322 |
| d.Placebo.Metformin | -3.23596 | -1.1387 | -0.2331 | 0.9519 | 6.164 |
| d.Placebo.Orlistat | -8.47702 | -2.2396 | -0.945 | 0.0317 | 2.329 |
| d.Placebo.Vit_D | -3.88022 | -2.1881 | -1.4895 | -0.5791 | 2.84 |
| d.Placebo.Vit_E | -3.93193 | -1.8706 | -0.9283 | 0.2535 | 5.47 |
| d.Placebo.Vit_E_Vit_C | -10.9058 | -2.7231 | -1.1147 | 0.1356 | 2.933 |
| sd.d | 0.04235 | 0.3938 | 0.7938 | 1.2043 | 1.579 |
| B | -14.8144 | -2.1178 | -0.2122 | 1.1633 | 5.133 |

Table S47, Meta-regression of NAS versus Duration: Empirical mean and standard deviation for each variable, plus standard error of the mean, across studies included in the analysis.

| Variable | Mean | SD | Naive SE | Time-series SE |
| --- | --- | --- | --- | --- |
| d.Placebo.CBDR | -0.002117 | 1.0025 | 0.003544 | 0.011079 |
| d.Placebo.Metformin | -0.407504 | 1.5675 | 0.005542 | 0.087694 |
| d.Placebo.Orlistat | -0.774843 | 1.8274 | 0.006461 | 0.141409 |
| d.Placebo.Vit_D | -1.617221 | 1.5044 | 0.005319 | 0.098867 |
| d.Placebo.Vit_E | -1.107623 | 1.565 | 0.005533 | 0.090357 |
| d.Placebo.Vit_E_Vit_C | -0.876317 | 1.6052 | 0.005675 | 0.089007 |
| sd.d | 0.803464 | 0.4685 | 0.001656 | 0.008338 |
| B | -0.010686 | 2.5939 | 0.009171 | 0.228677 |

Table S48, Meta-regression of NAS versus Duration: Quantiles (2.5%, 25%, 50%, 75%, and 97.5%) for each variable across studies included in the analysis.

| Variable | 2.50% | 25% | 50% | 75% | 97.50% |
| --- | --- | --- | --- | --- | --- |
| d.Placebo.CBDR | -2.12515 | -0.5289 | 0.001423 | 0.52792 | 2.118 |
| d.Placebo.Metformin | -3.63543 | -1.3233 | -0.419516 | 0.50189 | 2.814 |
| d.Placebo.Orlistat | -4.5741 | -1.7937 | -0.756149 | 0.24453 | 3.042 |
| d.Placebo.Vit_D | -4.75595 | -2.4693 | -1.607357 | -0.76474 | 1.484 |
| d.Placebo.Vit_E | -4.32889 | -2.0214 | -1.114287 | -0.19252 | 2.119 |
| d.Placebo.Vit_E_Vit_C | -4.15182 | -1.8541 | -0.882595 | 0.07752 | 2.45 |
| sd.d | 0.03896 | 0.3936 | 0.803334 | 1.20877 | 1.579 |
| B | -5.62795 | -1.2743 | 0.043809 | 1.26244 | 5.878 |

Table S49, Meta-regression of Baseline ALT versus NAS: Empirical mean and standard deviation for each variable, plus standard error of the mean, across studies included in the analysis.

| Variable | Mean | SD | Naive SE | Time-series SE |
| --- | --- | --- | --- | --- |
| d.Placebo.CBDR | -0.4046 | 1.8954 | 0.006701 | 0.17115 |
| d.Placebo.Metformin | -0.8094 | 1.9643 | 0.006945 | 0.170151 |
| d.Placebo.Orlistat | -0.5046 | 1.4407 | 0.005093 | 0.107835 |
| d.Placebo.Vit_D | -1.419 | 1.2309 | 0.004352 | 0.071653 |
| d.Placebo.Vit_E | -1.5087 | 1.9553 | 0.006913 | 0.170956 |
| d.Placebo.Vit_E_Vit_C | -0.5319 | 1.7889 | 0.006325 | 0.137872 |
| sd.d | 0.8032 | 0.4661 | 0.001648 | 0.008178 |
| B | 0.7513 | 3.0717 | 0.01086 | 0.330218 |

Table S50, Meta-regression of Baseline ALT versus NAS: Quantiles (2.5%, 25%, 50%, 75%, and 97.5%) for each variable across studies included in the analysis.

| Variable | 2.50% | 25% | 50% | 75% | 97.50% |
| --- | --- | --- | --- | --- | --- |
| d.Placebo.CBDR | -5.00754 | -1.2795 | -0.2051 | 0.7313 | 2.819 |
| d.Placebo.Metformin | -5.48096 | -1.7299 | -0.6148 | 0.3841 | 2.565 |
| d.Placebo.Orlistat | -3.10002 | -1.3577 | -0.6285 | 0.2021 | 2.871 |
| d.Placebo.Vit_D | -3.78224 | -2.1284 | -1.4949 | -0.7853 | 1.345 |
| d.Placebo.Vit_E | -6.27149 | -2.4276 | -1.3021 | -0.3116 | 1.862 |
| d.Placebo.Vit_E_Vit_C | -3.66146 | -1.634 | -0.6978 | 0.3512 | 3.676 |
| sd.d | 0.04653 | 0.3951 | 0.8005 | 1.2049 | 1.576 |
| B | -4.19767 | -0.9376 | 0.2664 | 1.7527 | 8.974 |

Table S51, Meta-regression of Baseline AST versus NAS: Empirical mean and standard deviation for each variable, plus standard error of the mean, across studies included in the analysis.

| Variable | Mean | SD | Naive SE | Time-series SE |
| --- | --- | --- | --- | --- |
| d.Placebo.CBDR | -0.4046 | 1.8954 | 0.006701 | 0.17115 |
| d.Placebo.Metformin | -0.8094 | 1.9643 | 0.006945 | 0.170151 |
| d.Placebo.Orlistat | -0.5046 | 1.4407 | 0.005093 | 0.107835 |
| d.Placebo.Vit_D | -1.419 | 1.2309 | 0.004352 | 0.071653 |
| d.Placebo.Vit_E | -1.5087 | 1.9553 | 0.006913 | 0.170956 |
| d.Placebo.Vit_E_Vit_C | -0.5319 | 1.7889 | 0.006325 | 0.137872 |
| sd.d | 0.8032 | 0.4661 | 0.001648 | 0.008178 |
| B | 0.7513 | 3.0717 | 0.01086 | 0.330218 |

Table S52, Meta-regression of Baseline AST versus NAS: Quantiles (2.5%, 25%, 50%, 75%, and 97.5%) for each variable across studies included in the analysis.

| Variable | 2.50% | 25% | 50% | 75% | 97.50% |
| --- | --- | --- | --- | --- | --- |
| d.Placebo.CBDR | -9.80122 | -1.3573 | -0.1723 | 0.8351 | 3.004 |
| d.Placebo.Metformin | -10.35846 | -1.8244 | -0.5871 | 0.4733 | 2.698 |
| d.Placebo.Orlistat | -3.32621 | -1.4599 | -0.6235 | 0.3396 | 6.452 |
| d.Placebo.Vit_D | -3.876 | -2.2074 | -1.4955 | -0.6715 | 3.853 |
| d.Placebo.Vit_E | -11.20158 | -2.5303 | -1.2973 | -0.2322 | 2.017 |
| d.Placebo.Vit_E_Vit_C | -3.61033 | -1.6671 | -0.7078 | 0.3629 | 6.471 |
| sd.d | 0.03468 | 0.3647 | 0.7697 | 1.1846 | 1.574 |
| B | -4.50315 | -1.1864 | 0.2288 | 1.9165 | 18.142 |

Figures


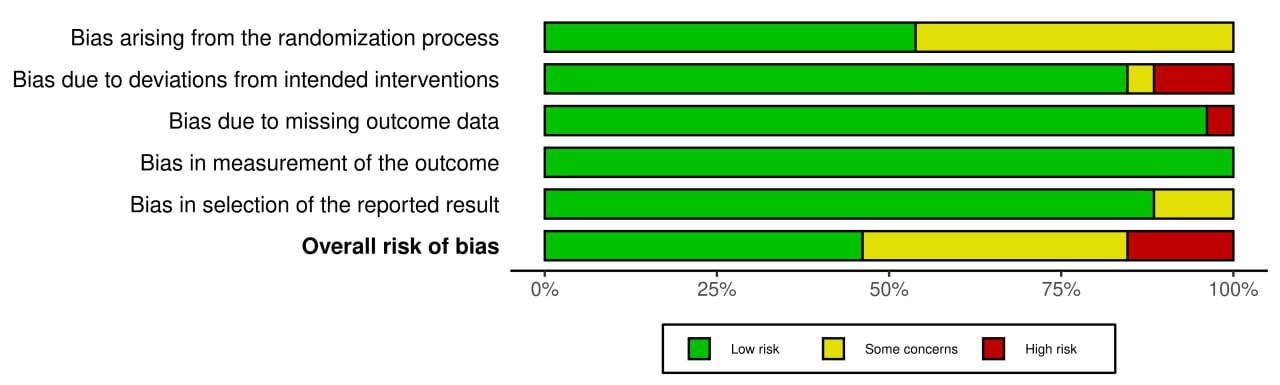


Figure S1, Overall risk of bias assessment done for the clinical trial studies based on the RoB2 tool


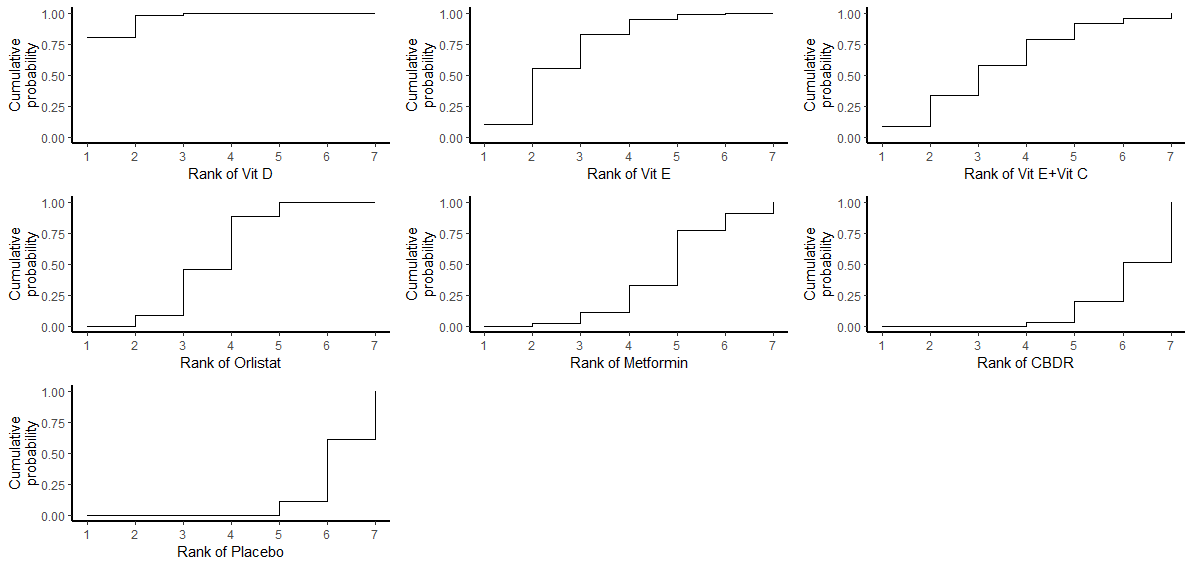


Figure SS2, SUCRA plots showing the cumulative probability of each intervention being among the best for reducing NAS. Higher curves indicate better rankings.


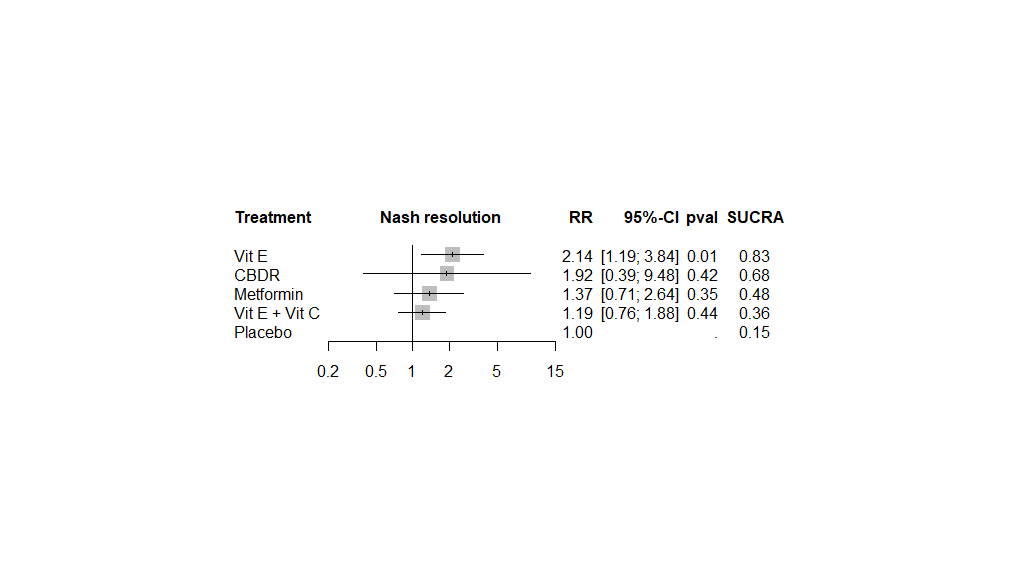


Figure S3, Forest plots for network meta-analysis of NASH resolution events.


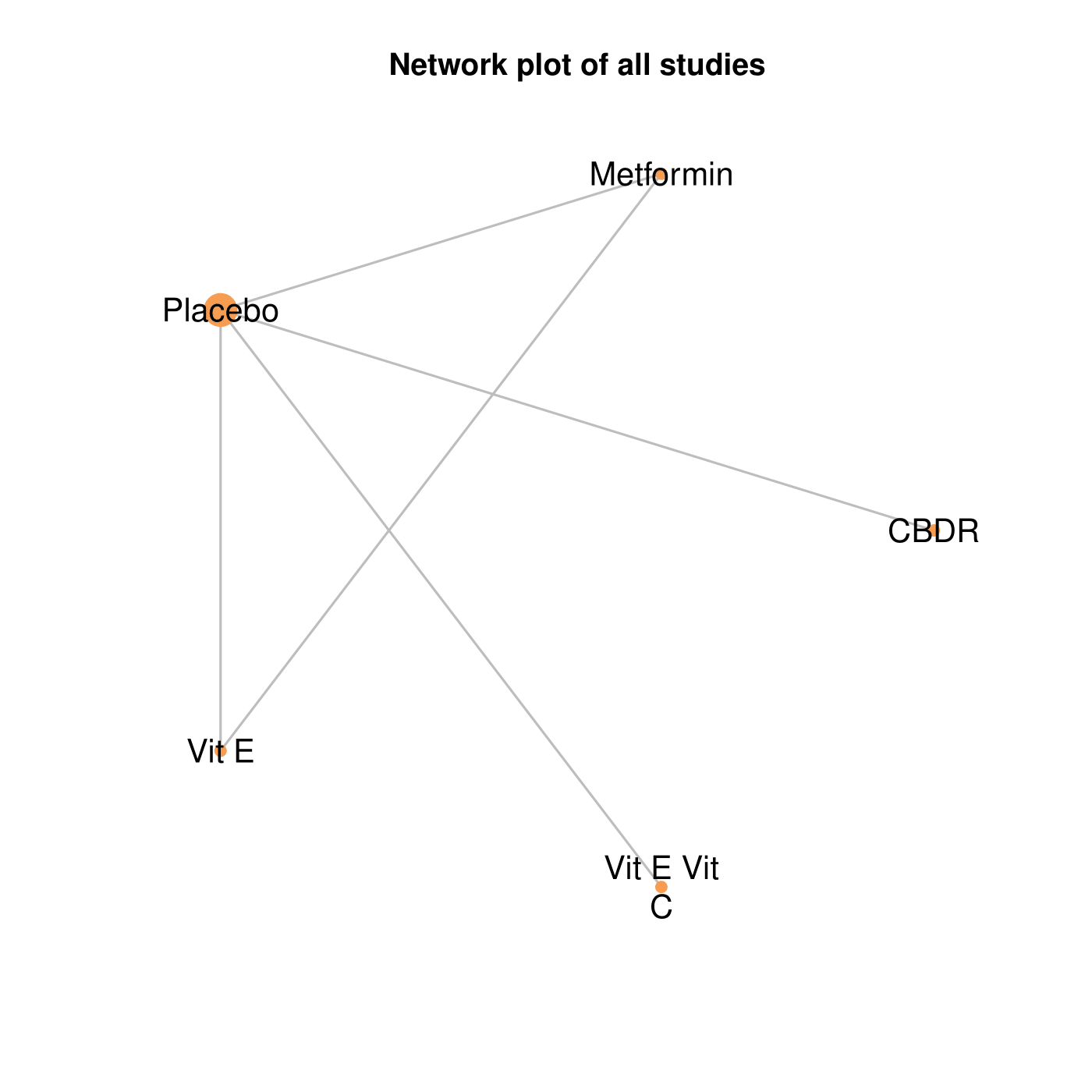


Figure S4, Network geometry of NASH resolution events for all included studies, by drug treatments.


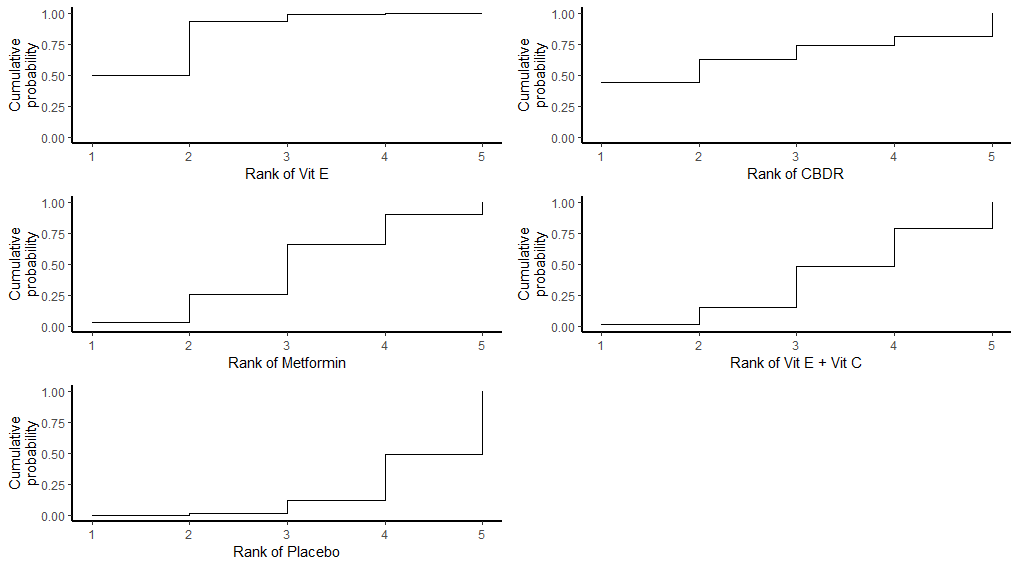


Figure S5, SUCRA plots showing the cumulative probability of each intervention being among the best for reducing NASH resolution events. Higher curves indicate better rankings.


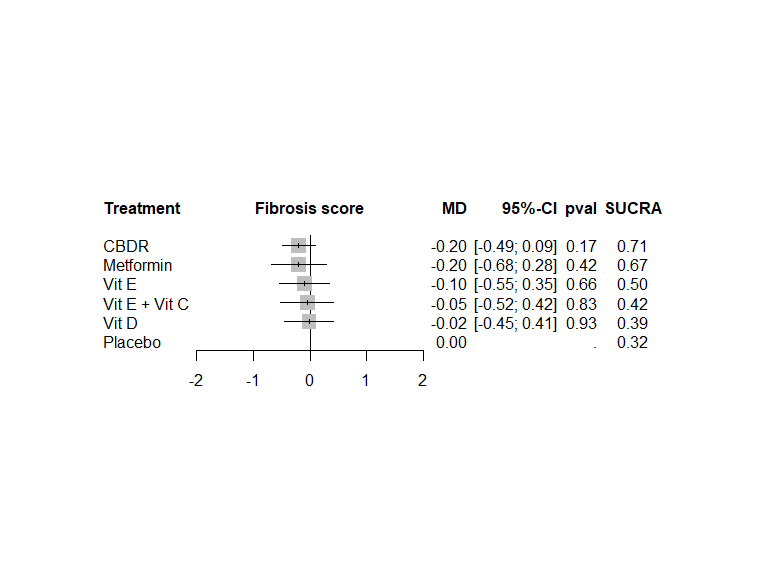


Figure S6, Forest plots for network meta-analysis of fibrosis improvement score.


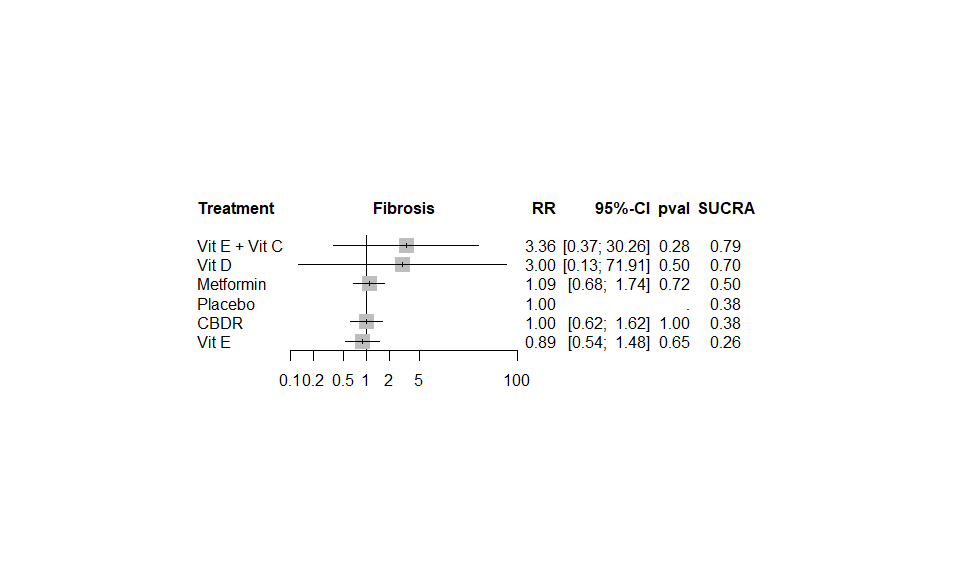
Figure S7, Forest plots for network meta-analysis of fibrosis improvement events.


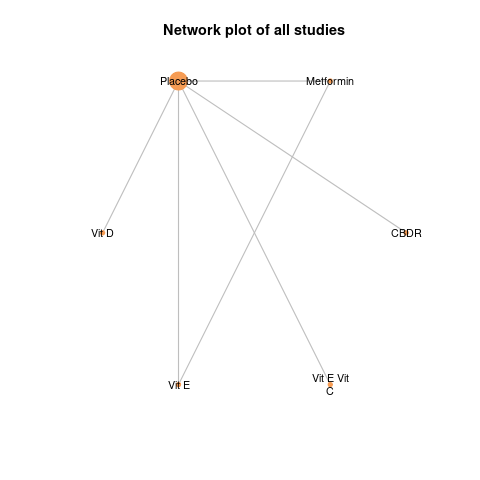


Figure S8, Network geometry of fibrosis improvement score and events for all included studies, by drug treatments.


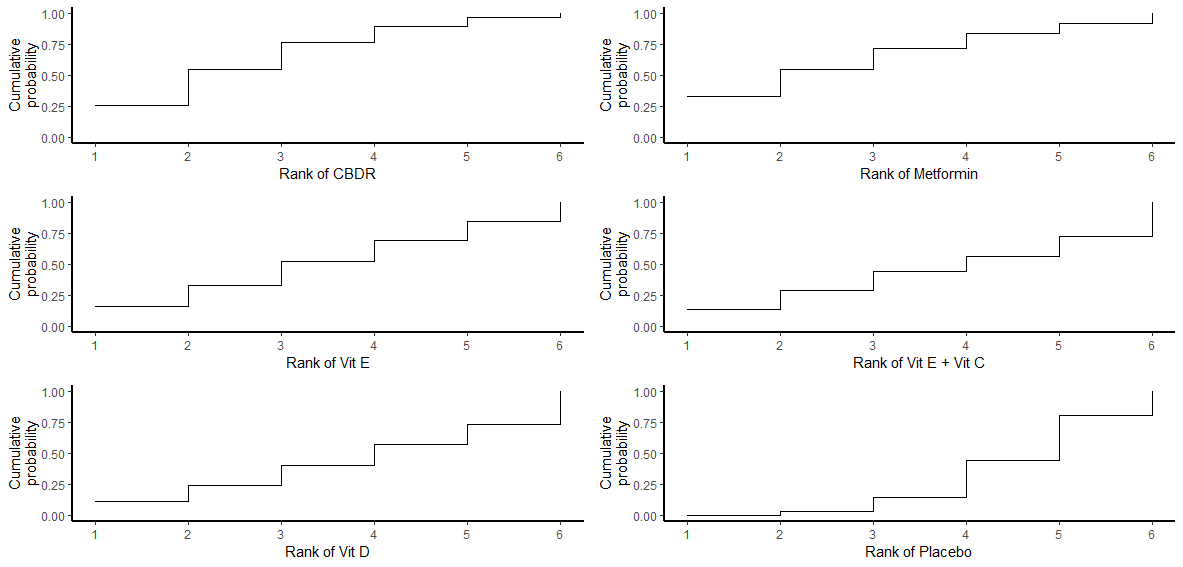


Figure S9, SUCRA plots showing the cumulative probability of each intervention being among the best for reducing fibrosis improvement score. Higher curves indicate better rankings.


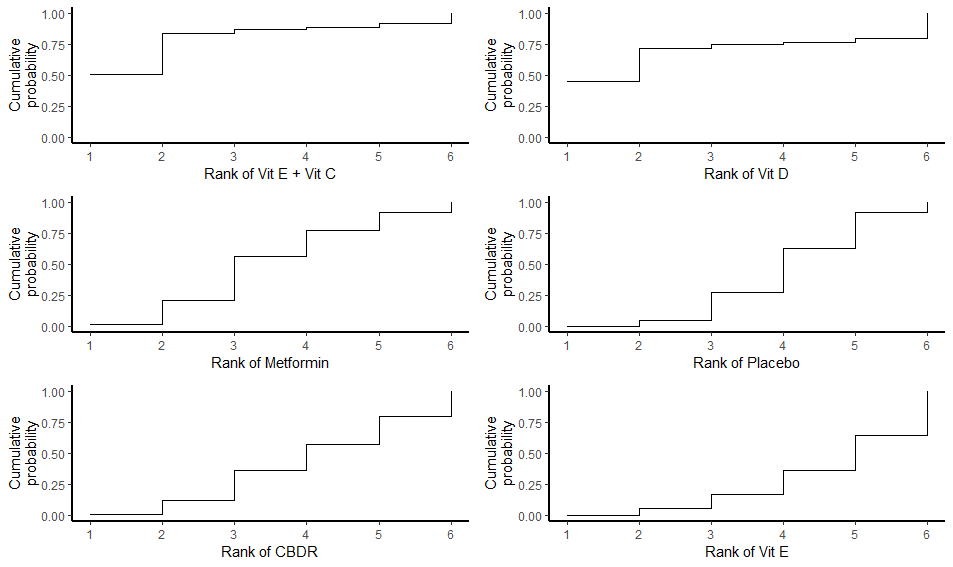


Figure S10, SUCRA plots showing the cumulative probability of each intervention being among the best for reducing fibrosis improvement events. Higher curves indicate better rankings.


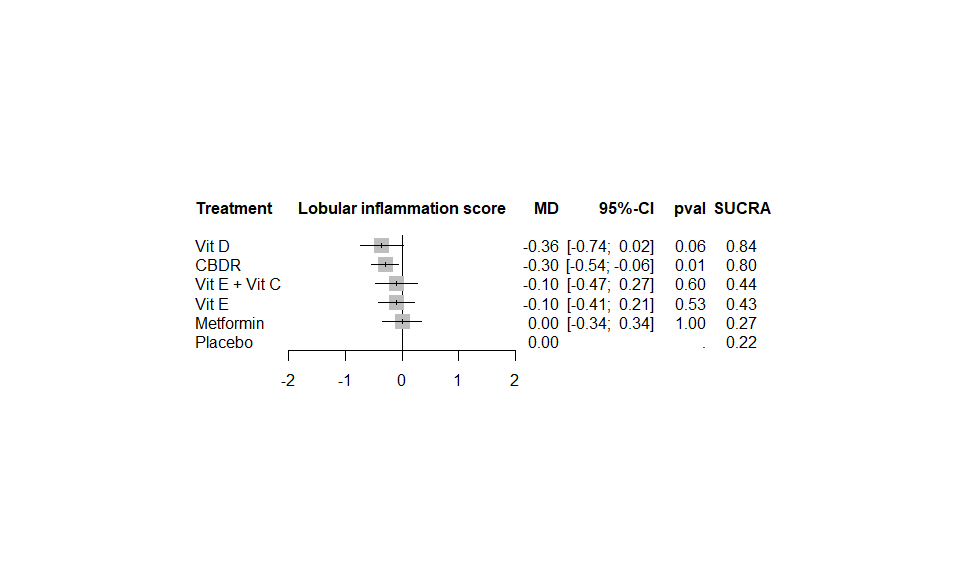


Figure S11, Forest plots for network meta-analysis of Lobular inflammation improvement score.


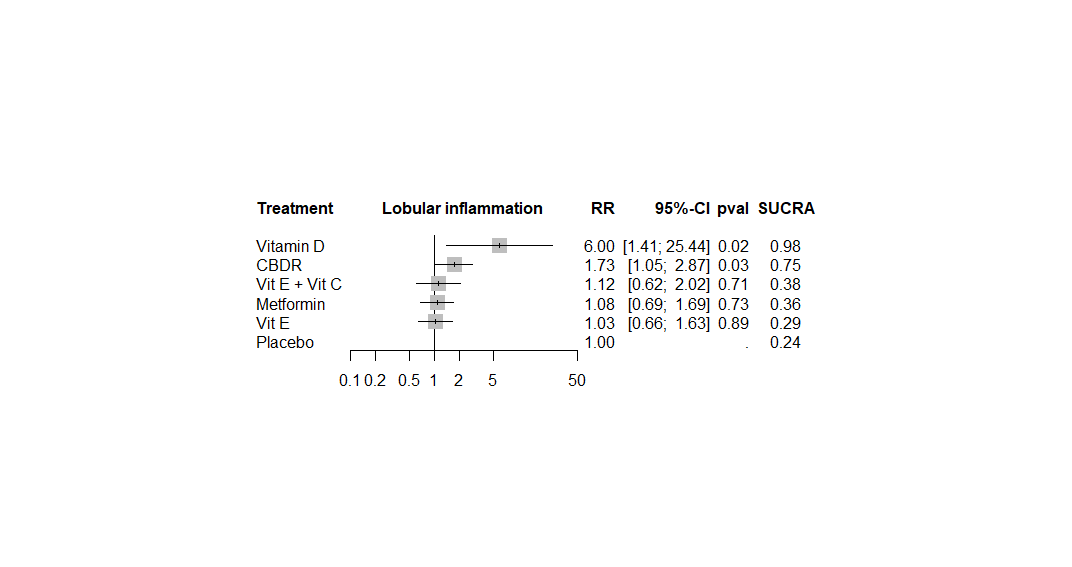


Figure S12, Forest plots for network meta-analysis of Lobular inflammation improvement events.


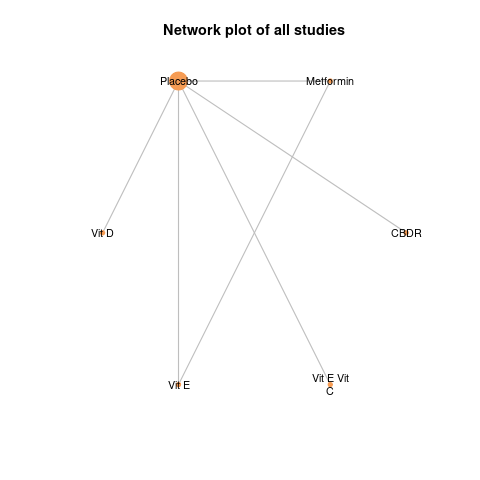


Figure S13, Network geometry of Lobular improvement score and events for all included studies, by drug treatments.


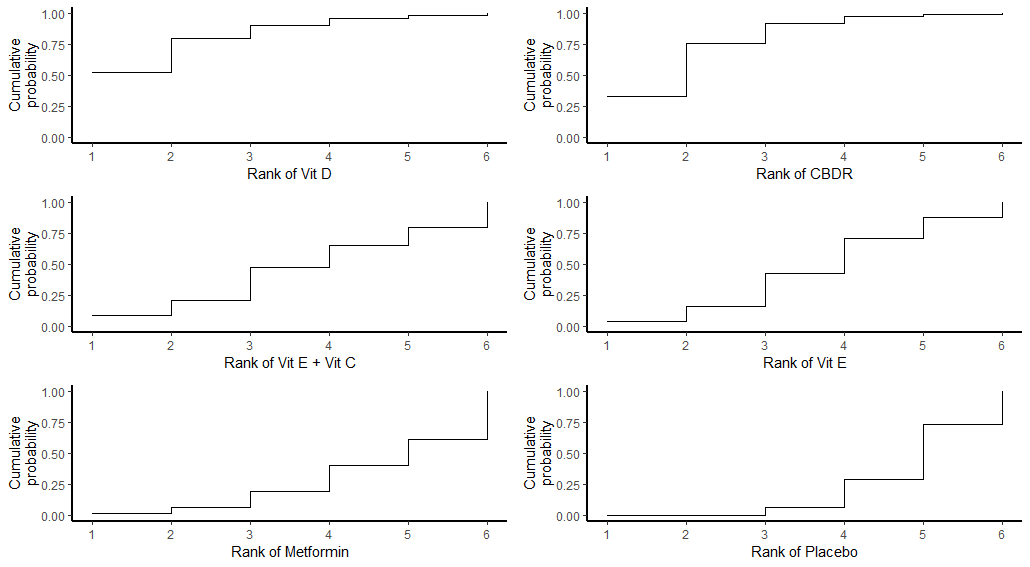


Figure S14, SUCRA plots showing the cumulative probability of each intervention being among the best for reducing Lobular inflammation improvement score. Higher curves indicate better rankings.


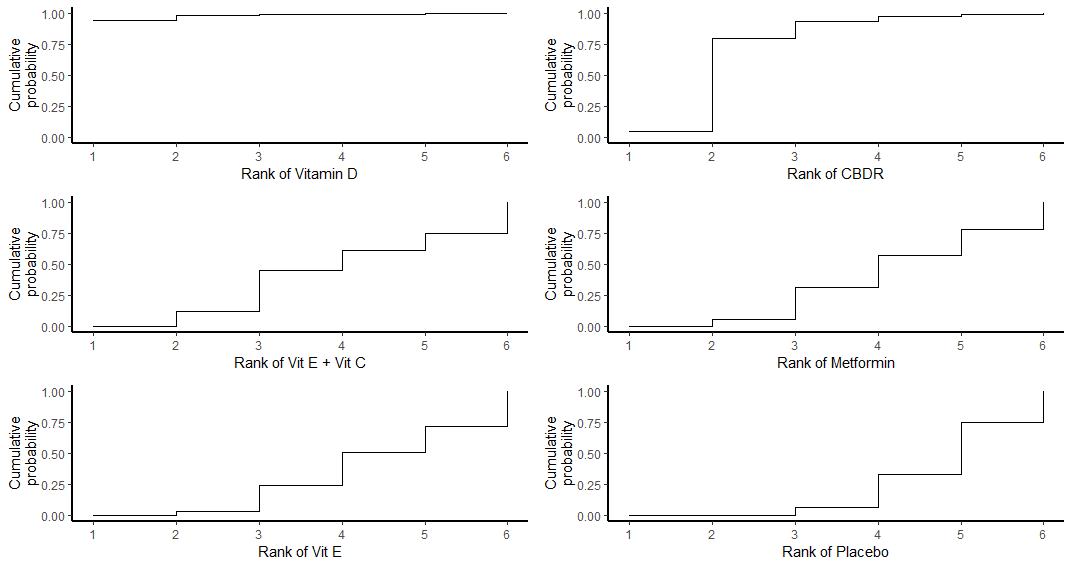


Figure S15, SUCRA plots showing the cumulative probability of each intervention being among the best for reducing Lobular inflammation improvement events. Higher curves indicate better rankings.


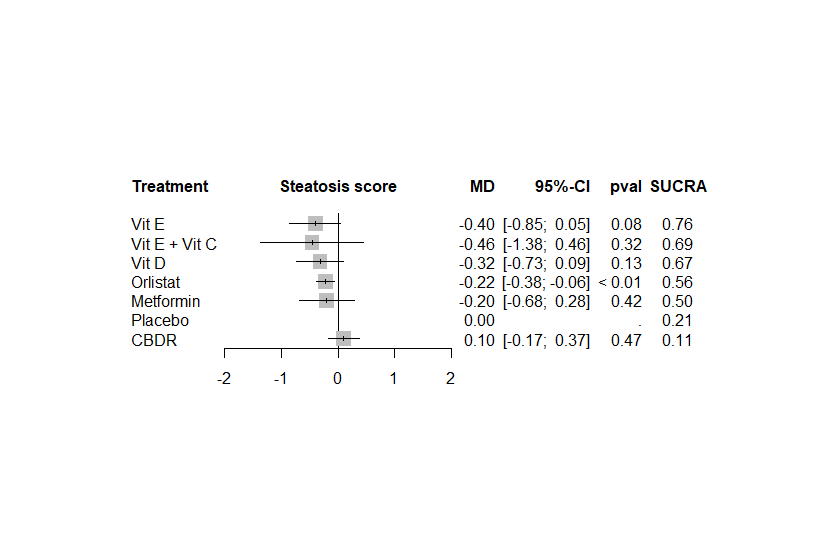


Figure S16, Forest plots for network meta-analysis of Steatosis improvement score.


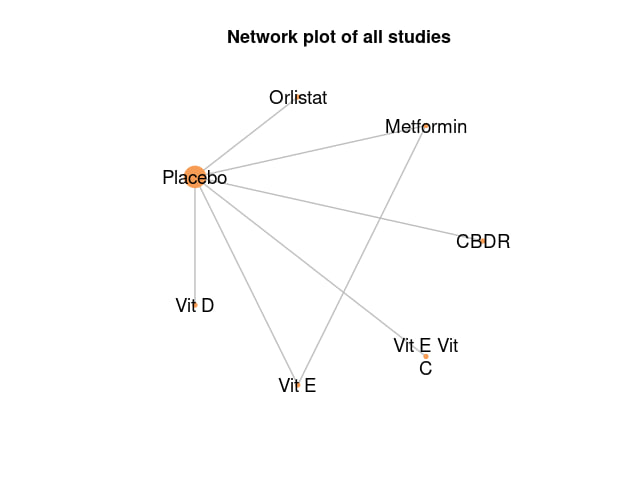


Figure S17, Network geometry of Steatosis improvement score for all included studies, by drug treatments.


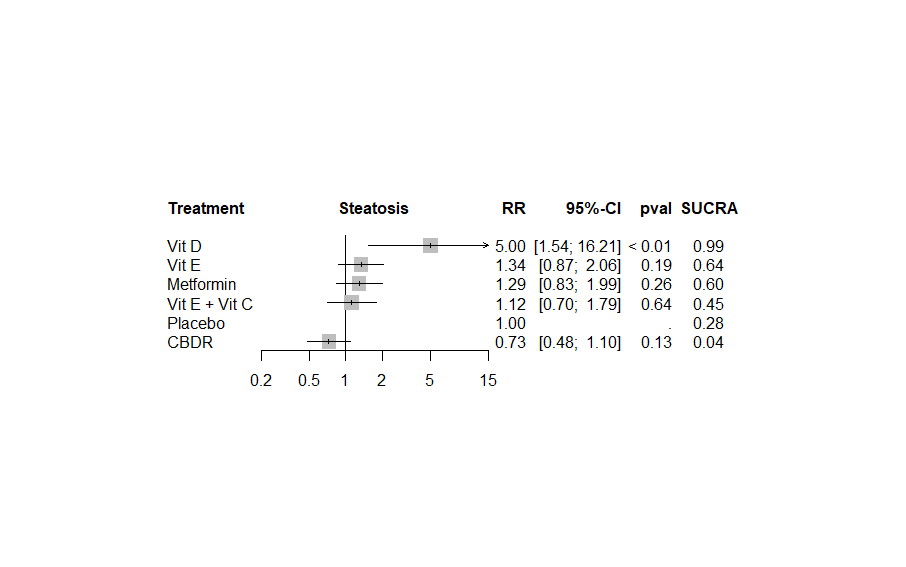
Figure S18, Forest plots for network meta-analysis of Steatosis improvement events.


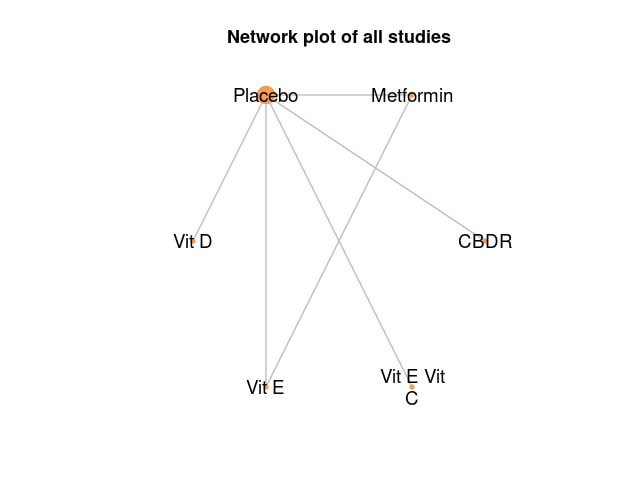


Figure S19, Network geometry of Steatosis improvement events for all included studies, by drug treatments.


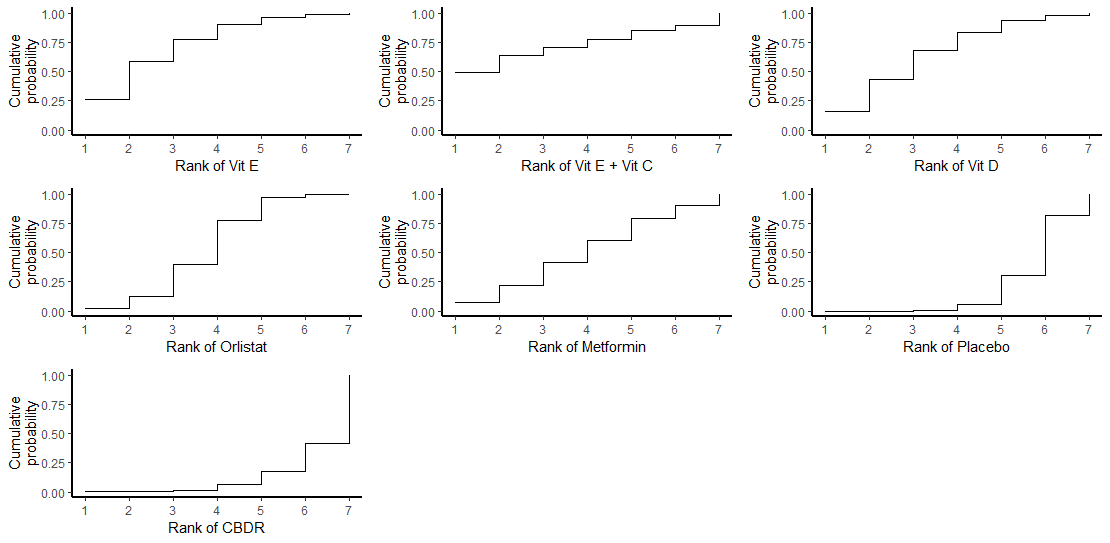


Figure S20, SUCRA plots showing the cumulative probability of each intervention being among the best for reducing Steatosis improvement score. Higher curves indicate better rankings.


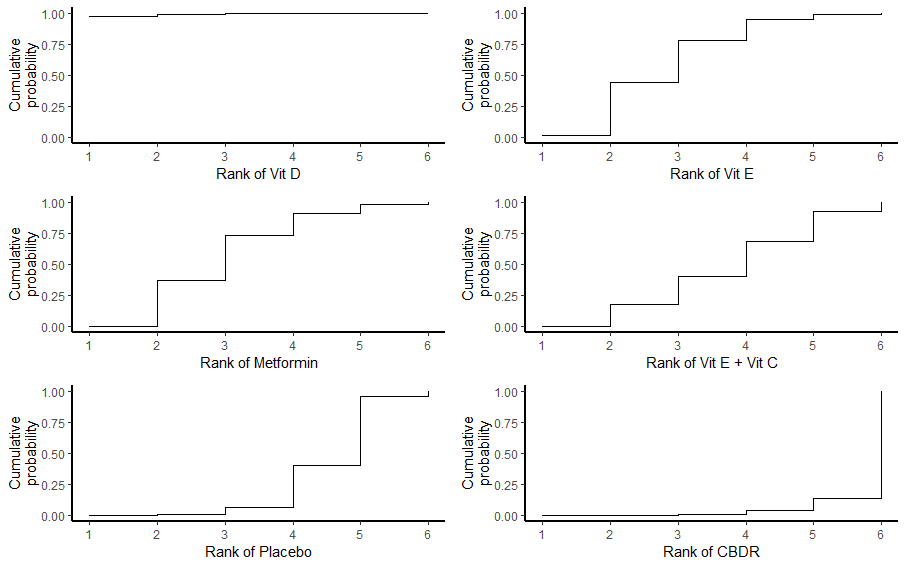


Figure S21, SUCRA plots showing the cumulative probability of each intervention being among the best for reducing Steatosis improvement event. Higher curves indicate better rankings.


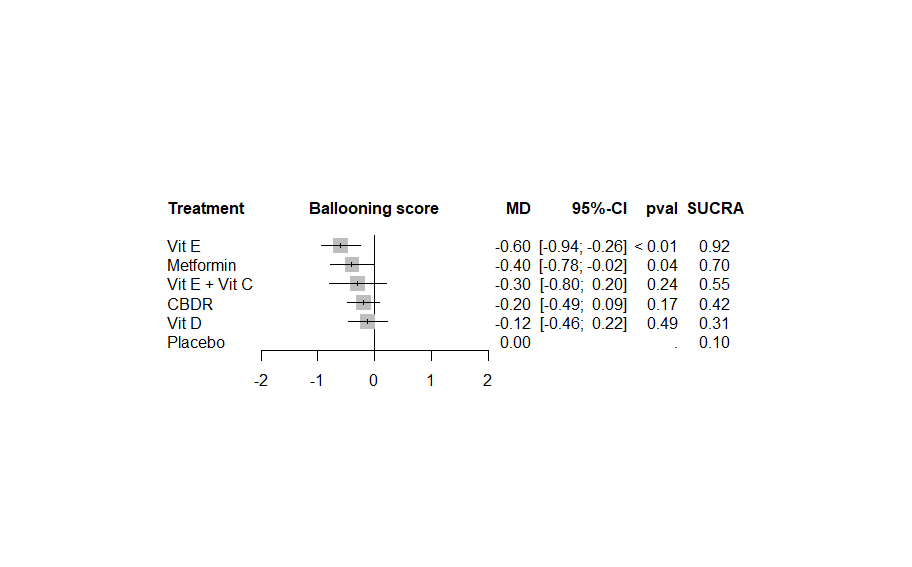


Figure S22, Forest plots for network meta-analysis of Ballooning improvement score.


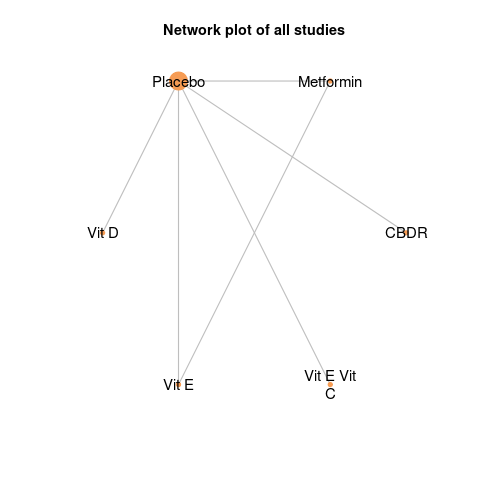


Figure S23, Network geometry of Ballooning improvement score for all included studies, by drug treatments.


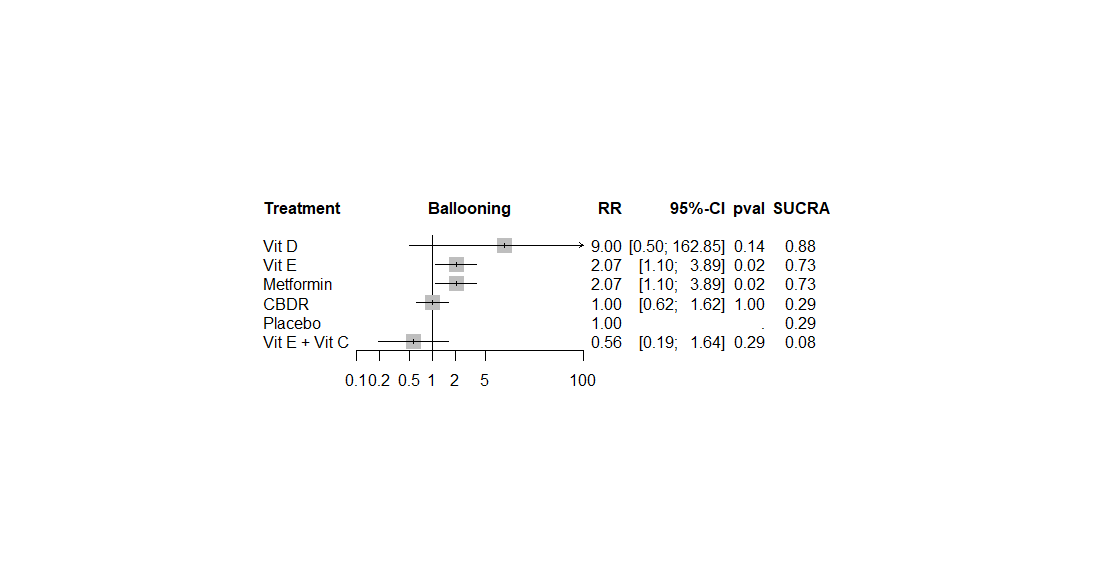


Figure S24, Forest plots for network meta-analysis of Ballooning improvement events.


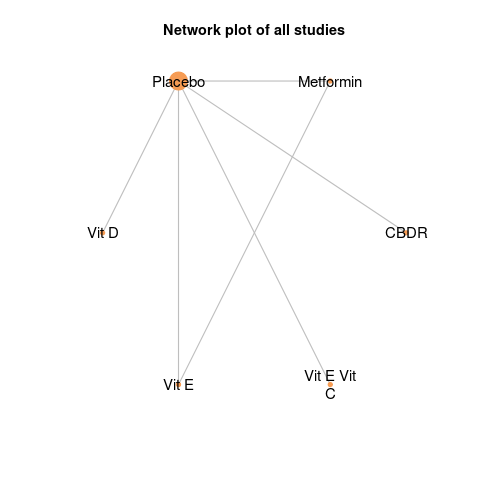


Figure S25, Network geometry of Ballooning improvement events for all included studies, by drug treatments.


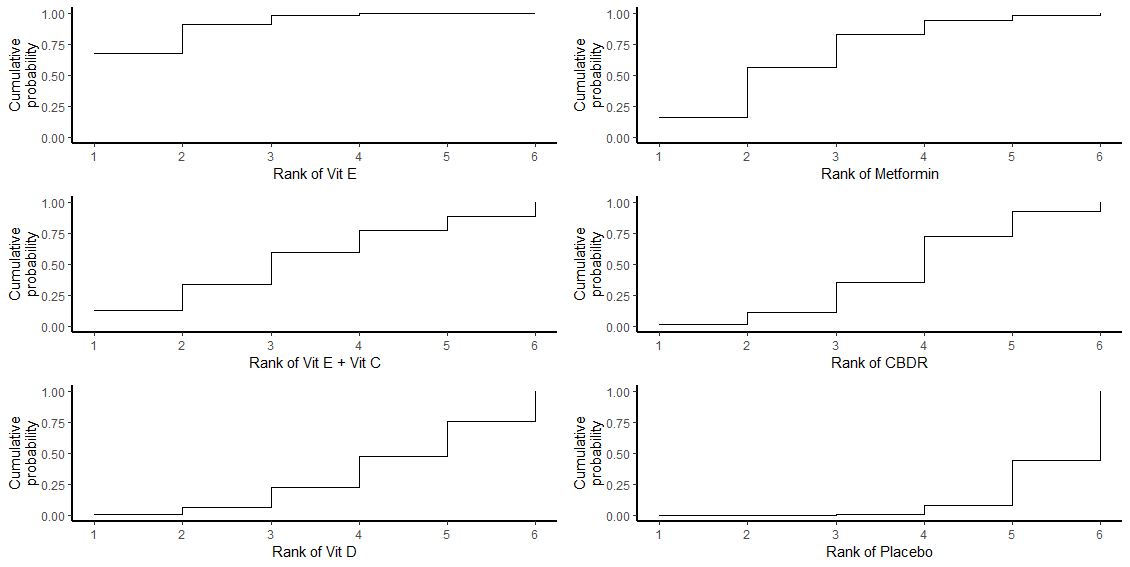


Figure S26, SUCRA plots showing the cumulative probability of each intervention being among the best for reducing Ballooning improvement score. Higher curves indicate better rankings.


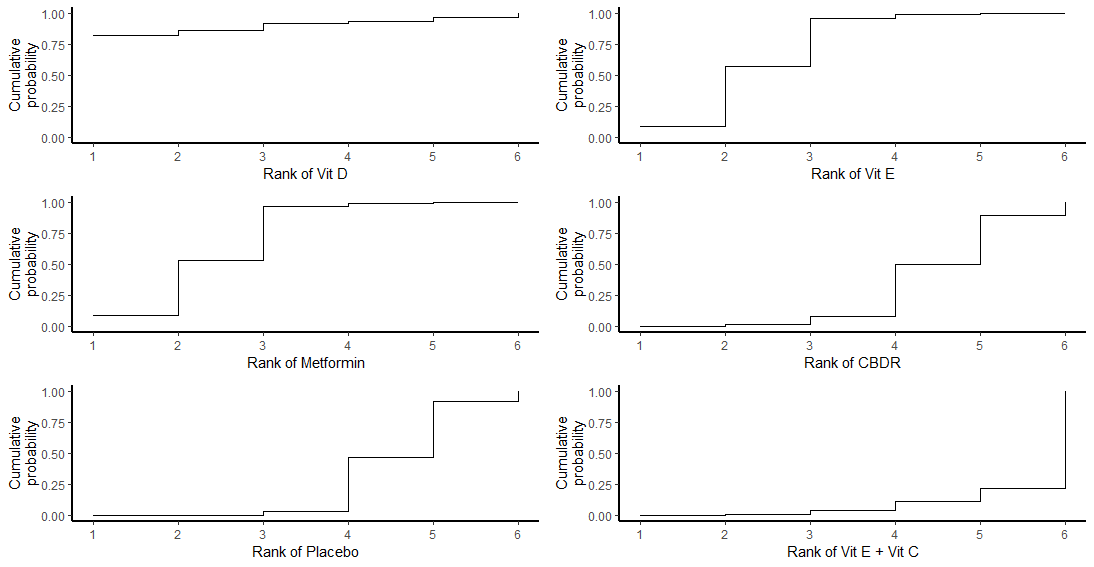


Figure S27, SUCRA plots showing the cumulative probability of each intervention being among the best for reducing Ballooning improvement event. Higher curves indicate better rankings.


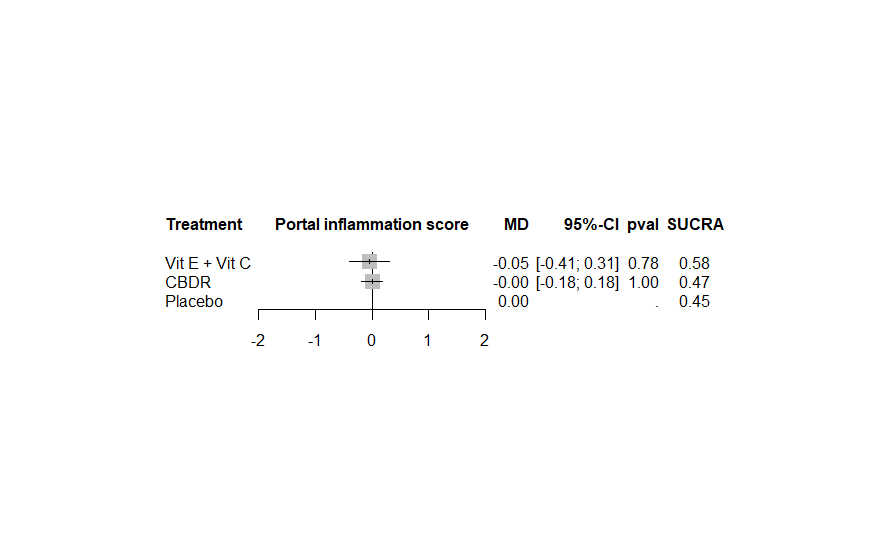
Figure S28, Forest plots for network meta-analysis of Portal inflammation improvement score.


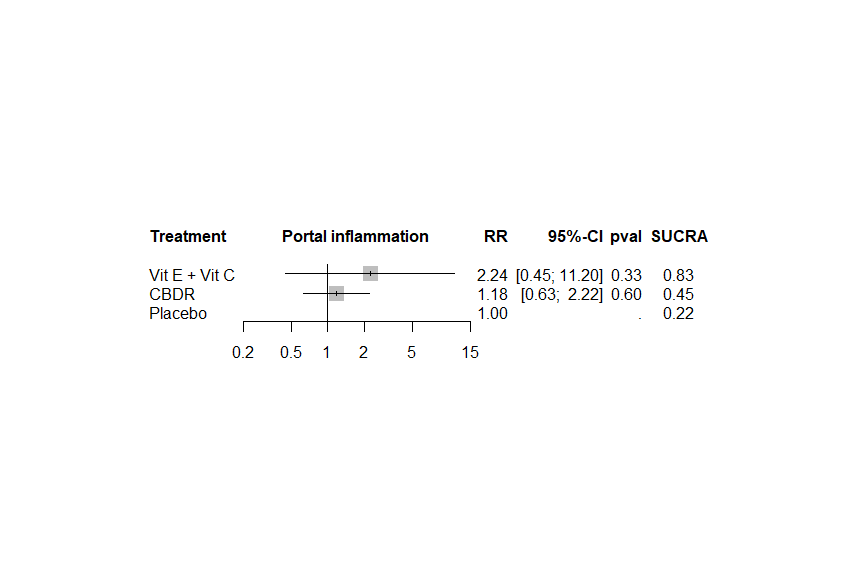


Figure S29, Forest plots for network meta-analysis of Portal inflammation improvement events.


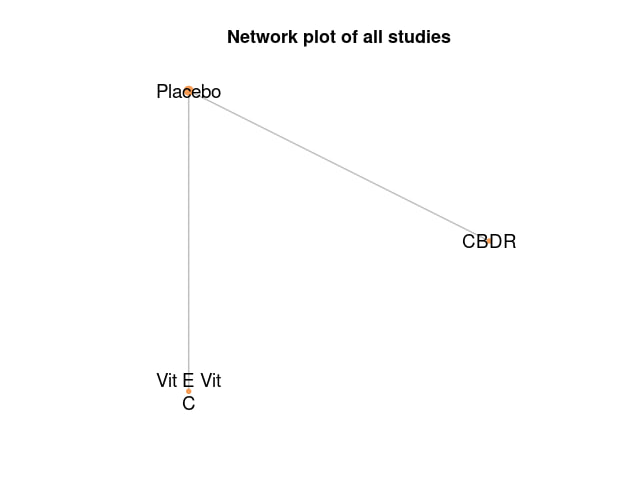


Figure S30, Network geometry of Network geometry of Portal inflammation improvement score and events for all included studies, by drug treatments. for all included studies, by drug treatments.


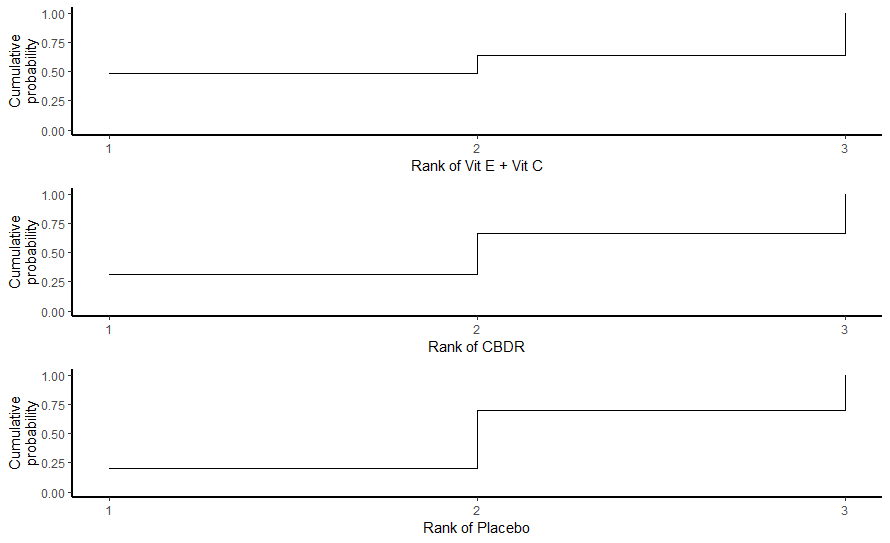


Figure S31, SUCRA plots showing the cumulative probability of each intervention being among the best for reducing Portal inflammation improvement score. Higher curves indicate better rankings.


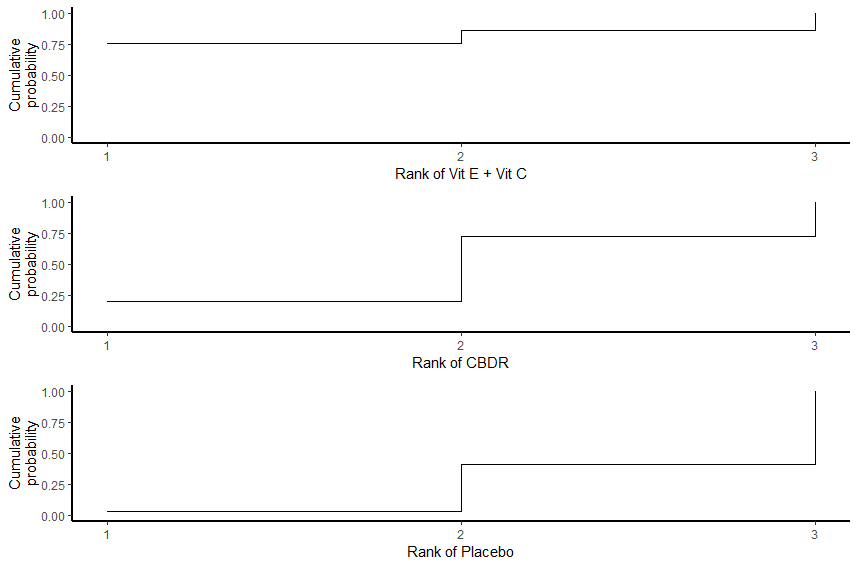


Figure S32, SUCRA plots showing the cumulative probability of each intervention being among the best for reducing Portal inflammation improvement events. Higher curves indicate better rankings.


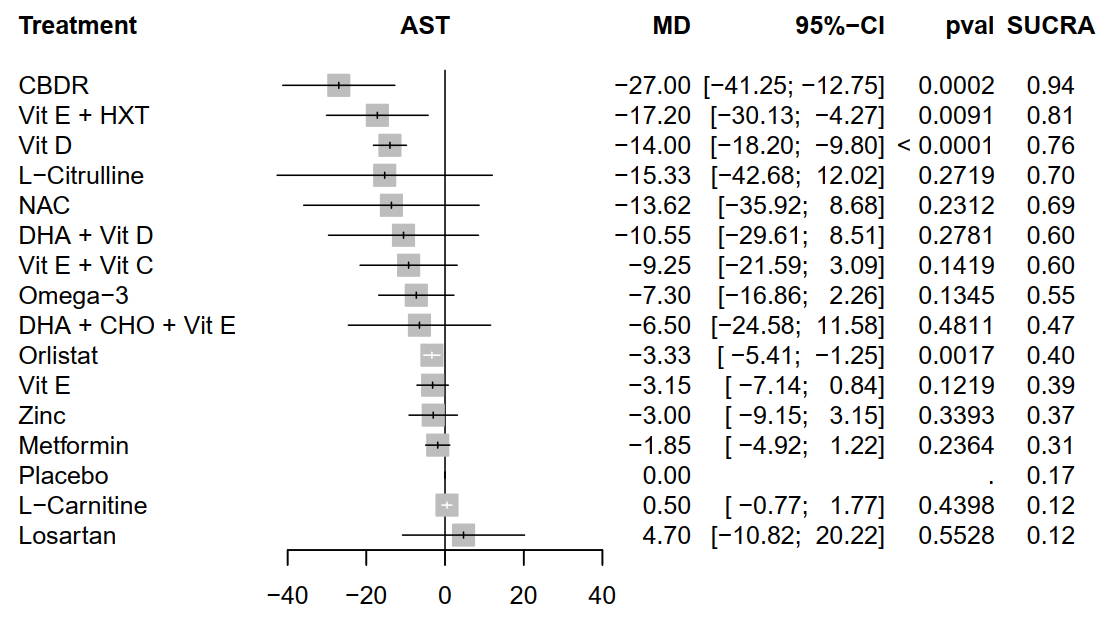


Figure S33, Forest plots for network meta-analysis of AST after sensitivity analysis.


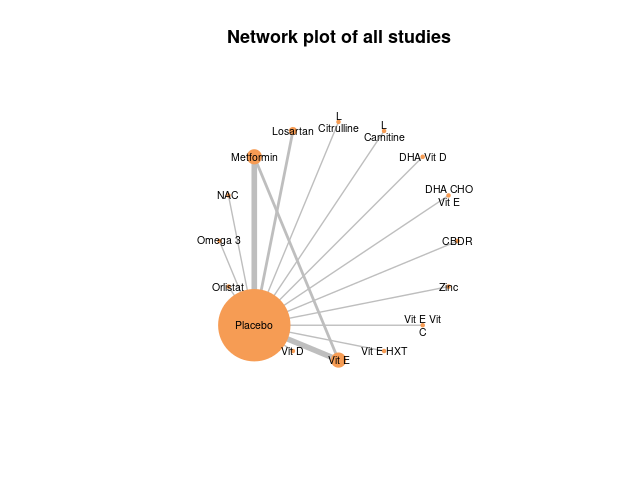


Figure S34, Network geometry of AST after sensitivity analysis for all included studies, by drug treatments.


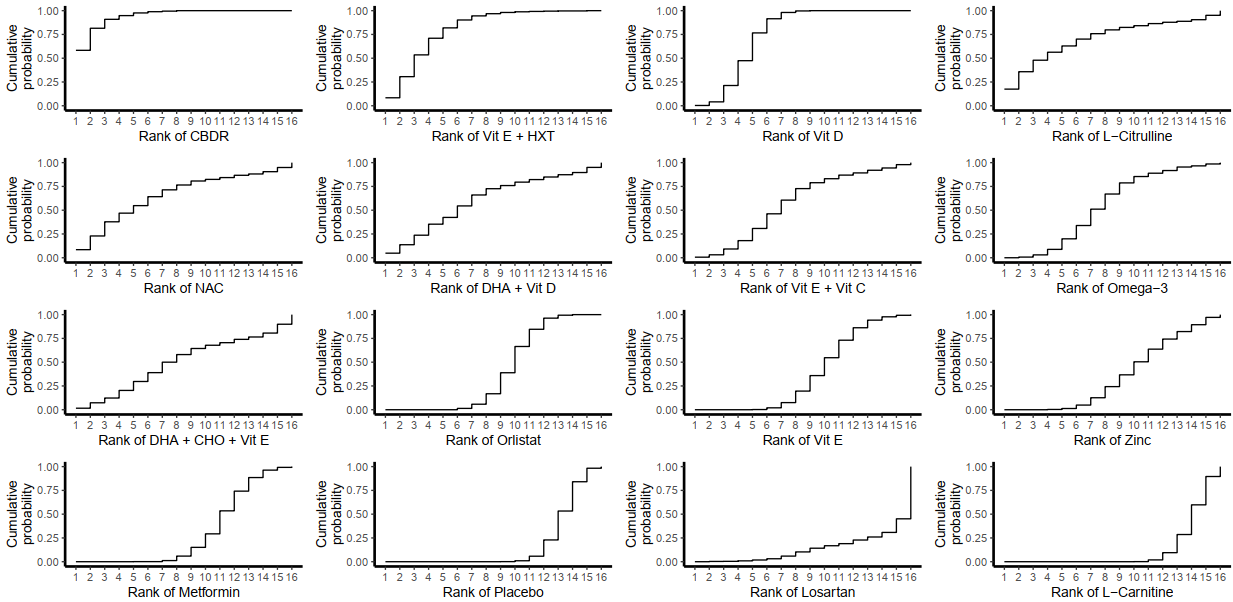


*Figure S35,* *SUCRA plots showing the cumulative probability of each intervention being among the best for reducing AST after sensitivity analysis. Higher curves indicate better rankings.*


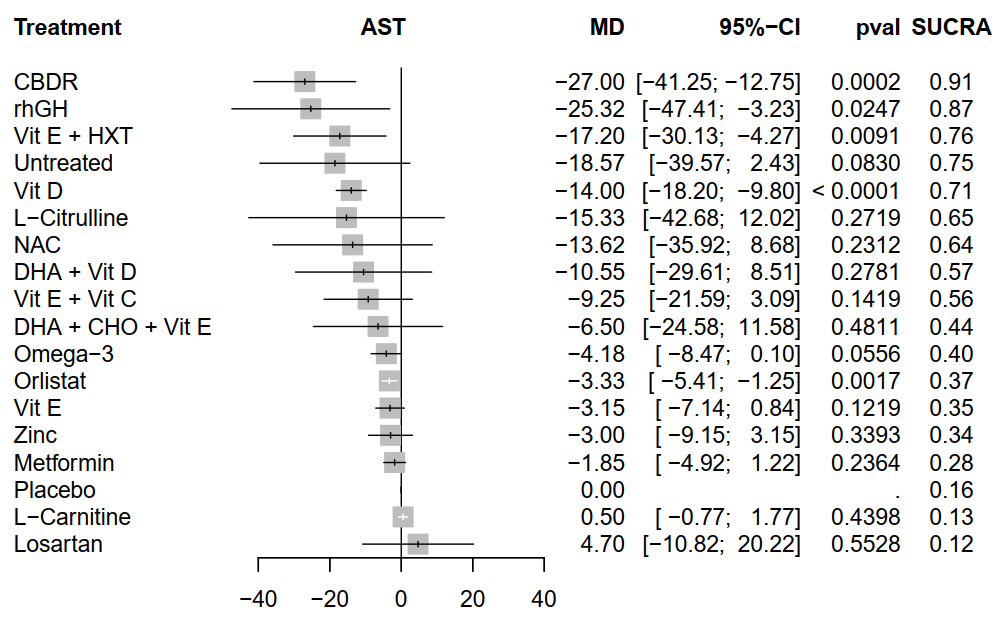


Figure S36, Forest plots for network meta-analysis of AST before sensitivity analysis.


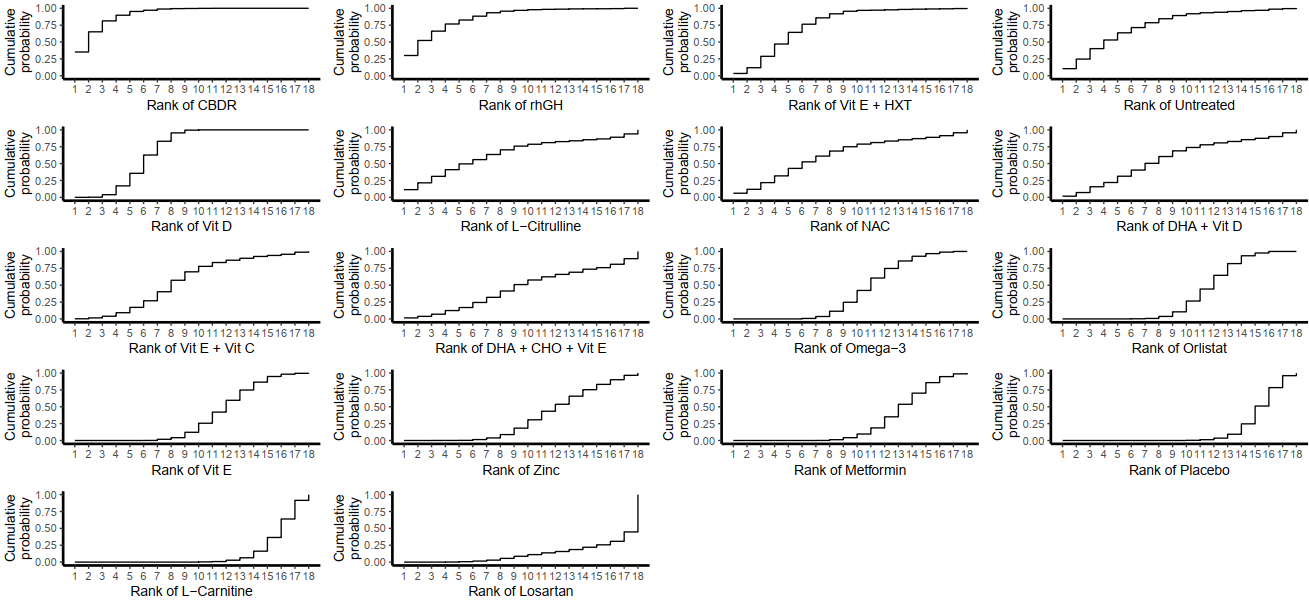


Figure S37, SUCRA plots showing the cumulative probability of each intervention being among the best for reducing AST before sensitivity analysis. Higher curves indicate better rankings.


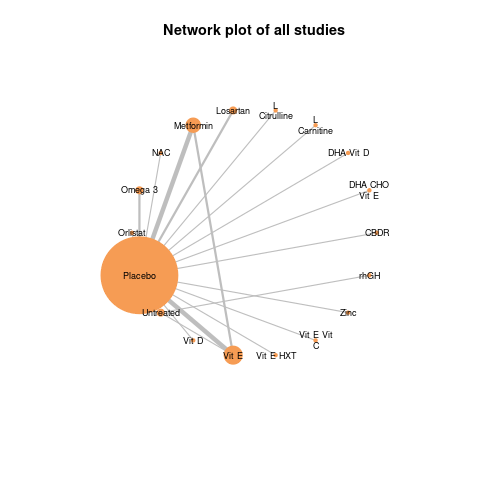


Figure S38, Network geometry of AST before sensitivity analysis for all included studies, by drug treatments.


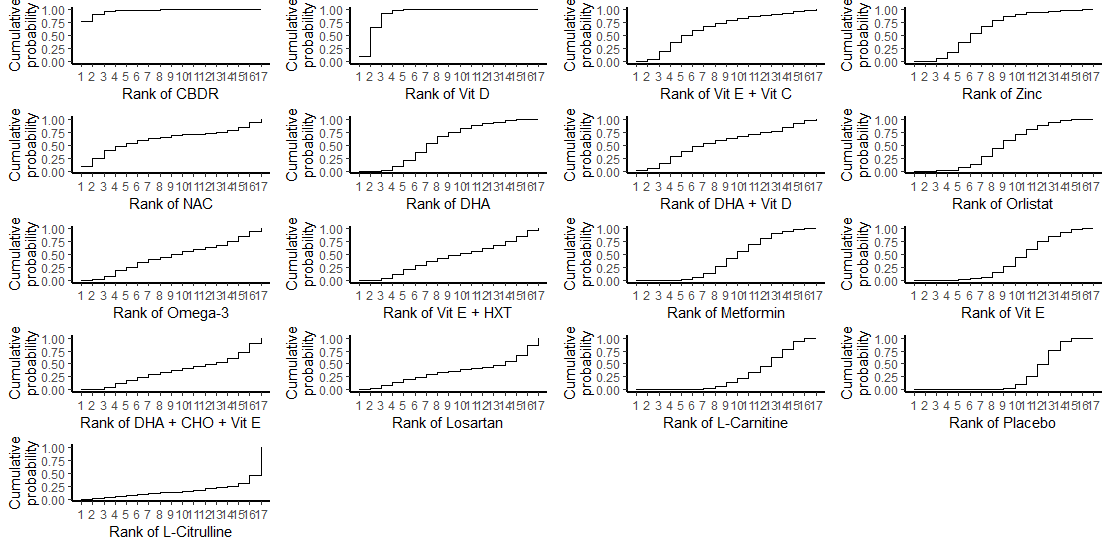


Figure S39, SUCRA plots showing the cumulative probability of each intervention being among the best for reducing ALT after sensitivity analysis. Higher curves indicate better rankings.


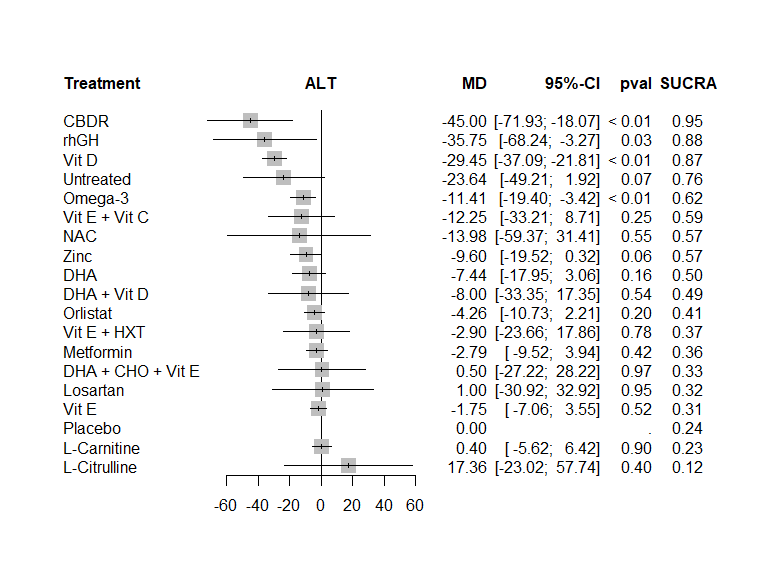


Figure S40, Forest plots for network meta-analysis of ALT before sensitivity analysis.


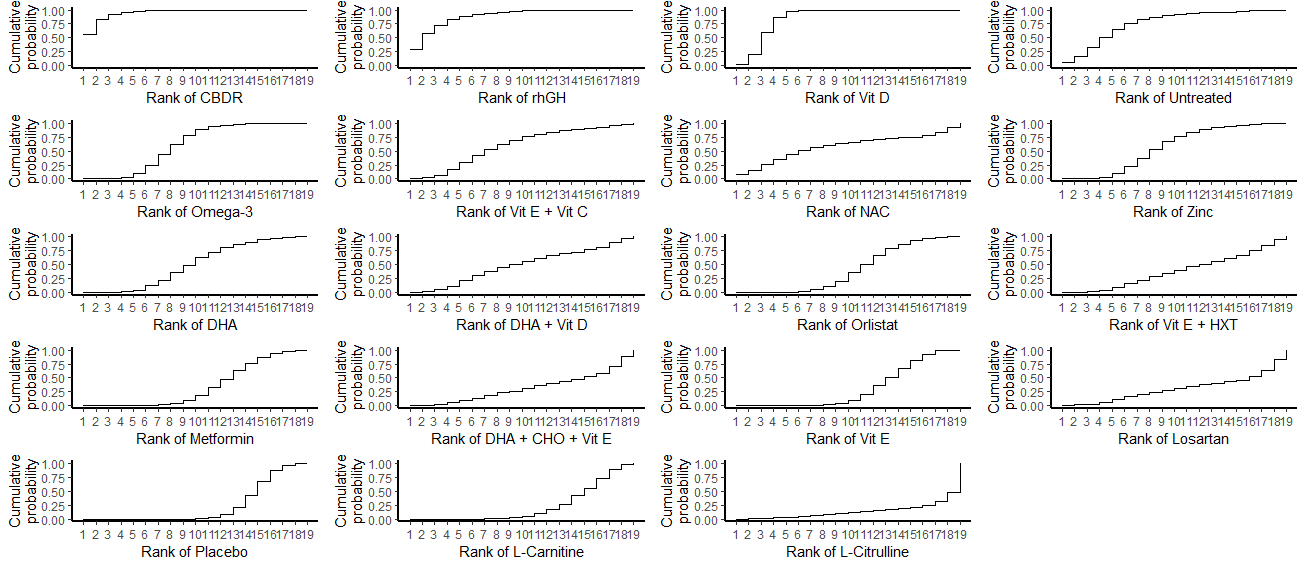


Figure S41, SUCRA plots showing the cumulative probability of each intervention being among the best for reducing ALT before sensitivity analysis. Higher curves indicate better rankings.


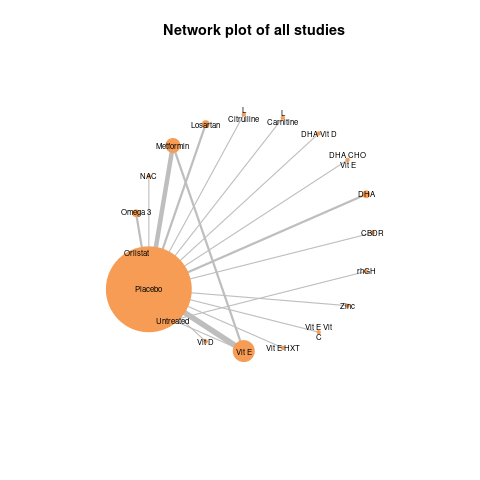


Figure S42, Network geometry of ALT before sensitivity analysis for all included studies, by drug treatments.


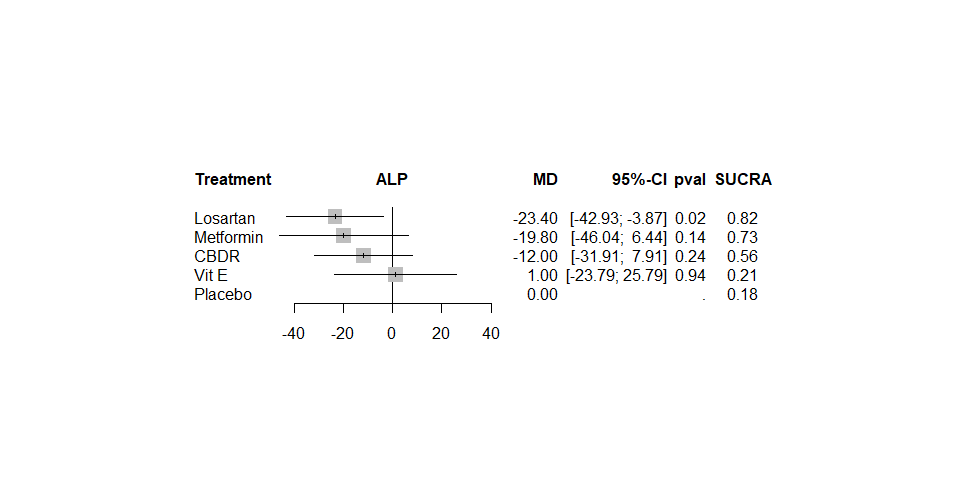


Figure S43, Forest plots for network meta-analysis of ALP.


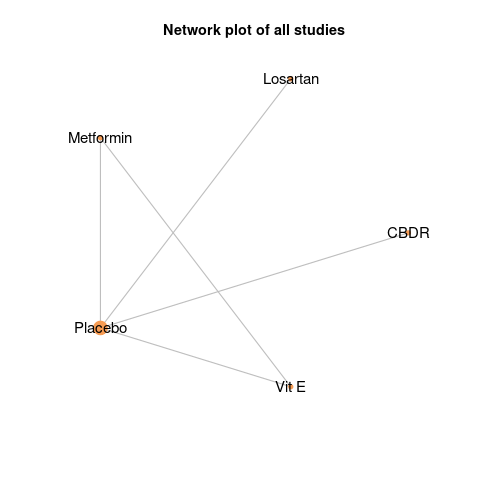


Figure S44, Network geometry of ALP for all included studies, by drug treatments.


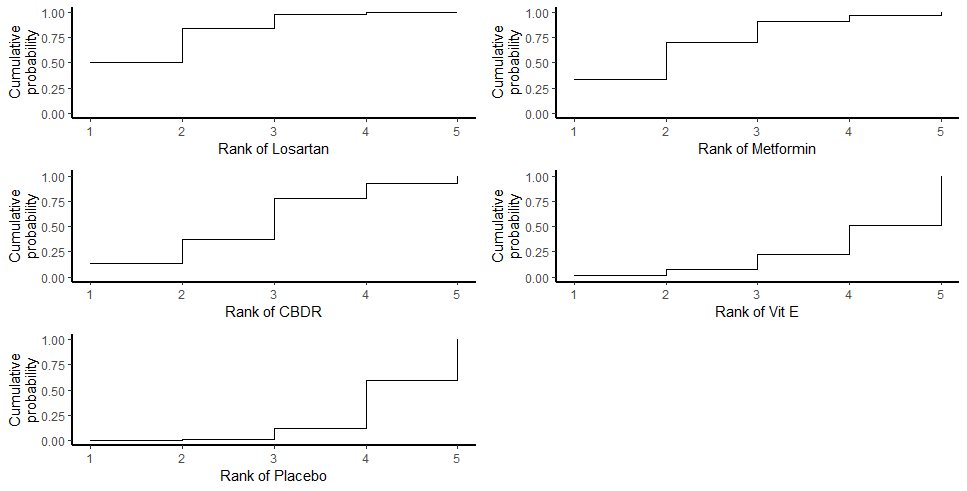


Figure S45, SUCRA plots showing the cumulative probability of each intervention being among the best for reducing ALP. Higher curves indicate better rankings.


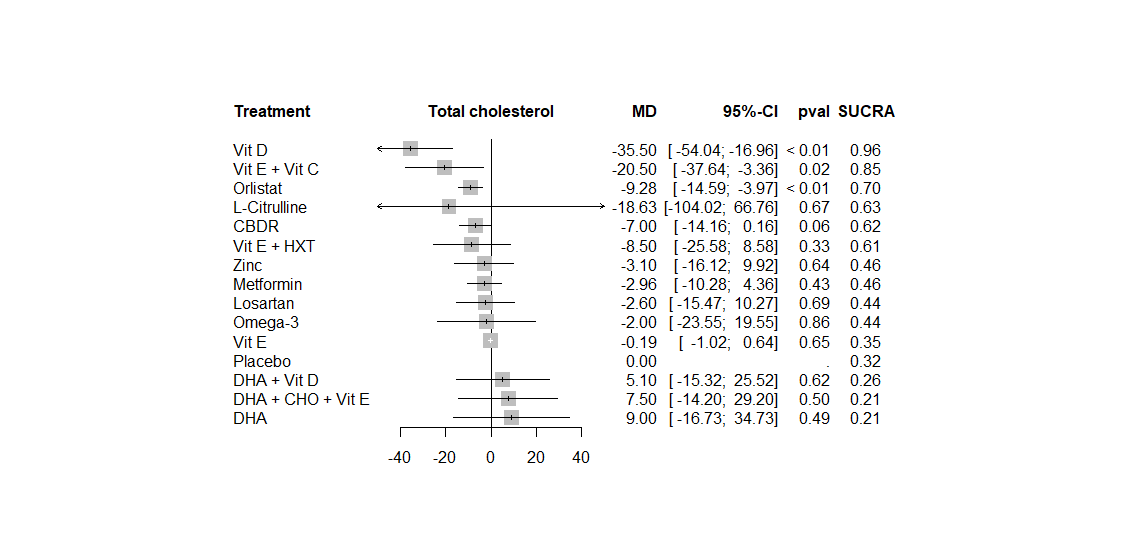


Figure S46, Forest plots for network meta-analysis Total cholesterol after sensitivity analysis.


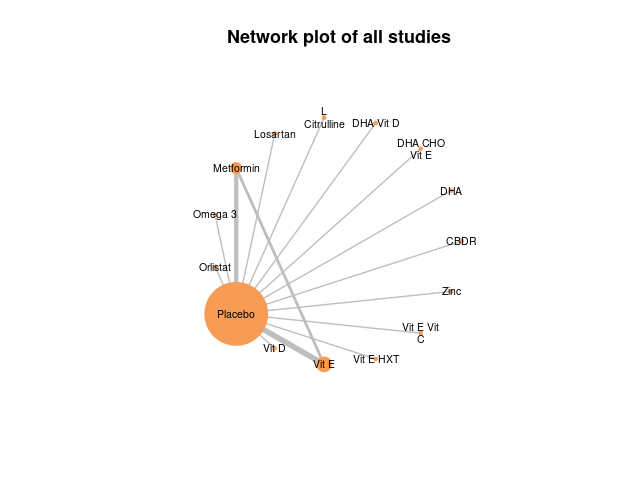


Figure S47, Network geometry of Total cholesterol after sensitivity analysis for all included studies, by drug treatments.


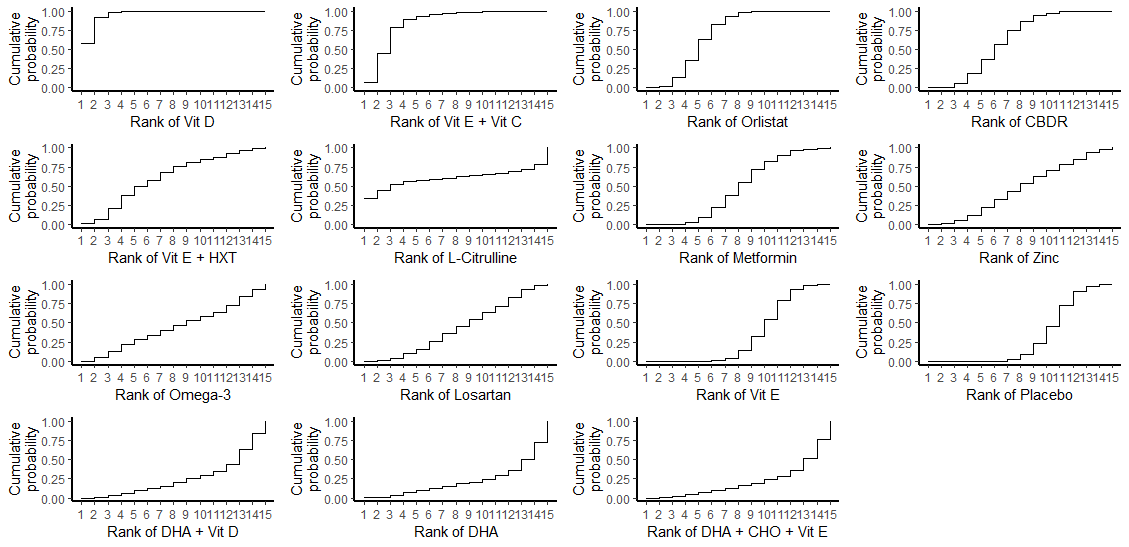


Figure S48, SUCRA plots showing the cumulative probability of each intervention being among the best for reducing Total cholesterol after sensitivity analysis. Higher curves indicate better rankings.


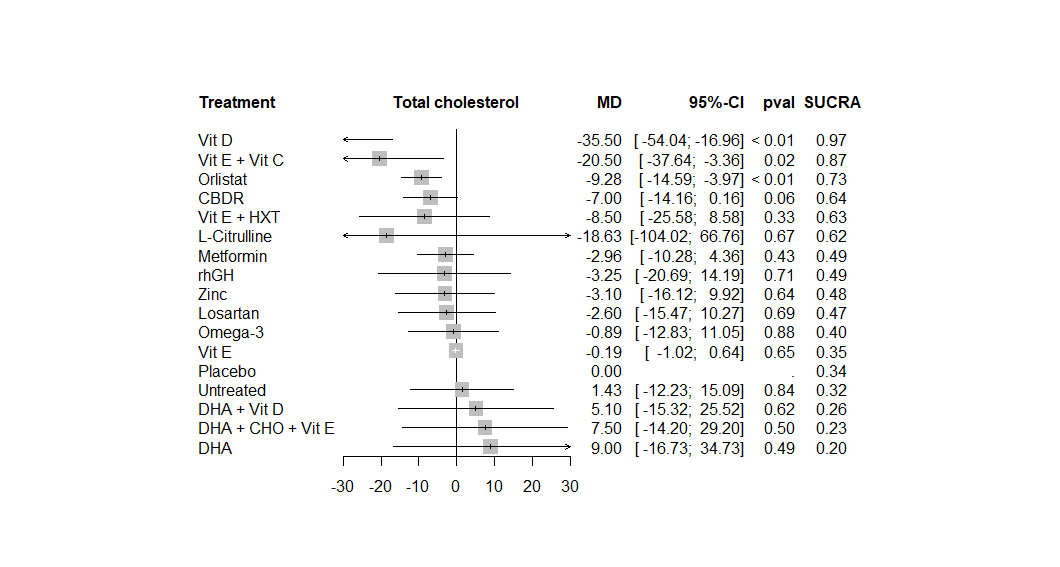


Figure S49, Forest plots for network meta-analysis of Total cholesterol before sensitivity analysis.


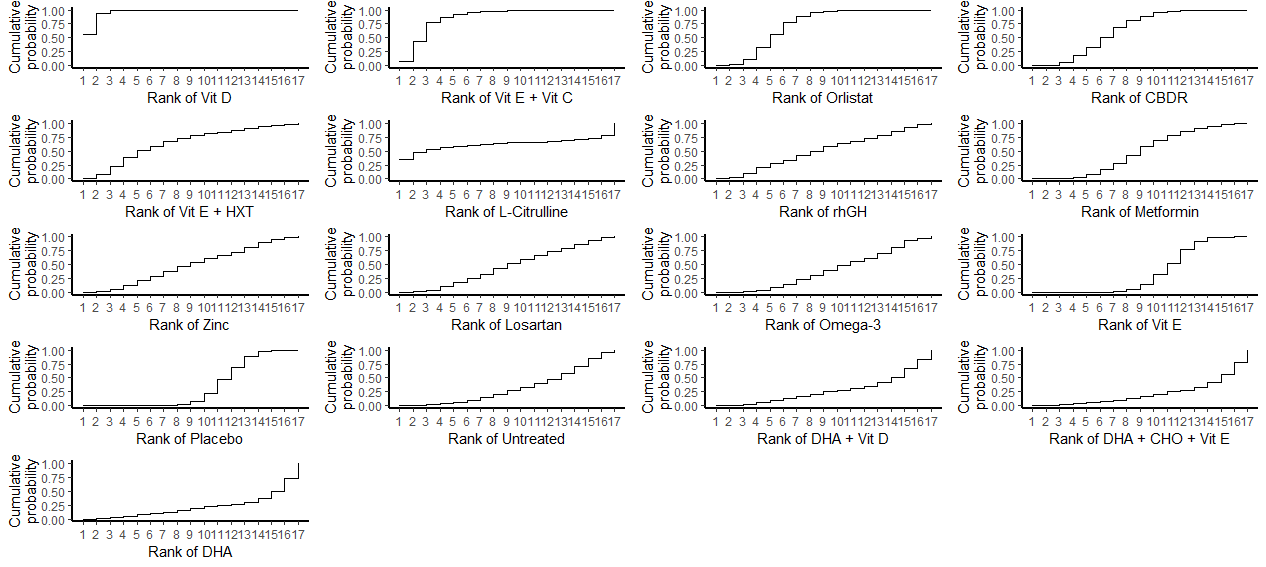


Figure S50 , SUCRA plots showing the cumulative probability of each intervention being among the best for reducing Total cholesterol before sensitivity analysis. Higher curves indicate better rankings.


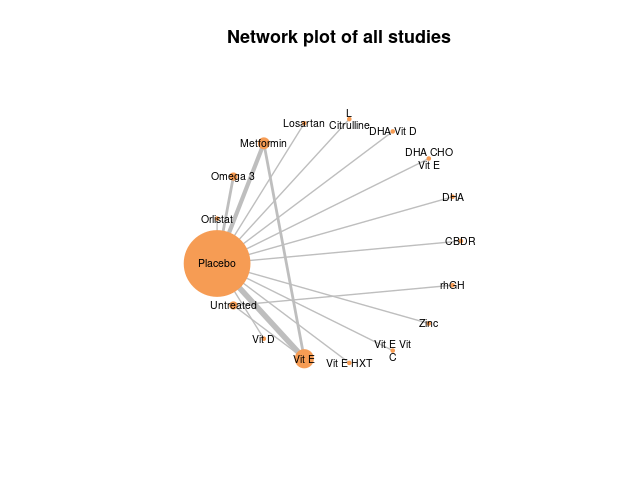


Figure S51 , Network geometry of Total cholesterol before sensitivity analysis for all included studies, by drug treatments.


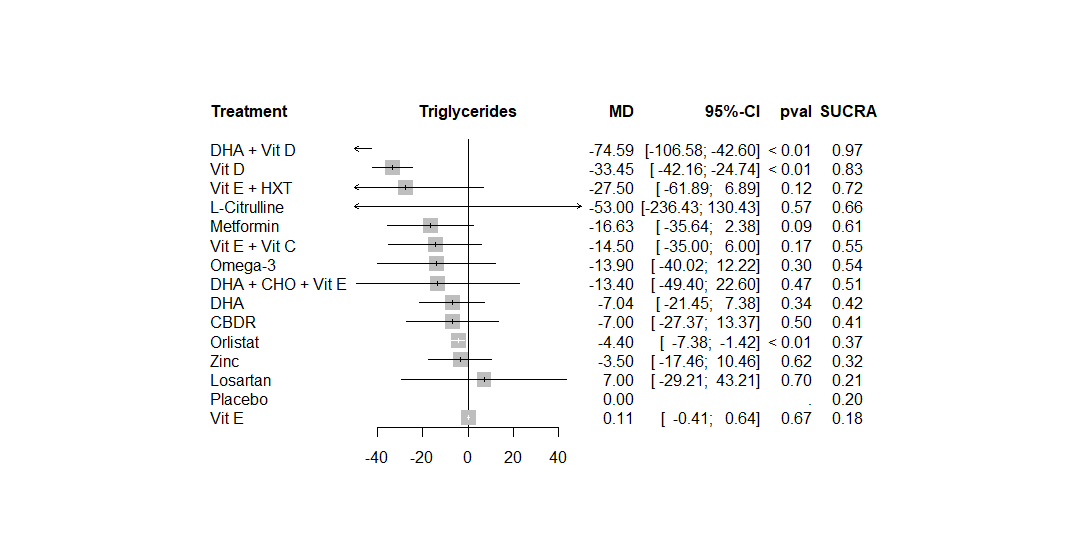


Figure S52, Forest plots for network meta-analysis of Triglycerides after sensitivity analysis.


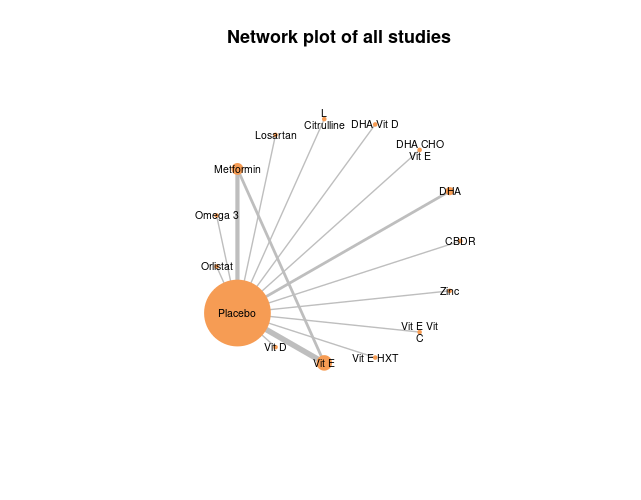


Figure S53, Network geometry of Triglycerides after sensitivity analysis for all included studies, by drug treatments.


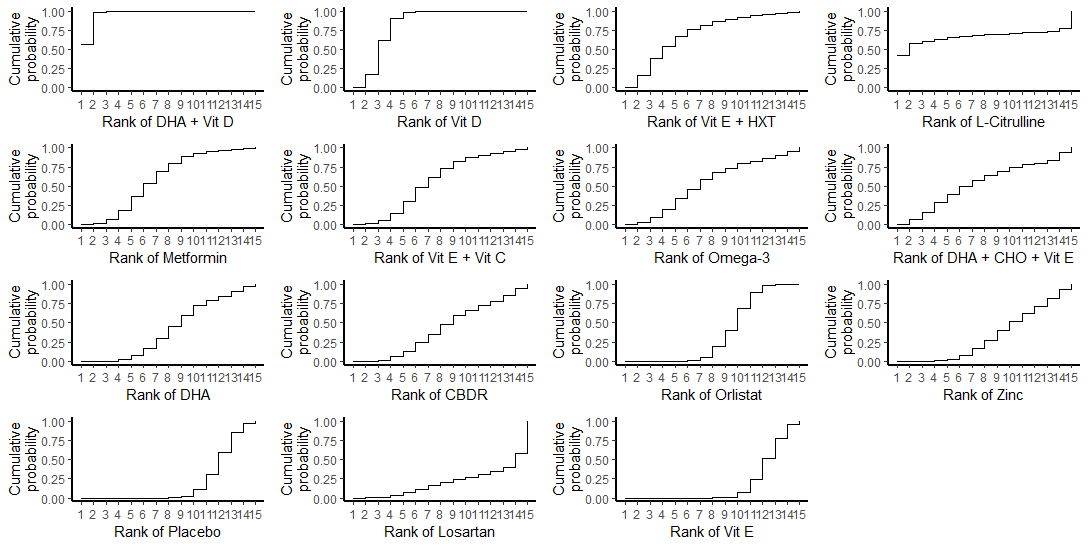


Figure S54, SUCRA plots showing the cumulative probability of each intervention being among the best for reducing triglycerides after sensitivity analysis. Higher curves indicate better rankings.


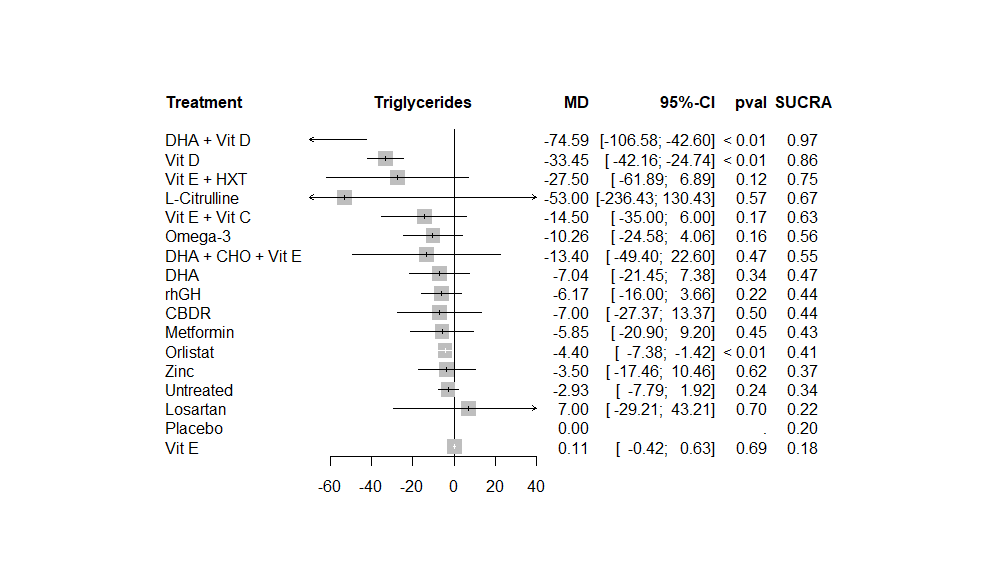


Figure S55 , Forest plots for network meta-analysis of Triglycerides before sensitivity analysis.


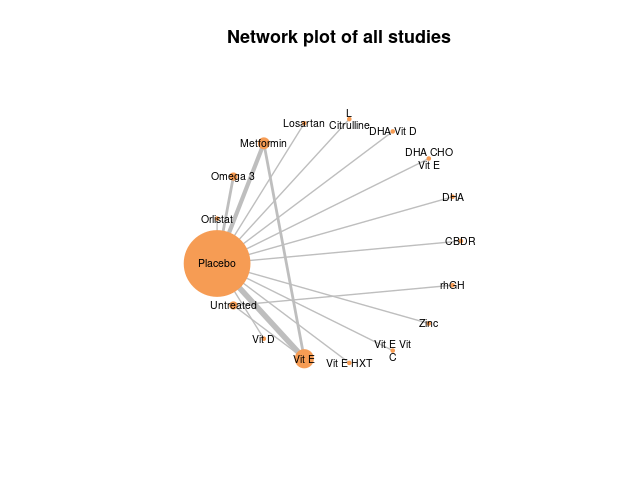


Figure S56 , Network geometry of Triglycerides before sensitivity analysis for all included studies, by drug treatments.


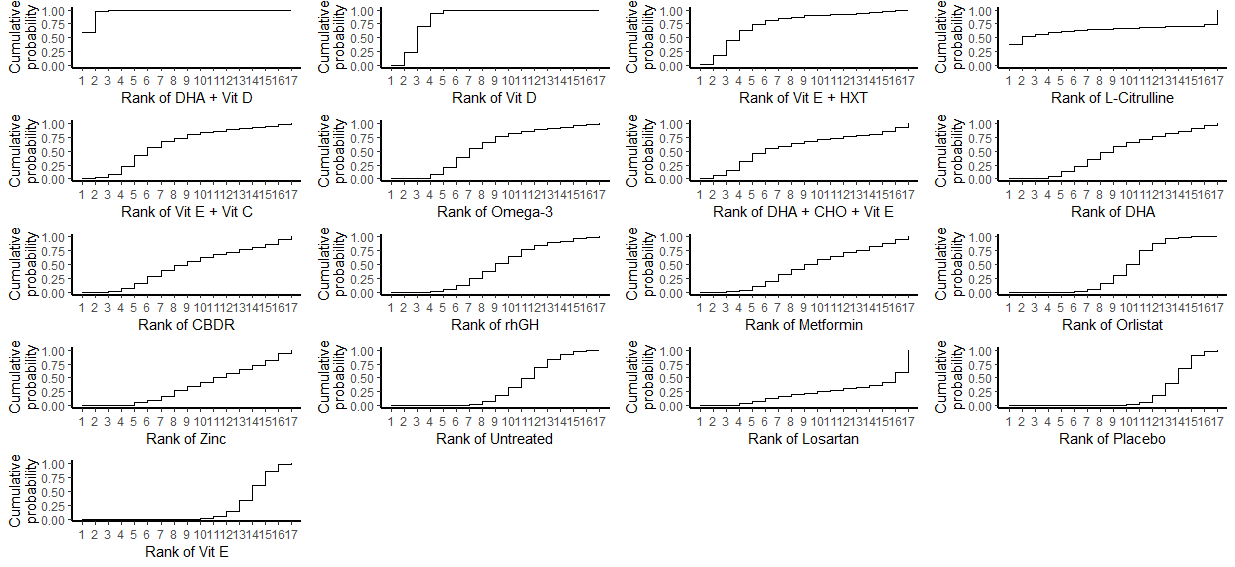


Figure S57, SUCRA plots showing the cumulative probability of each intervention being among the best for reducing Triglycerides before sensitivity analysis. Higher curves indicate better rankings.


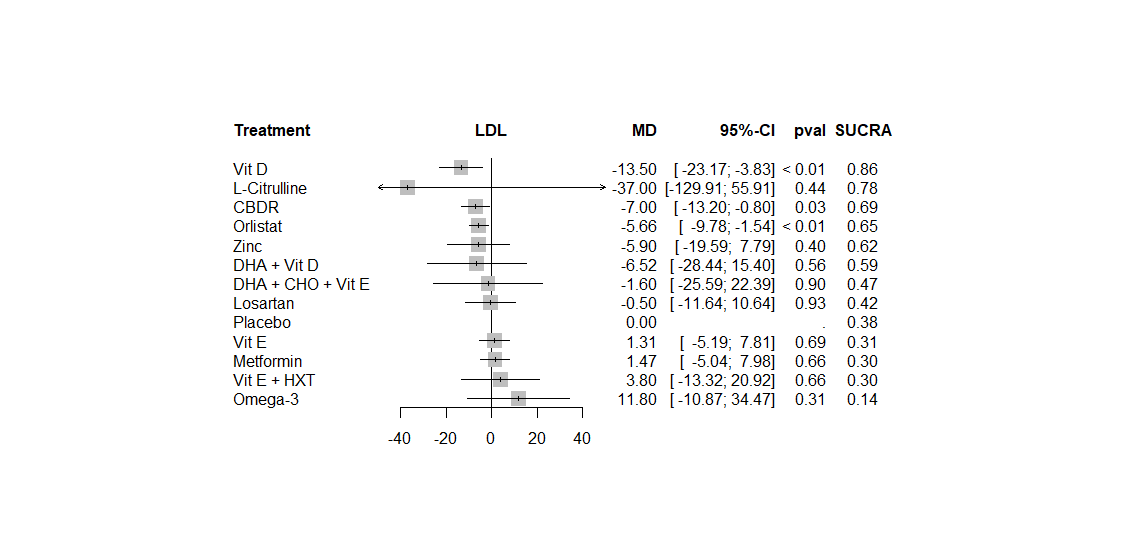


Figure S58, Forest plots for network meta-analysis of LDL after sensitivity analysis.


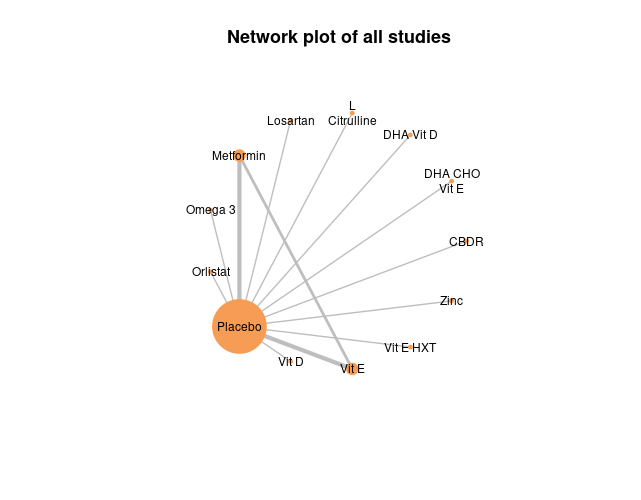


Figure S59, Network geometry of LDL after sensitivity analysis for all included studies, by drug treatments.


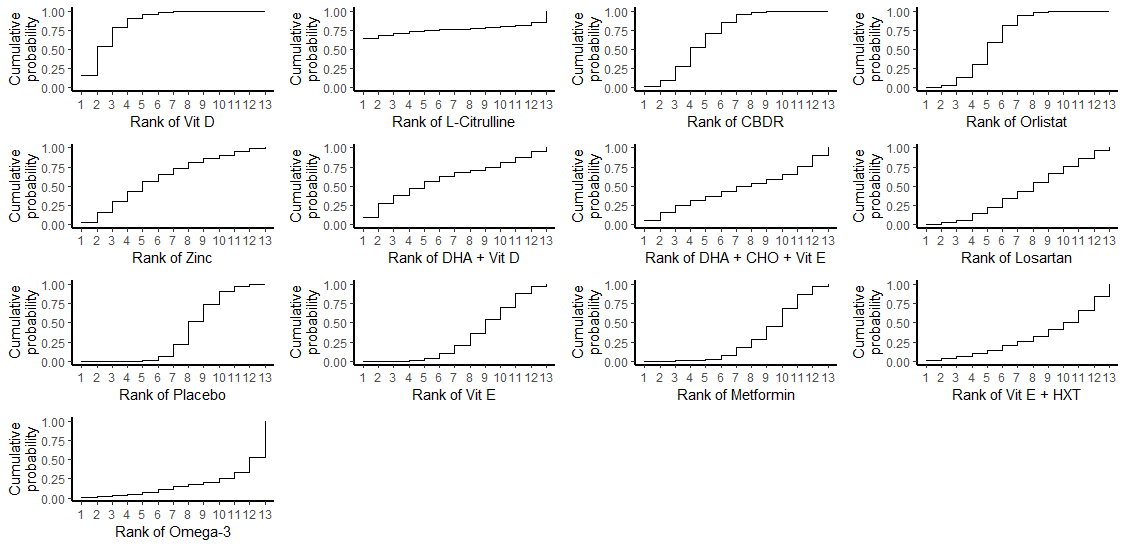


Figure S60, SUCRA plots showing the cumulative probability of each intervention being among the best for reducing LDL after sensitivity analysis. Higher curves indicate better rankings.


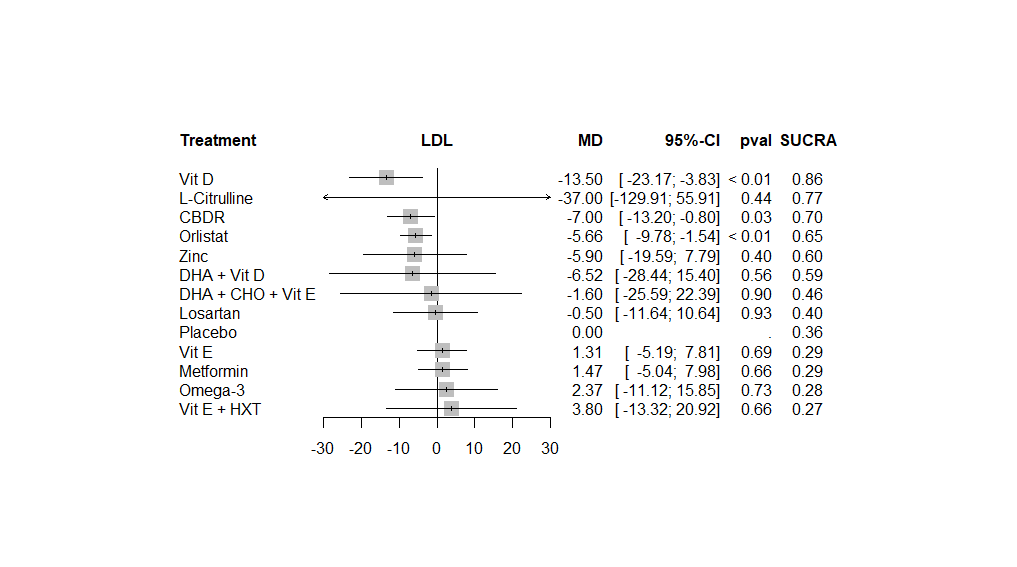


Figure S61 , Forest plots for network meta-analysis of LDL before sensitivity analysis.


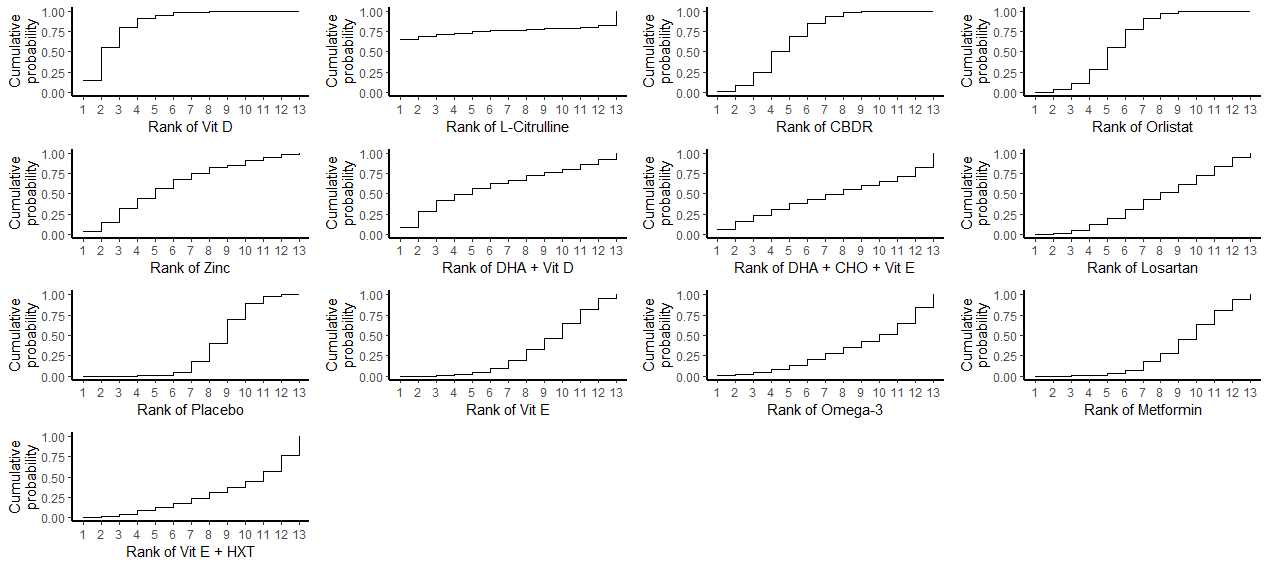


Figure S62 , SUCRA plots showing the cumulative probability of each intervention being among the best for reducing LDL before sensitivity analysis. Higher curves indicate better rankings.


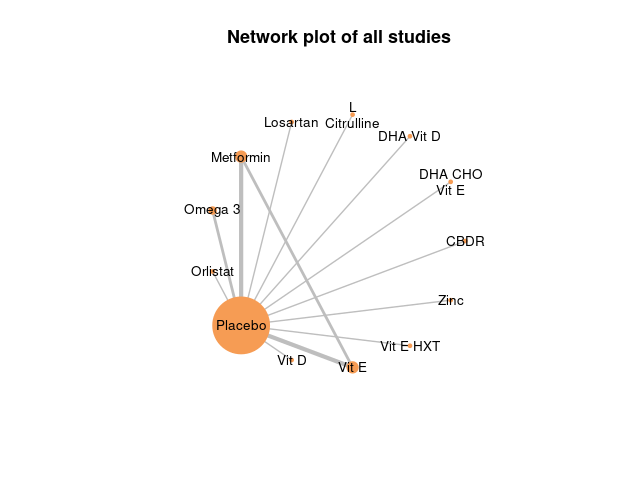


Figure S63 , Network geometry of LDL before sensitivity analysis for all included studies, by drug treatments.


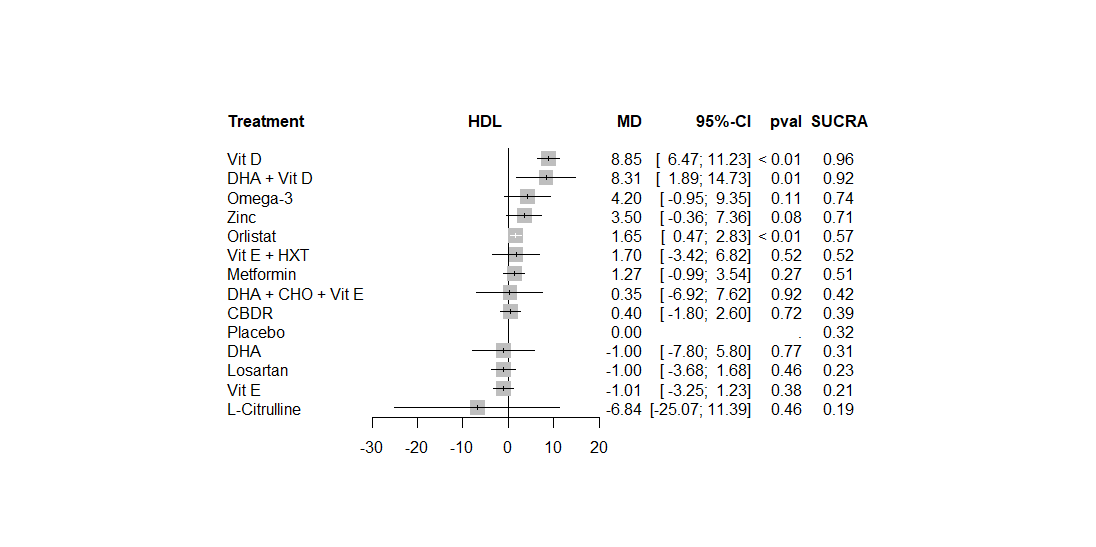


Figure S64, Forest plots for network meta-analysis of HDL after sensitivity analysis.


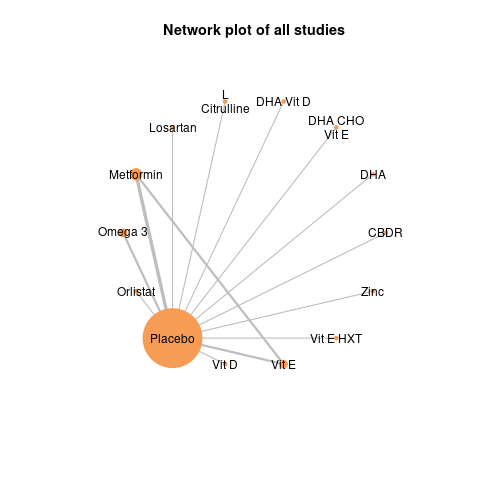


Figure S65, Network geometry of HDL after sensitivity analysis for all included studies, by drug treatments.


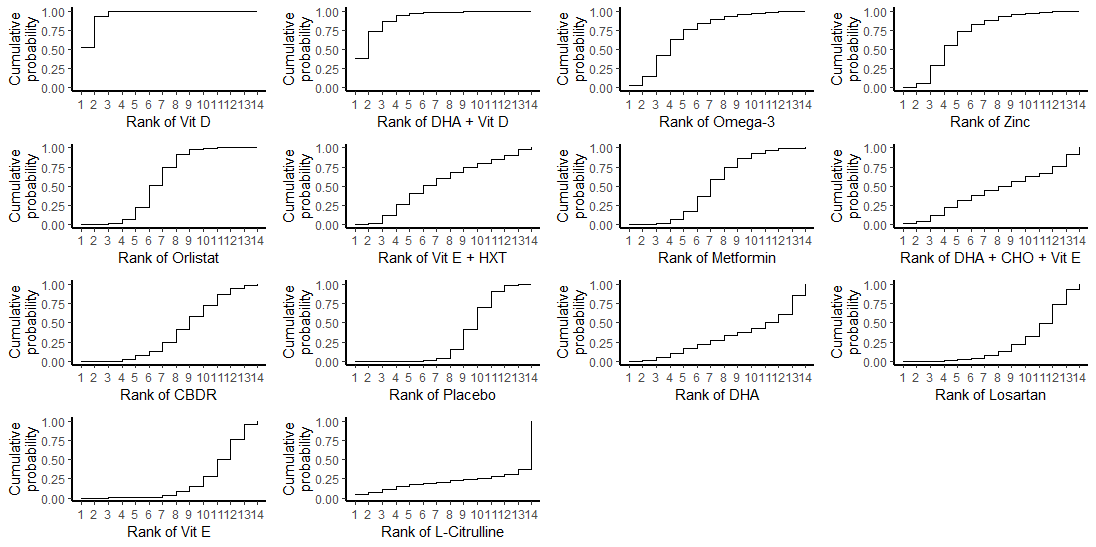


Figure S66, SUCRA plots showing the cumulative probability of each intervention being among the best for reducing HDL after sensitivity analysis. Higher curves indicate better rankings.


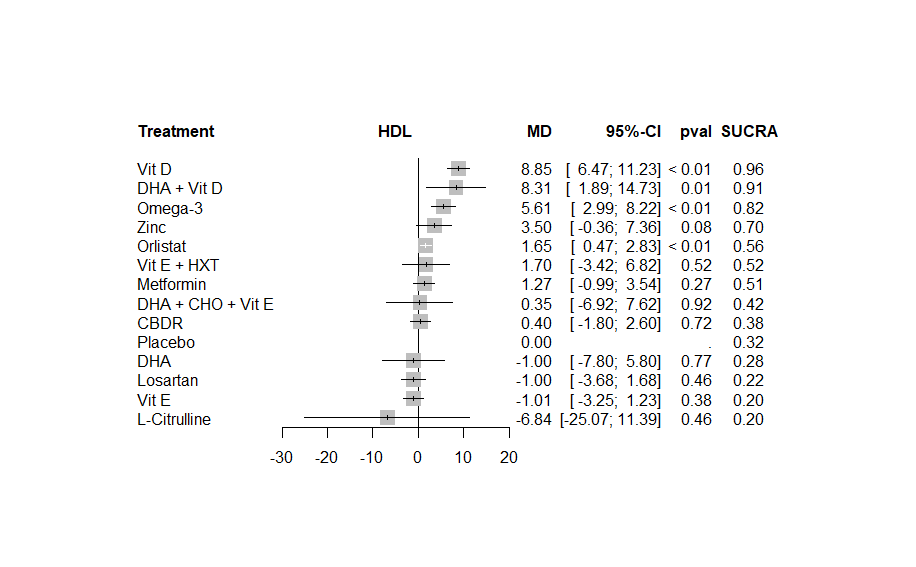


Figure S67 , Forest plots for network meta-analysis of HDL before sensitivity analysis.


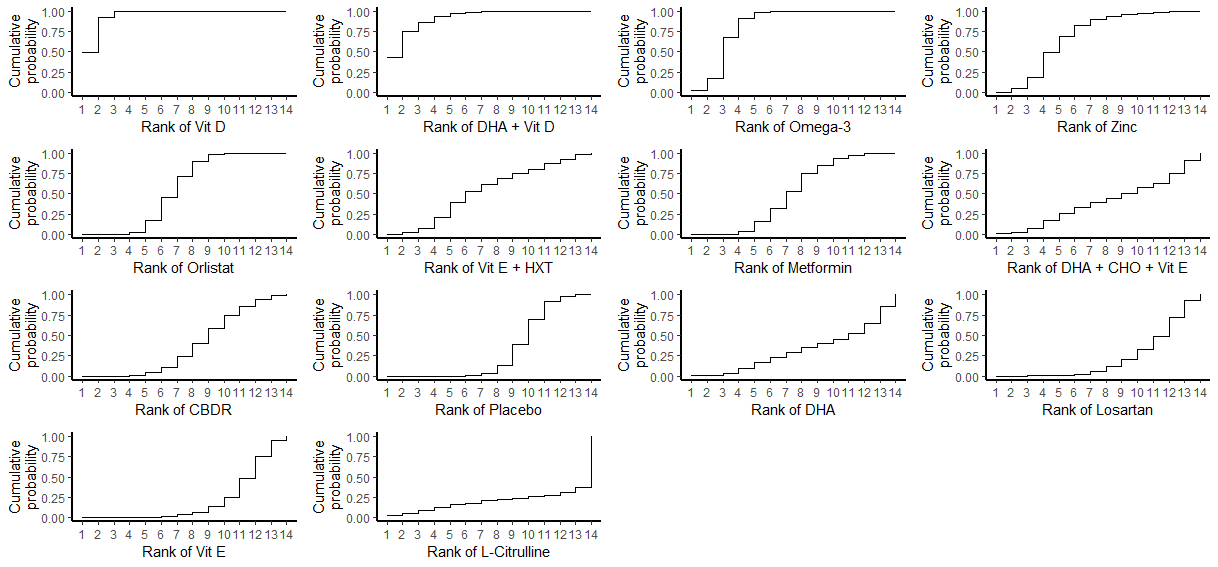


Figure S68 , SUCRA plots showing the cumulative probability of each intervention being among the best for reducing HDL before sensitivity analysis. Higher curves indicate better rankings.


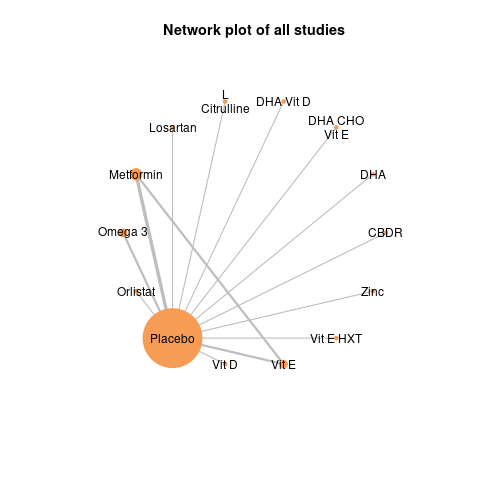


Figure S69 , Network geometry of HDL before sensitivity analysis for all included studies, by drug treatments.


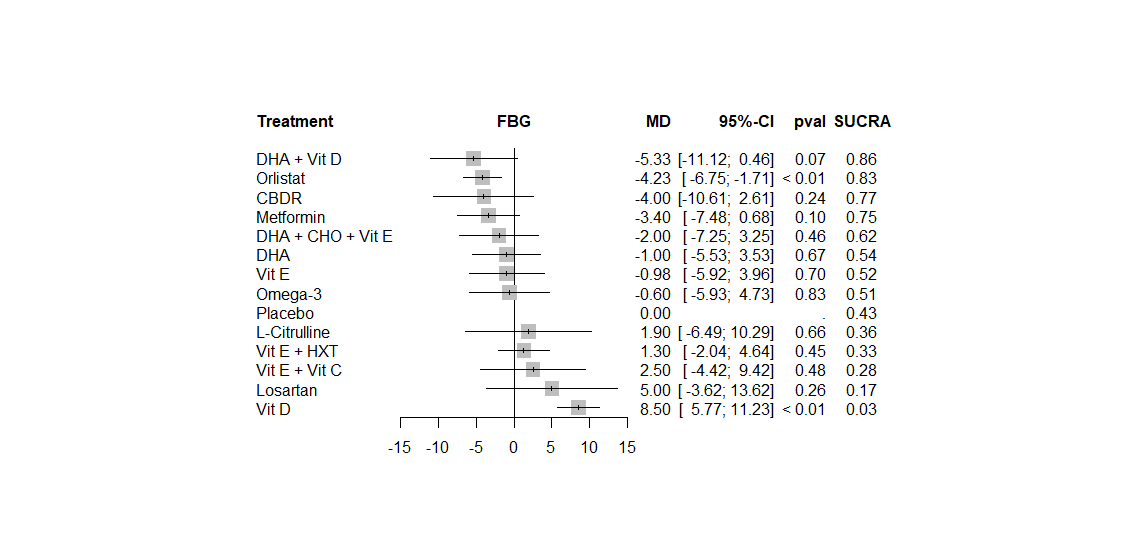


Figure S70, Forest plots for network meta-analysis of Fasting Blood Glucose after sensitivity analysis.


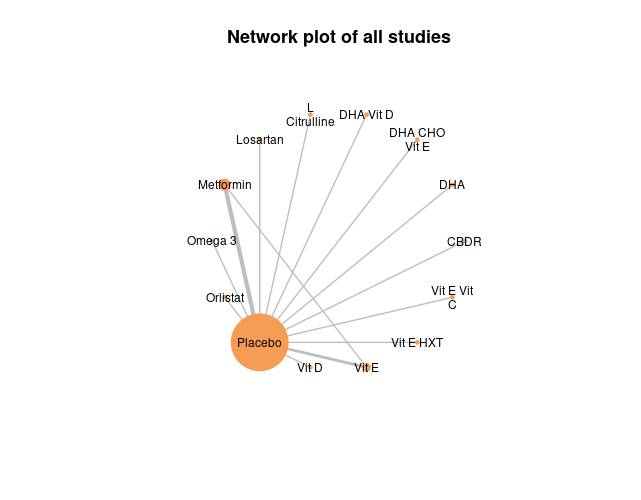


Figure S71, Network geometry of Fasting Blood Glucose after sensitivity analysis for all included studies, by drug treatments.


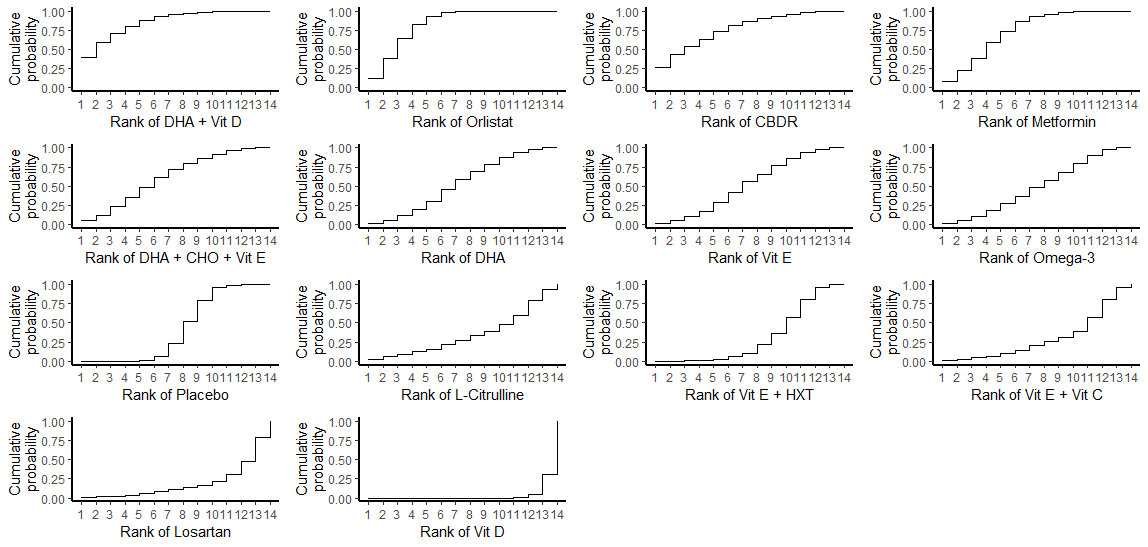


Figure S72, SUCRA plots showing the cumulative probability of each intervention being among the best for reducing Fasting Blood Glucose after sensitivity analysis. Higher curves indicate better rankings.


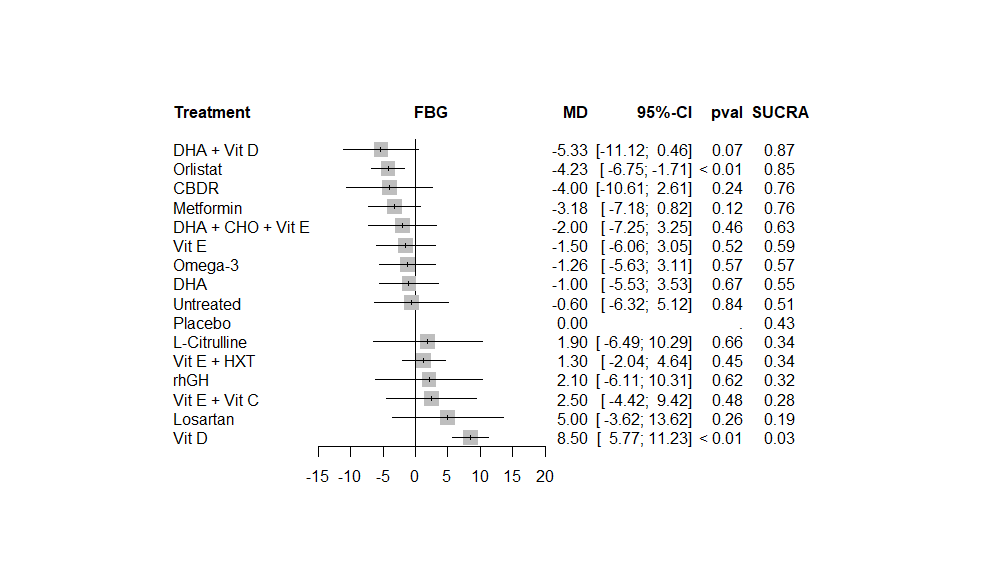


Figure S73 , Forest plots for network meta-analysis of Fasting Blood Glucose before sensitivity analysis.


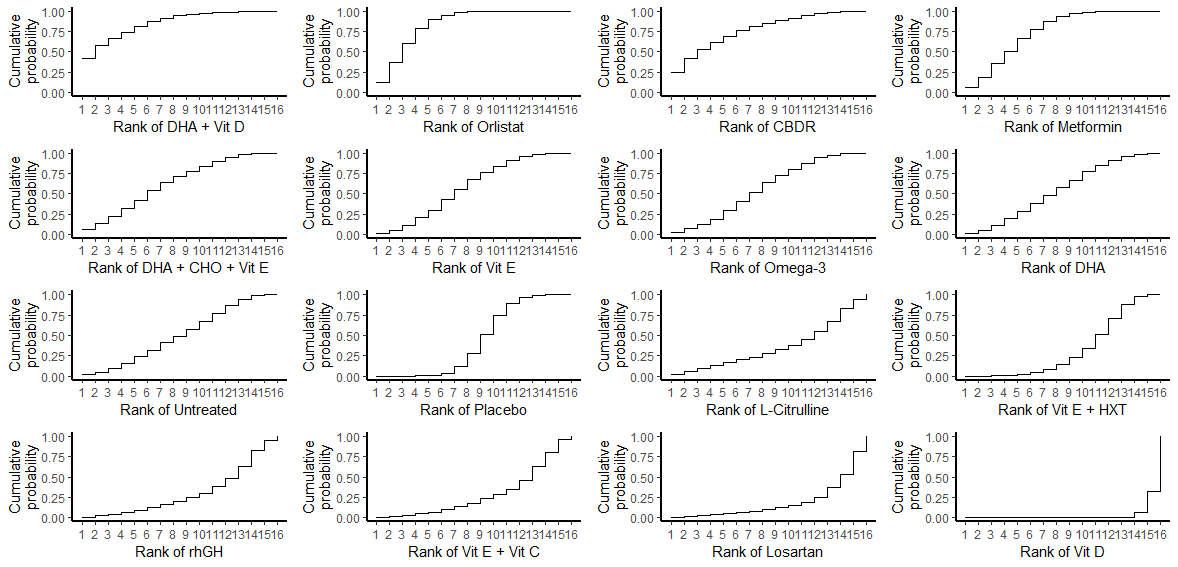


Figure S74 , SUCRA plots showing the cumulative probability of each intervention being among the best for reducing Fasting Blood Glucose before sensitivity analysis. Higher curves indicate better rankings.


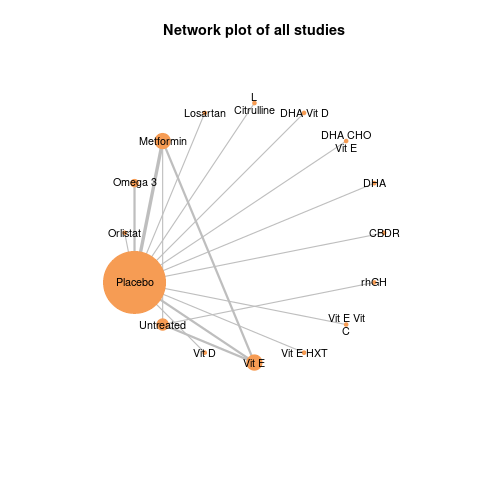


Figure S75 , Network geometry of Fasting Blood Glucose before sensitivity analysis for all included studies, by drug treatments.


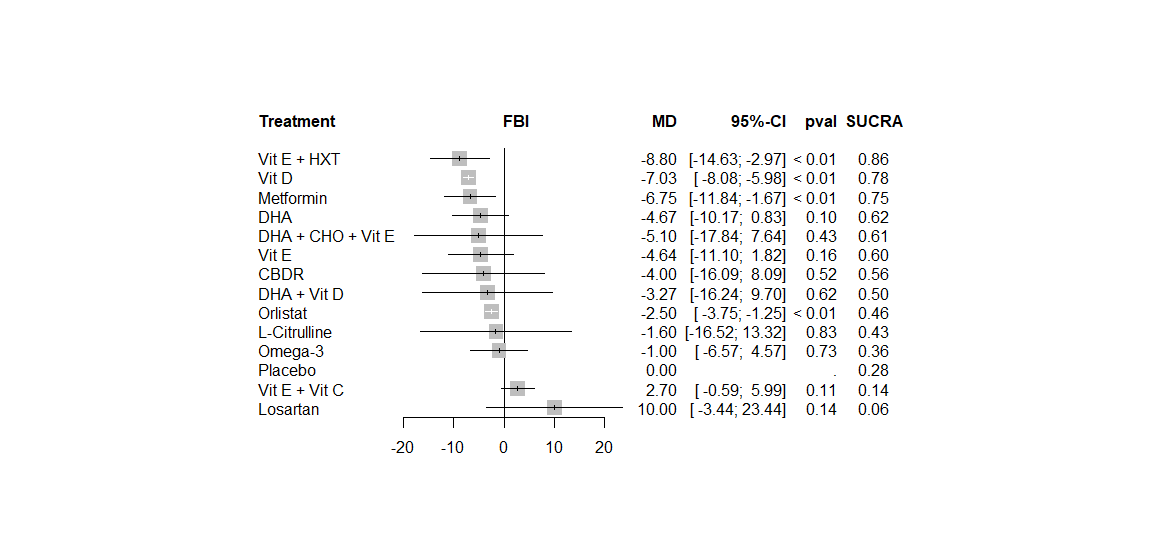


Figure S76 Forest plots for network meta-analysis of FBI after sensitivity analysis.


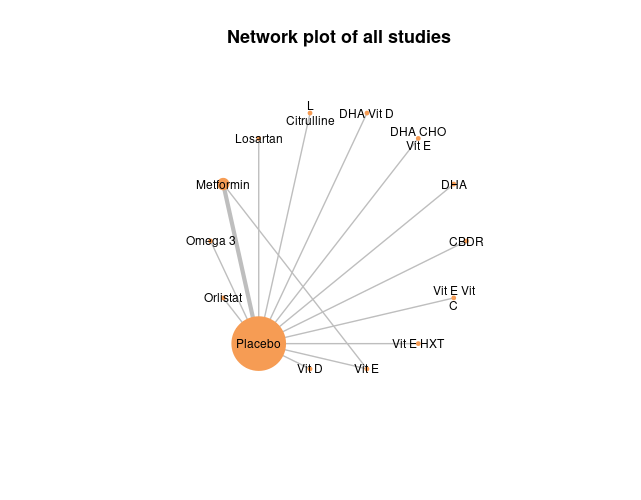


Figure S77 Network geometry of FBI after sensitivity analysis for all included studies, by drug treatments.


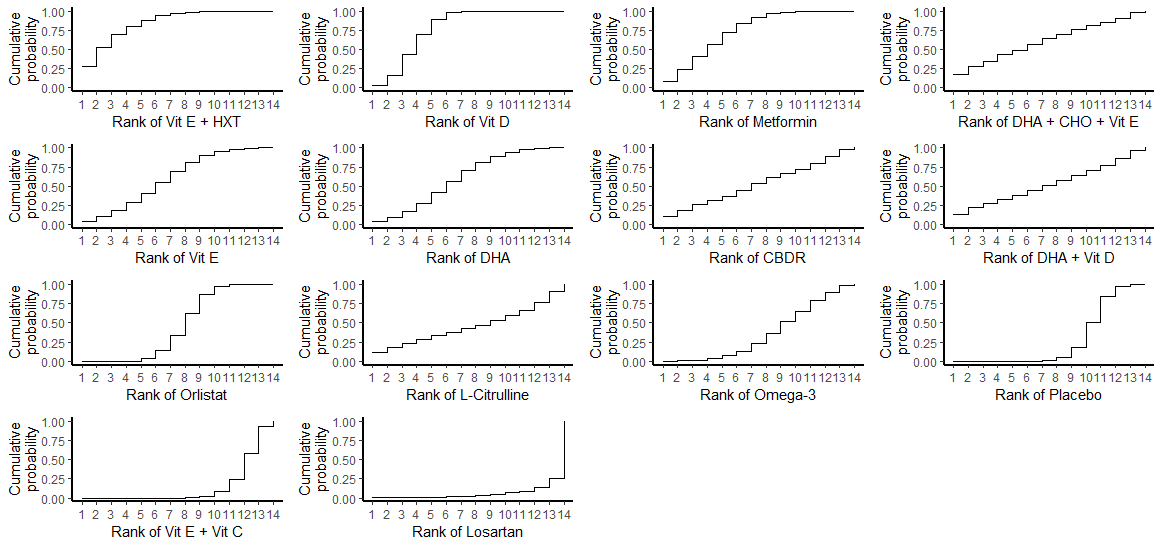


Figure S78, SUCRA plots showing the cumulative probability of each intervention being among the best for reducing FBI after sensitivity analysis. Higher curves indicate better rankings.


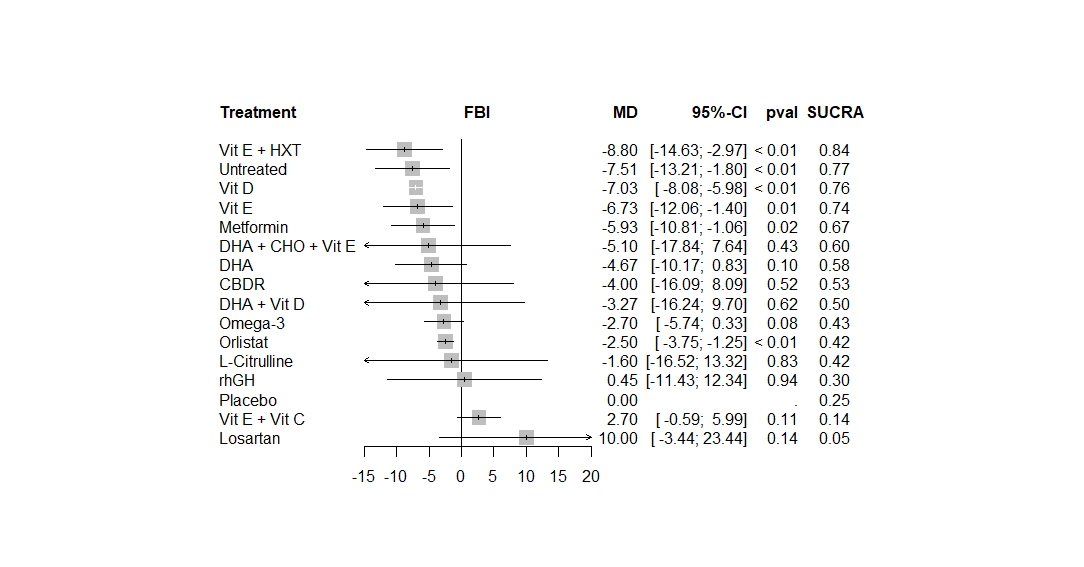


Figure S79 , Forest plots for network meta-analysis of FBI before sensitivity analysis.


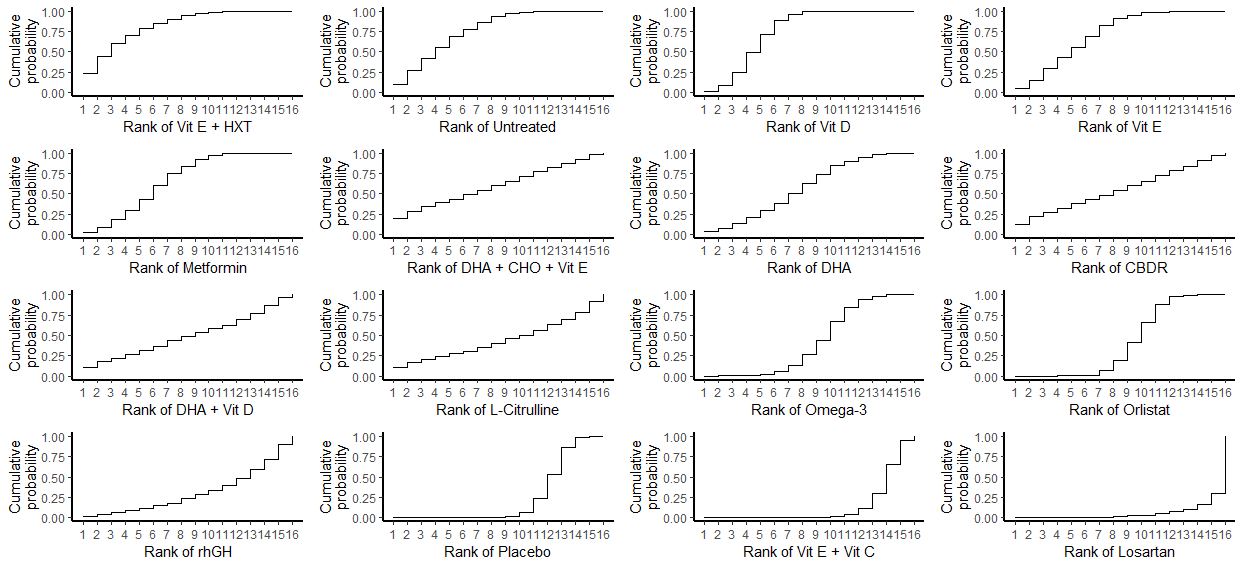


Figure S80 , SUCRA plots showing the cumulative probability of each intervention being among the best for reducing FBI before sensitivity analysis. Higher curves indicate better rankings.


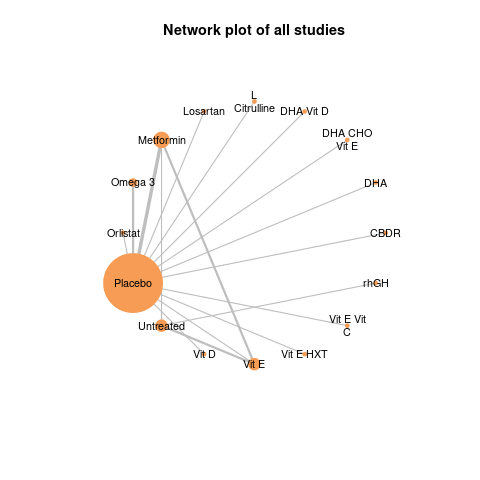


Figure S81 , Network geometry of FBI before sensitivity analysis for all included studies, by drug treatments.


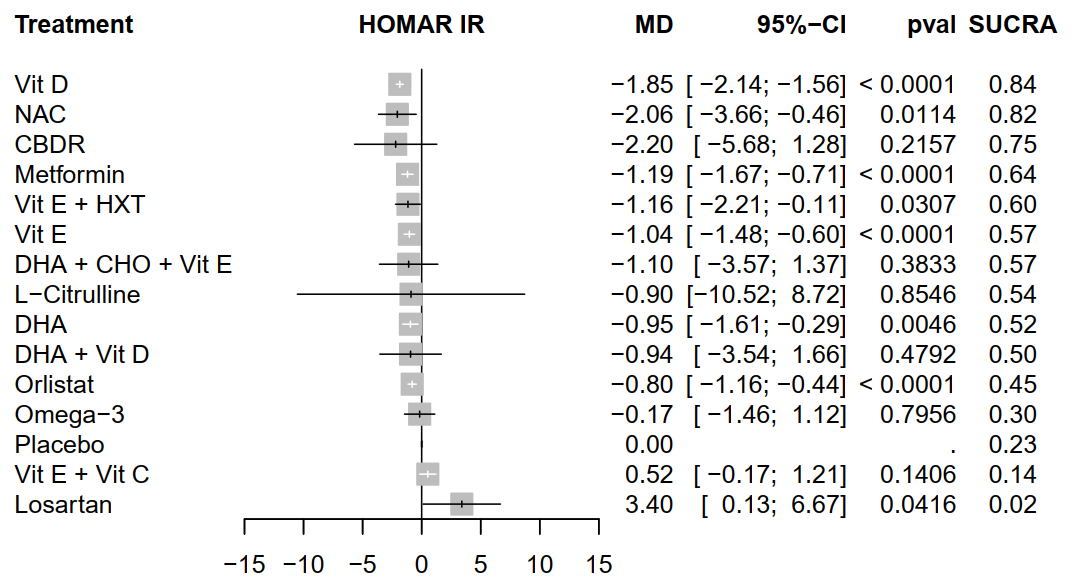


Figure S82, Forest plots for network meta-analysis of HOMA-IR after sensitivity analysis.


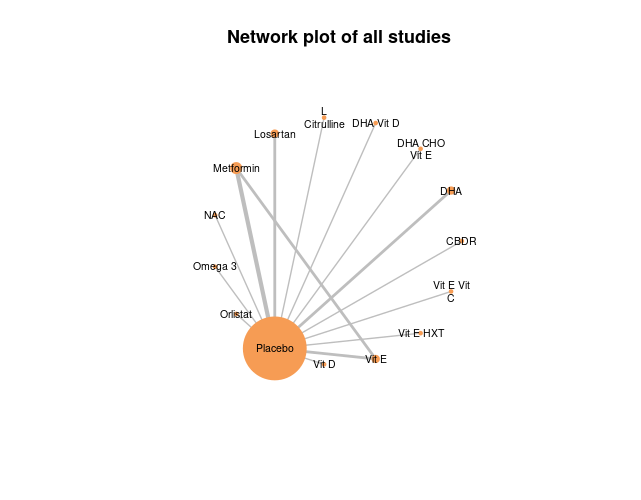


Figure S83, Network geometry of HOMA-IR after sensitivity analysis for all included studies, by drug treatments.


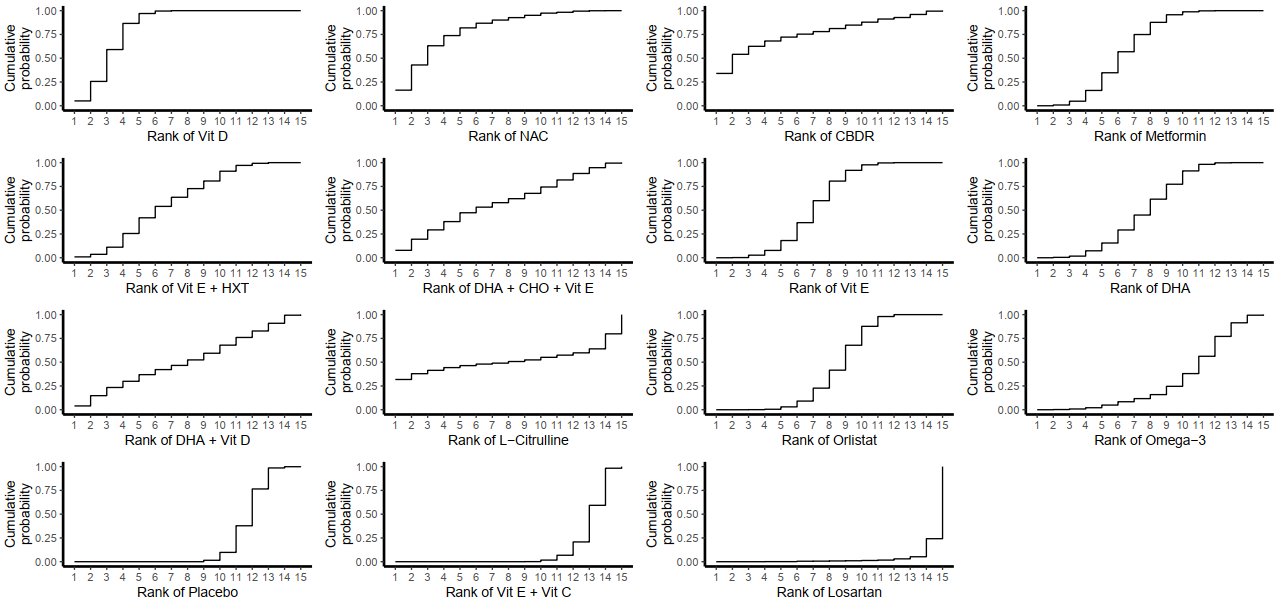


Figure S84, SUCRA plots showing the cumulative probability of each intervention being among the best for reducing HOMA-IR after sensitivity analysis. Higher curves indicate better rankings.


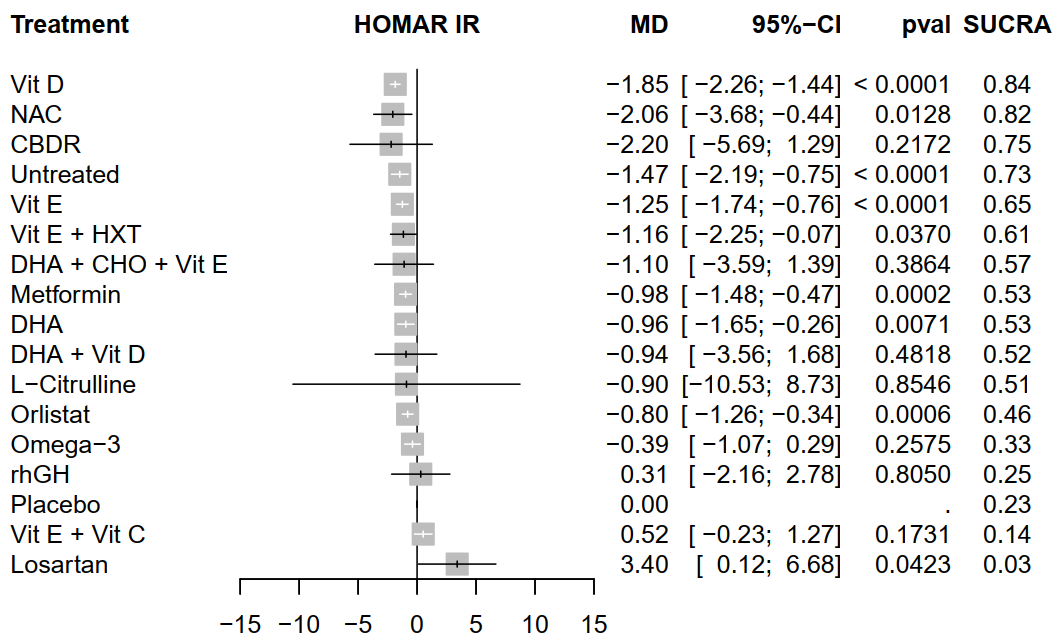


Figure S85 , Forest plots for network meta-analysis of HOMA-IR before sensitivity analysis.


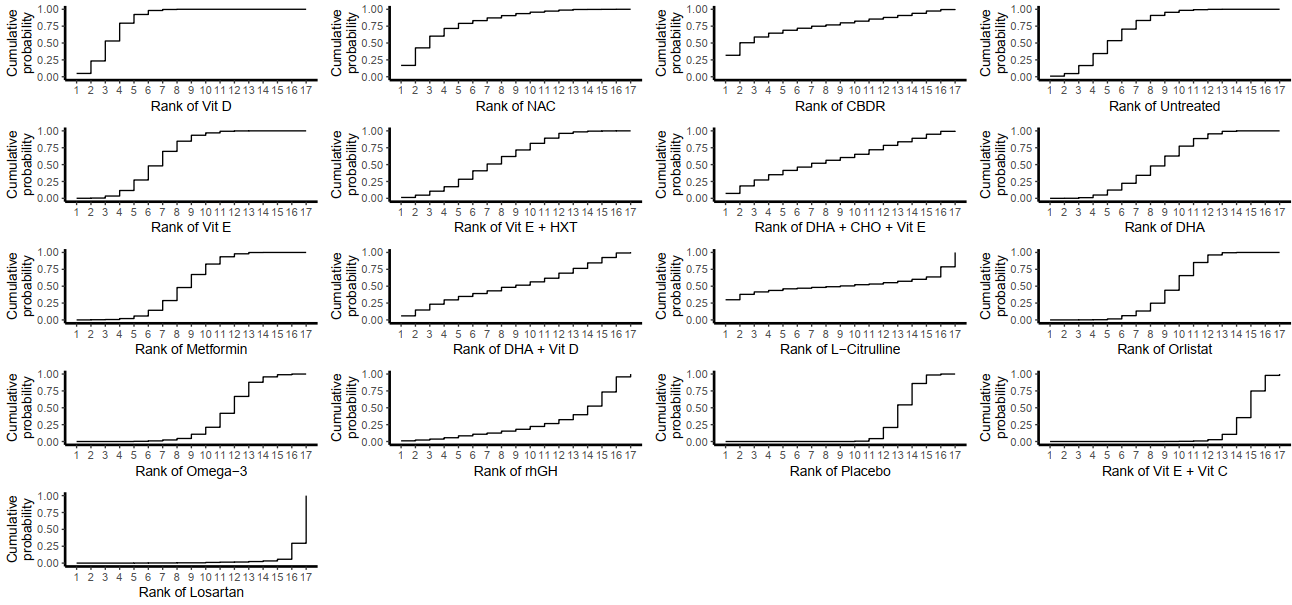


Figure S86 , SUCRA plots showing the cumulative probability of each intervention being among the best for reducing HOMA-IR before sensitivity analysis. Higher curves indicate better rankings.


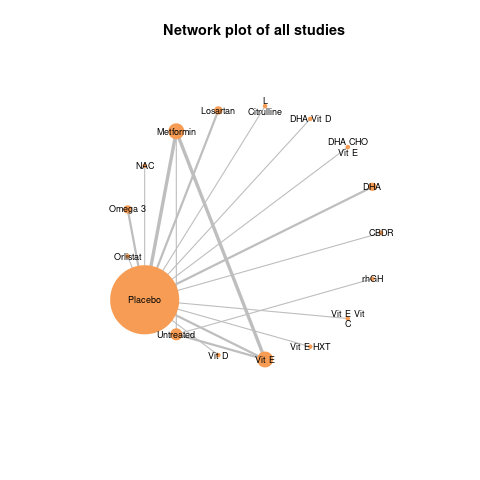


Figure S87 , Network geometry of HOMA-IR before sensitivity analysis for all included studies, by drug treatments.


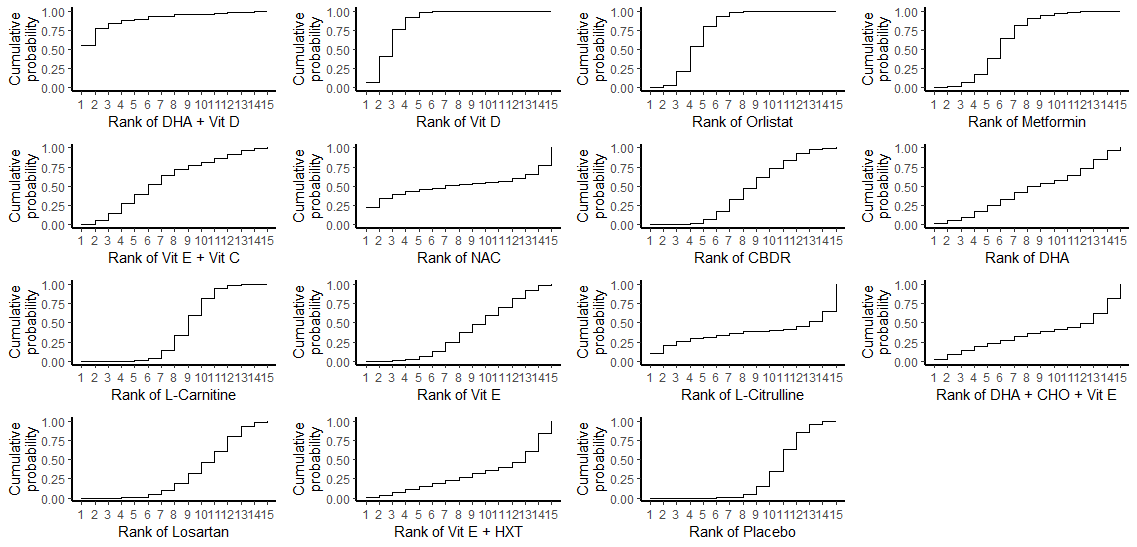


Figure S88, SUCRA plots showing the cumulative probability of each intervention being among the best for reducing BMI after sensitivity analysis. Higher curves indicate better rankings.


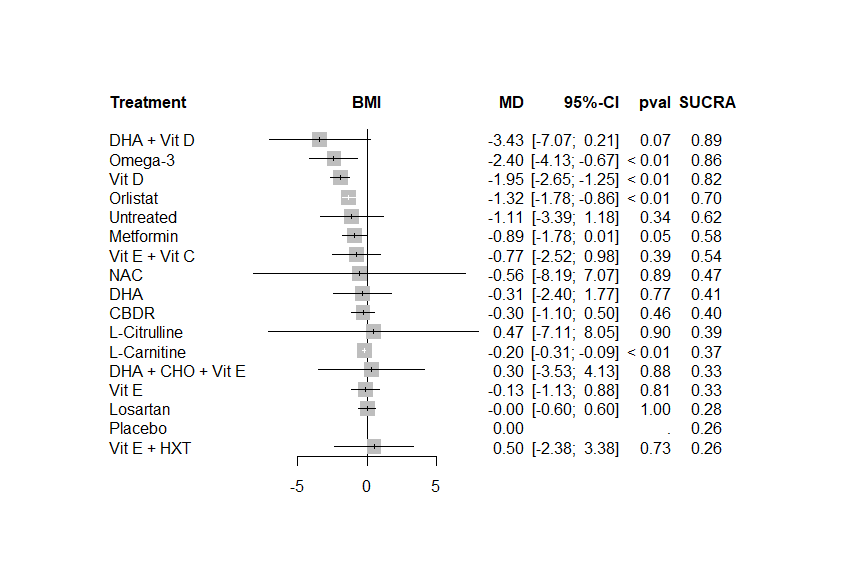


Figure S89 , Forest plots for network meta-analysis of BMI before sensitivity analysis.


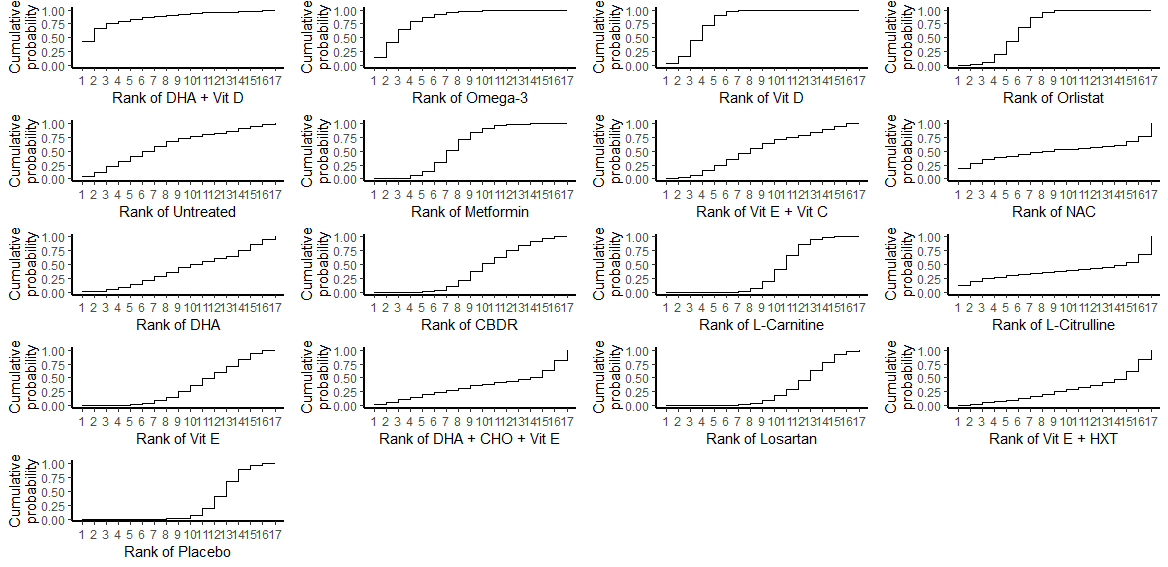


Figure S90 , SUCRA plots showing the cumulative probability of each intervention being among the best for reducing BMI before sensitivity analysis. Higher curves indicate better rankings.


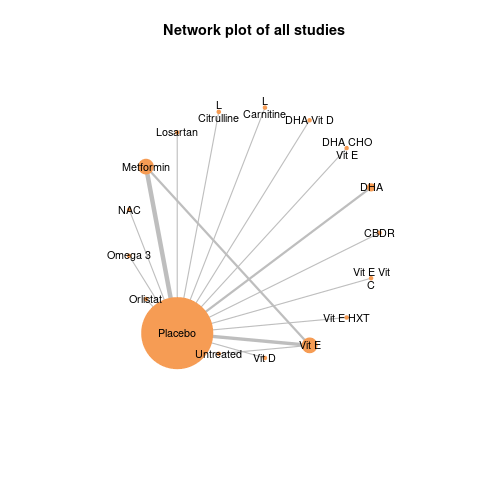


Figure S91 , Network geometry of BMI before sensitivity analysis for all included studies, by drug treatments.


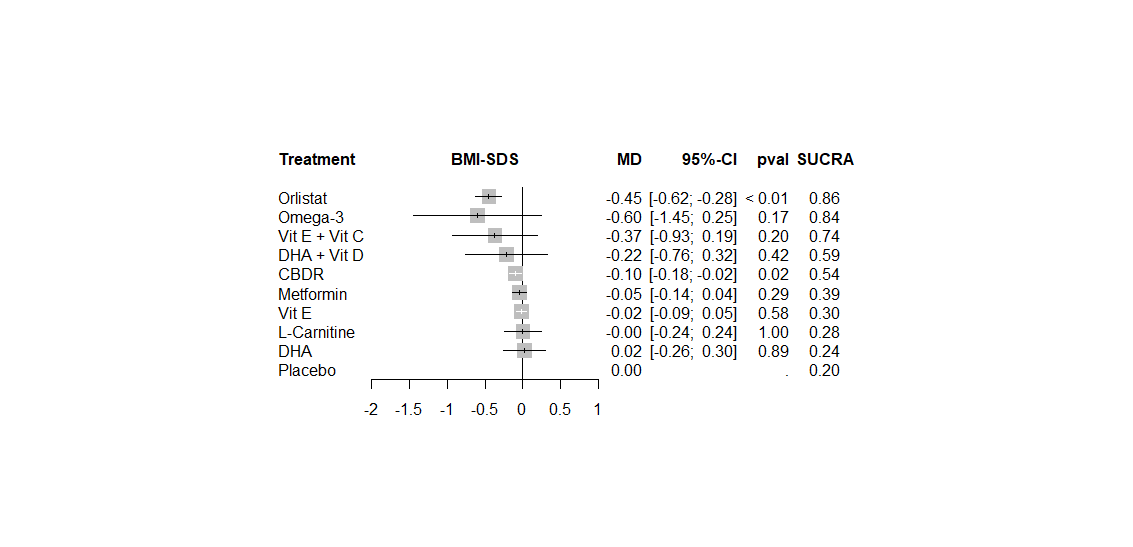


Figure S92, Forest plots for network meta-analysis of BMI-SDS after sensitivity analysis.


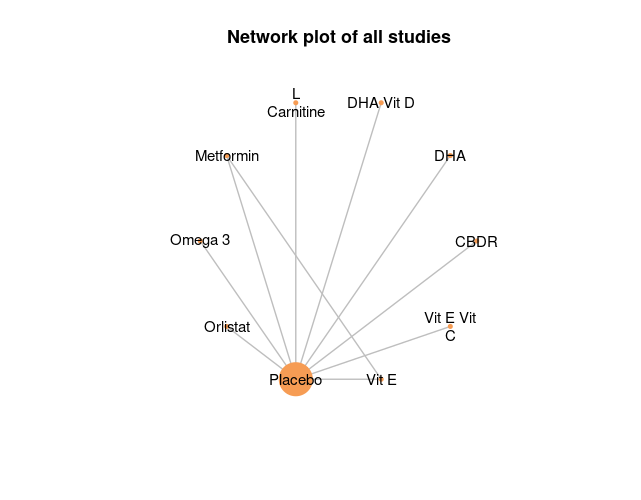


Figure S93, Network geometry of BMI-SDS after sensitivity analysis for all included studies, by drug treatments.


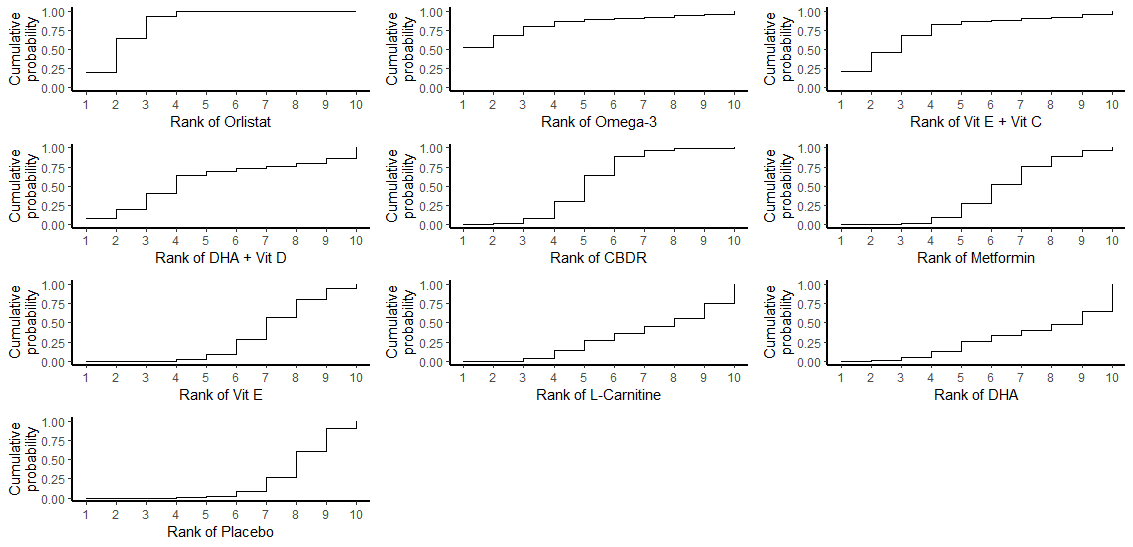


Figure S94, SUCRA plots showing the cumulative probability of each intervention being among the best for reducing BMI-SDS after sensitivity analysis. Higher curves indicate better rankings.


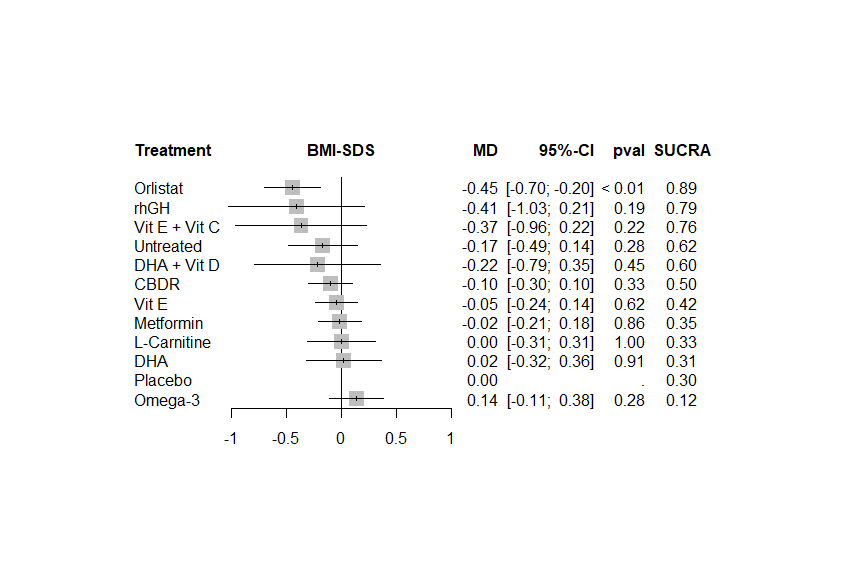


Figure S95 , Forest plots for network meta-analysis of BMI-SDS before sensitivity analysis.


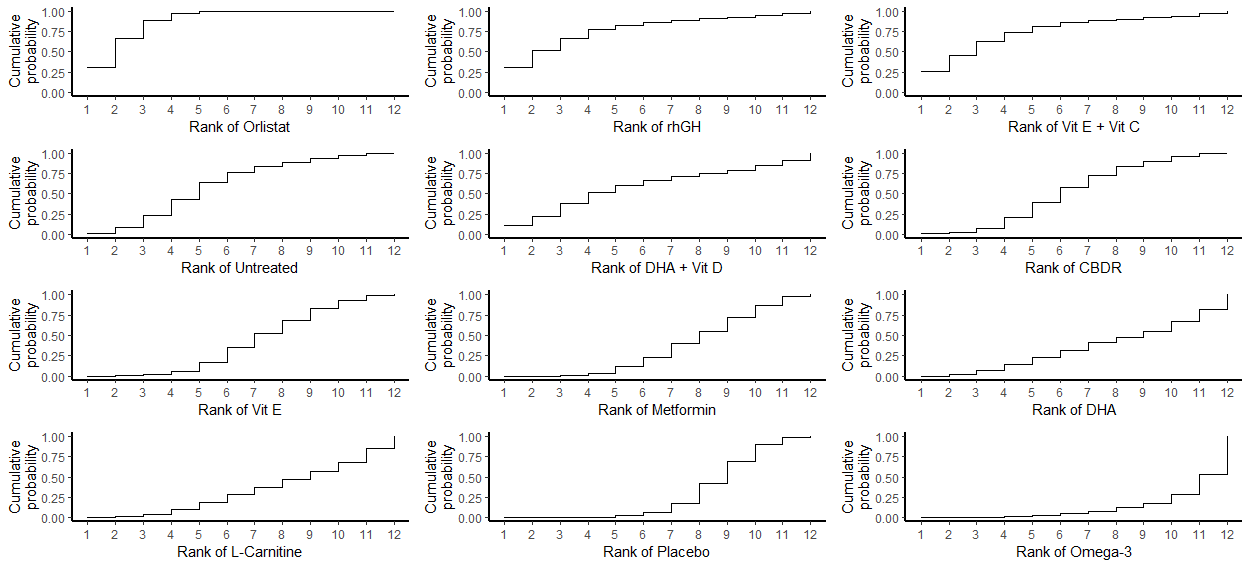


Figure S96 , SUCRA plots showing the cumulative probability of each intervention being among the best for reducing BMI-SDS before sensitivity analysis. Higher curves indicate better rankings.


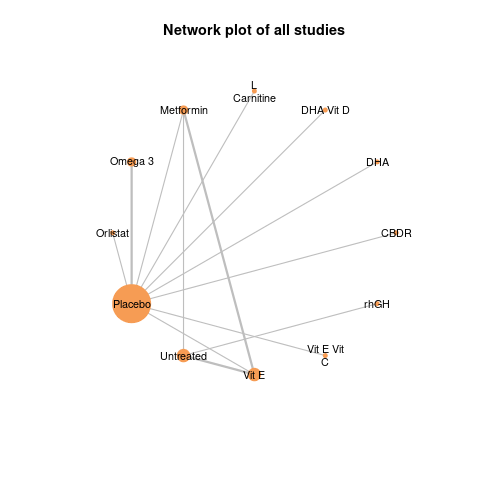


Figure S97 , Network geometry of AST before sensitivity analysis for all included studies, by drug treatments.


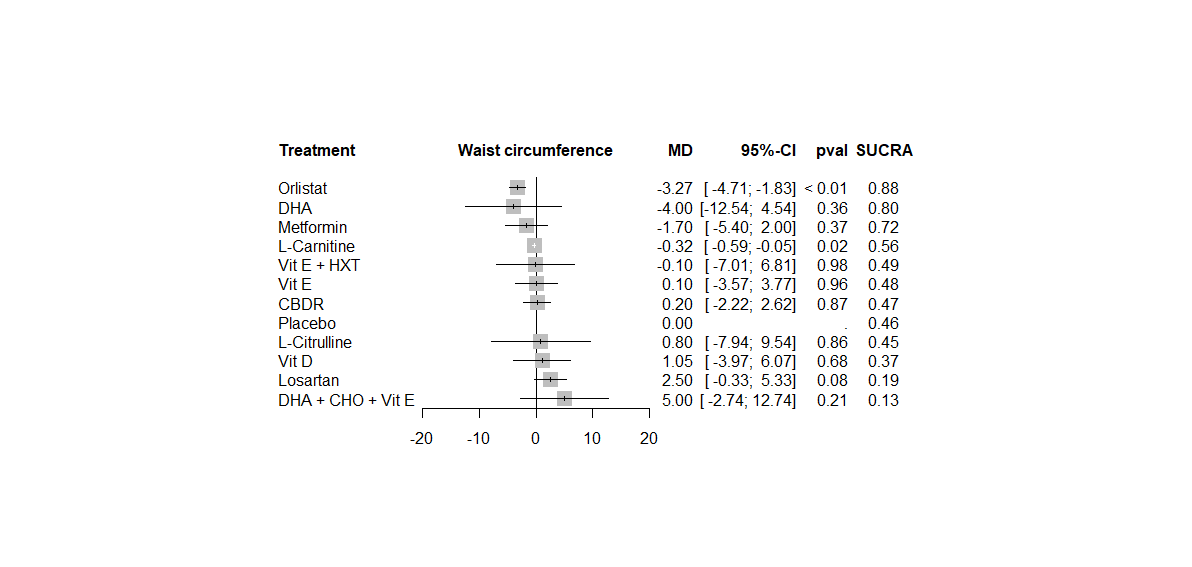


Figure S98, Forest plots for network meta-analysis of Waist circumference.


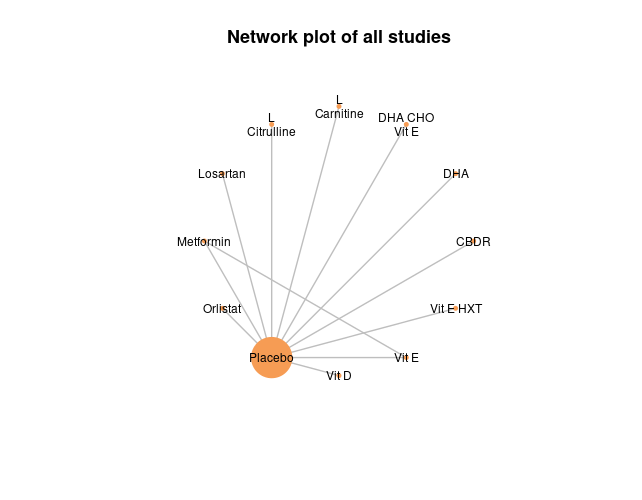


Figure S99, Network geometry of Waist circumference for all included studies, by drug treatments.


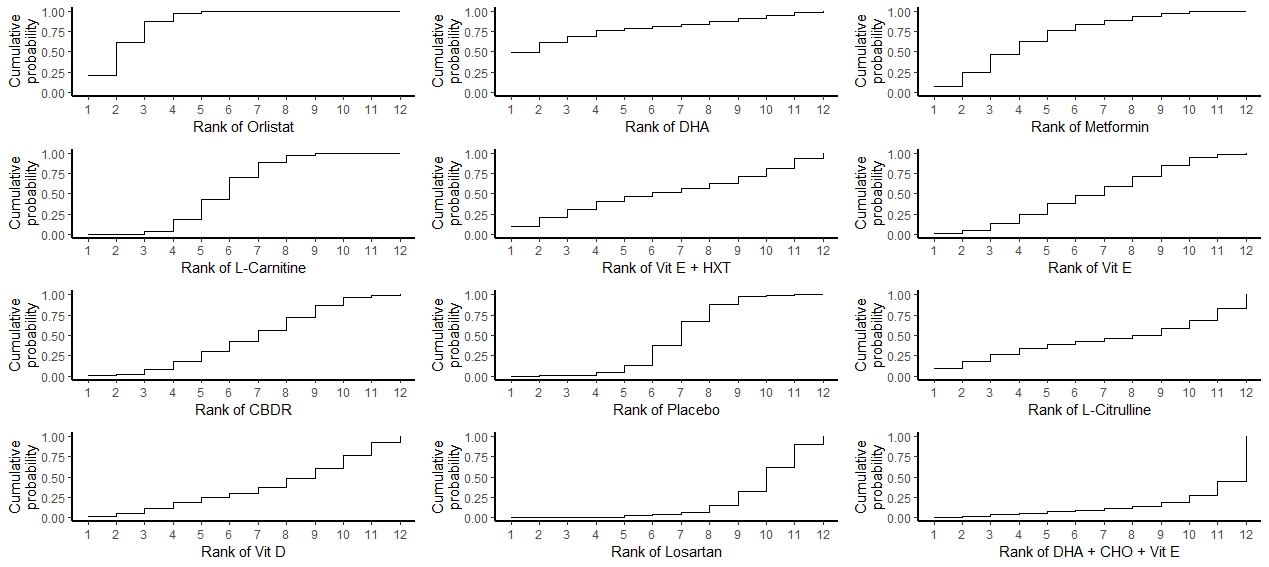


Figure S100, SUCRA plots showing the cumulative probability of each intervention being among the best for reducing Waist circumference. Higher curves indicate better rankings.
